# Supplementary material for: Meta-analysis of epigenome-wide association studies in neonates reveals widespread differential DNA methylation associated with birthweight
Source: Nat Commun. 2019 Apr 23;10:1893. doi: 10.1038/s41467-019-09671-3 (PMC6478731; doi:10.1038/s41467-019-09671-3)
Supplement: Supplementary file 1 — Supplementary Information [file 41467_2019_9671_MOESM1_ESM.pdf]

Supplementary Information

Meta-analysis of epigenome-wide association studies in neonates reveals widespread differential DNA methylation associated with birthweight.

Küpers L.K. *et al*.

Supplementary Figure 1. Manhattan plot showing the main meta-analysis results for associations between DNA methylation and birthweight as a continuous measure
Supplementary Figure 2. Correlation of the point estimates for the secondary meta-analysis of European ancestry only (y-axis) versus main meta-analysis (x-axis)
Supplementary Figures 3.1-3.20. Leave-one-out plots for a random set of 10 (of all 914 birthweight-related) CpGs, showing the association of methylation levels with birthweight as a continuous measure when one of the studies would be omitted from meta-analysis
Correlation of the point estimates from the meta-analysis including preterms, pre-eclampsia and diabetes versus main meta-analysis excluding these participants (x-axis)
Supplementary Figure 4. Correlation of the point estimates from the meta-analysis including preterms, pre-eclampsia and diabetes versus main meta-analysis excluding these participants (x-axis)
Supplementary Note:
Supplementary Funding. Study specific and in alphabetical order
Supplementary Acknowledgements. Study specific and in alphabetical order
Supplementary Methods. Study specific and in alphabetical order
Supplementary References
Supplementary Data to be found in separate Excel file:
Supplementary Data 1. Characteristics of the participating studies in the main meta-analysis (A), the look-up in blood samples taken later in life (B) and the sensitivity model without exclusions of preterm births, pre-eclampsia or maternal diabetes (C)
Supplementary Data 2. Main meta-analysis results from 8170 CpGs with FDR siginificant association between methylation levels and continuous birth weight in grams (first 1029 CpGs are Bonferroni significant, $P<1.06*10^{-7}$)
Supplementary Data 3. Meta-analysis results from 914 CpGs for the association between methylation levels and continuous birth weight in grams, in three separate ancestry groups
Supplementary Data 4. Meta-analysis results from 52 CpGs with association between methylation levels and high versus normal birth weight, after excluding preterm births, pre-eclampsia and maternal diabetes ($P<1.06*10^{-7}$)
Supplementary Data 5. Results for meta-analyses without exclusion of preterm births, pre-eclampsia or maternal diabetes; association of DNA methylation with continuous birthweight in grams (n=5414), low (n=178) versus normal (n=4197) birthweight, and high (n=1039) versus normal (n=4197) birthweight
Supplementary Data 6. Comparison of 914 robust CpGs with two lists of SNP-influenced CpG probes and results from the dip test for multimodality
Supplementary Data 7. Meta-analysis results between methylation levels and continuous birth weight in grams from look-up in methylation samples in childhood, adolescence and adulthood: results from 914 robust CpGs in main meta-analysis
Supplementary Data 8. Overlap of CpGs that are associated with birthweight as well as maternal smoking during pregnancy or maternal body mass index
Supplementary Data 9. Comparison of 914 birthweight-related CpG sites with metastable epialleles and CpGs associated with genomic imprinting
Supplementary Data 10. Birthweight-related CpGs sites that are located +/- 2Mb of birthweight-related SNPs from a recently published fetal GWAS meta-analysis (Horikoshi et al.)
Supplementary Data 11. Birthweight-related CpGs sites that are located +/- 2Mb of birthweight-related SNPs from a recently published maternal GWAS meta-analysis (Beaumont et al.)
Supplementary Data 12. The 98 expression quantitative trait methylation (cis-eQTM) sites for 82 of the 914 birthweight associated CpG sites
Supplementary Data 13. Associations between gene expression and methylation in INMA and the Gambia (FDR<0.05)
Supplementary Data 14. Gene Ontology (GO) term enrichment for birthweight-related CpGs

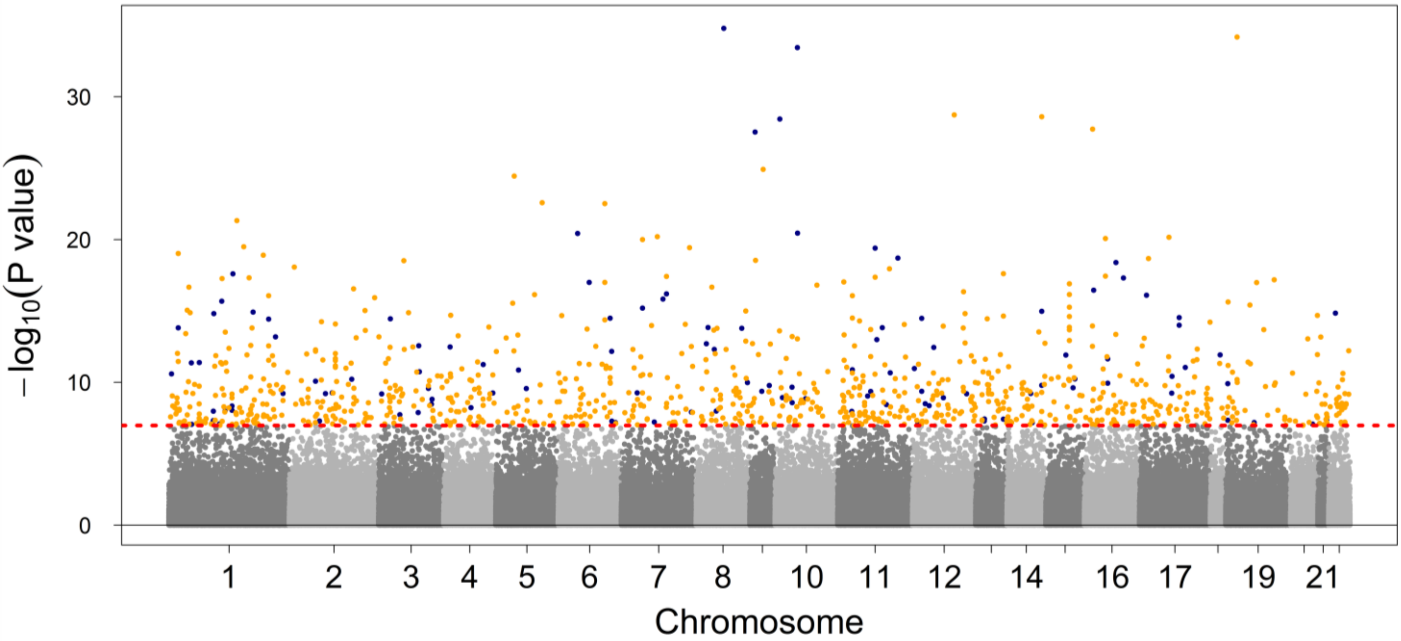

Supplementary Figure 1. Manhattan plot showing the main meta-analysis results for associations between DNA methylation and birthweight as a continuous measure

The red dashed line shows the Bonferroni-corrected significance threshold for multiple testing ($p<1.06x10^{-7}$). Highlighted in orange are the 914 CpGs with $p<1.06x10^{-7}$ and $I^2 \leq 50\%$ and highlighted in blue are the 115 CpGs with $p<1.06x10^{-7}$ and $I^2>50\%$.

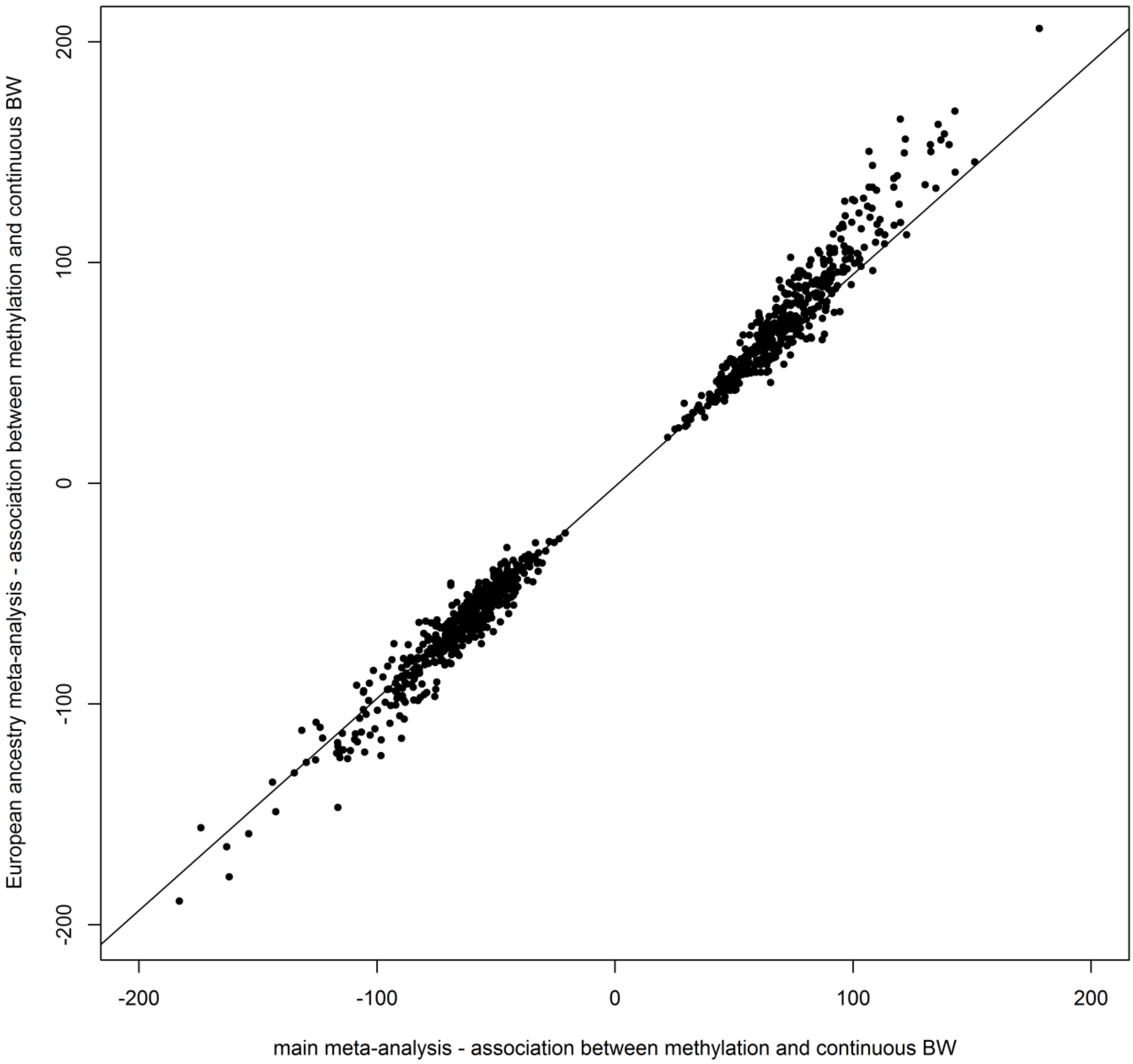

Supplementary Figure 2. Correlation of the point estimates for the secondary meta-analysis of European ancestry only (y-axis) versus main meta-analysis (x-axis)

Correlation of the point estimates for a secondary meta-analysis of 6,023 participants from 17 studies who were all of European ancestry (y-axis) with point estimates for the main meta-analysis with 8,809 participants from 24 studies of mixed ancestry (x-axis).

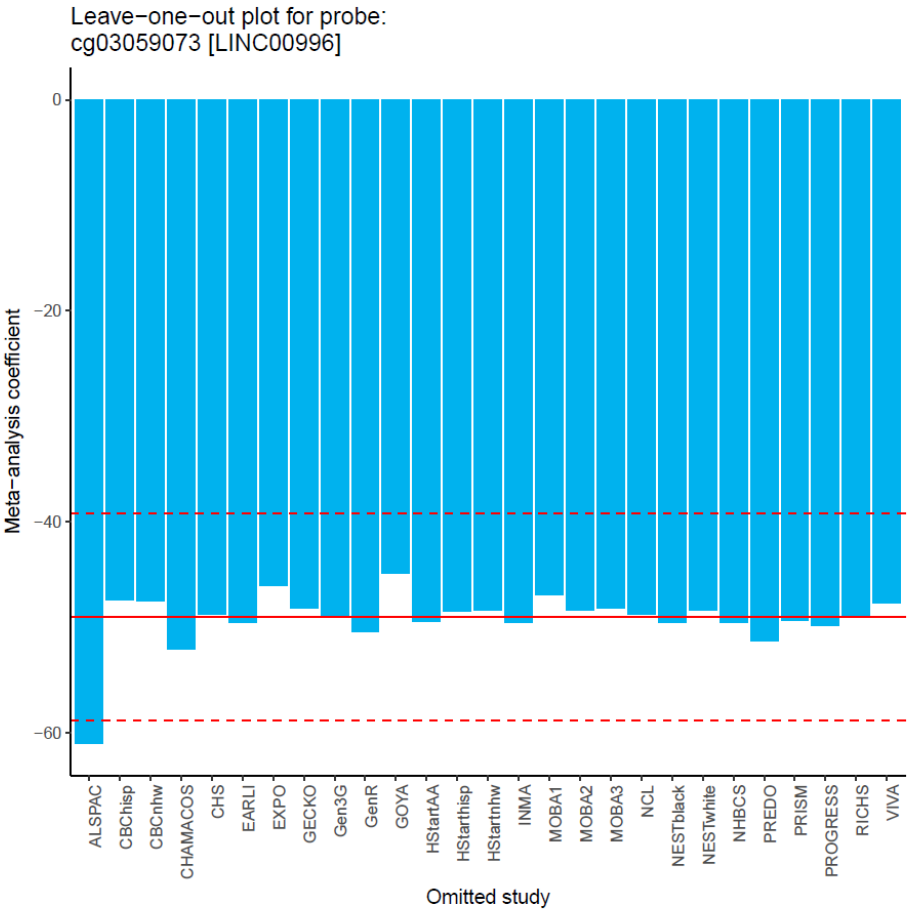
Leave−one−out plot for probe:
cg03059073 [LINC00996]
Meta−analysis coefficient
0
−20
−40
−60
ALSPAC
CBChisp
CBCnhw
CHAMACOS
CHS
EARLI
EXPO
GECKO
Gen3G
GenR
GOYA
HStartAA
HStarthisp
HStartnhw
INMA
MOBA1
MOBA2
MOBA3
NCL
NESTblack
NESTwhite
NHBCS
PREDO
PRISM
PROGRESS
RICHS
VIVA
Omitted study

3.1

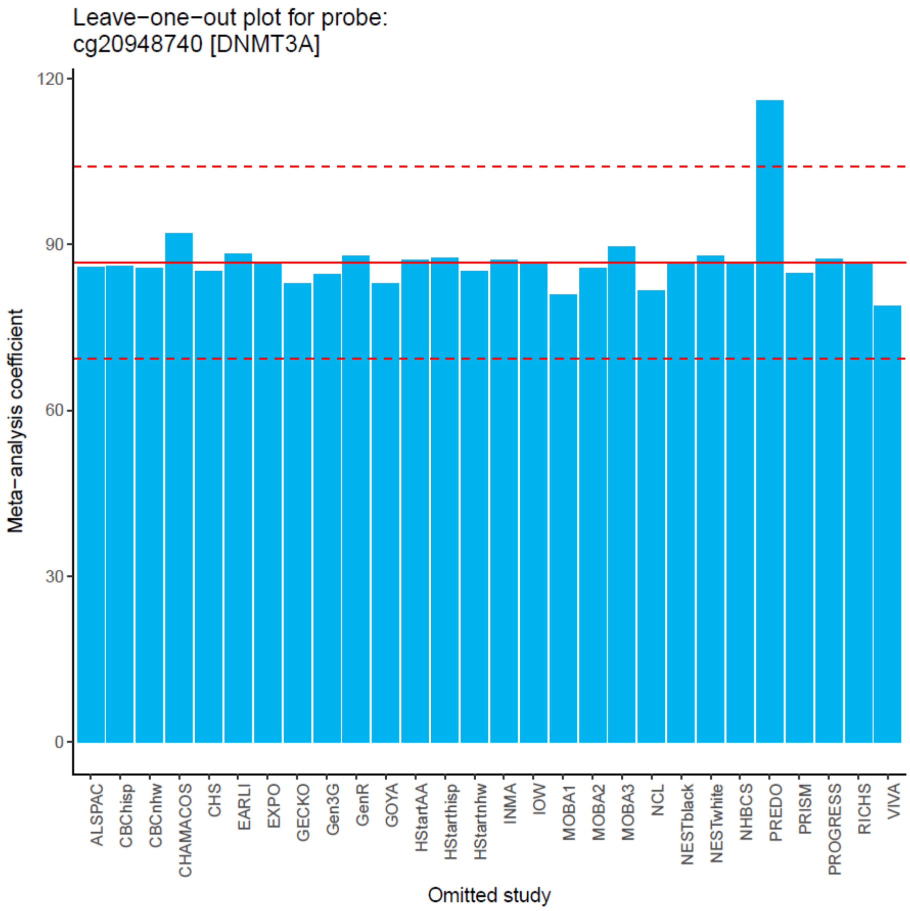
Leave−one−out plot for probe:
cg20948740 [DNMT3A]
Meta−analysis coefficient
120
90
60
30
0
ALSPAC
CBChisp
CBCnhw
CHAMACOS
CHS
EARLI
EXPO
GECKO
Gen3G
GenR
GOYA
HStartAA
HStarthisp
HStartnhw
INMA
IOW
MOBA1
MOBA2
MOBA3
NCL
NESTblack
NESTwhite
NHBCS
PREDO
PRISM
PROGRESS
RICHS
VIVA
Omitted study

3.2

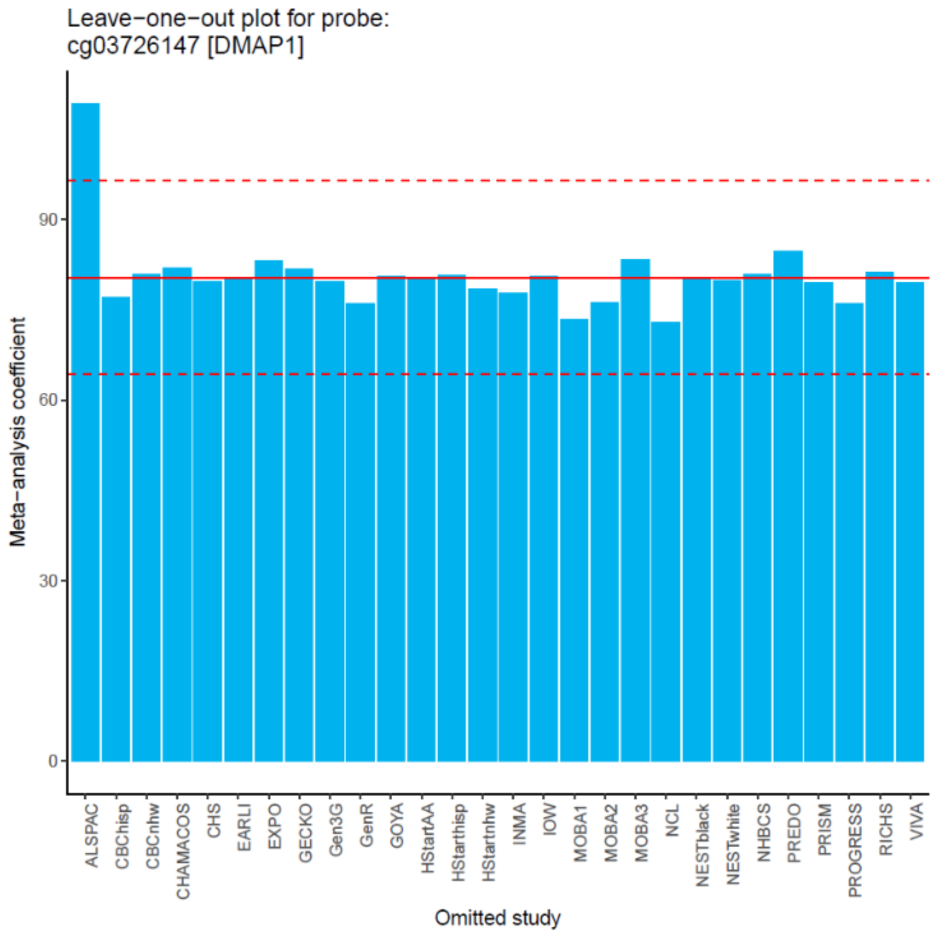
Leave−one−out plot for probe:
cg03726147 [DMAP1]
Meta−analysis coefficient
0
30
60
90
ALSPAC
CBChisp
CBCnhw
CHAMACOS
CHS
EARLI
EXPO
GECKO
Gen3G
GenR
GOYA
HStartAA
HStarthisp
HStartnhw
INMA
IOW
MOBA1
MOBA2
MOBA3
NCL
NESTblack
NESTwhite
NHBCS
PREDO
PRISM
PROGRESS
RICHS
VIVA
Omitted study

3.3

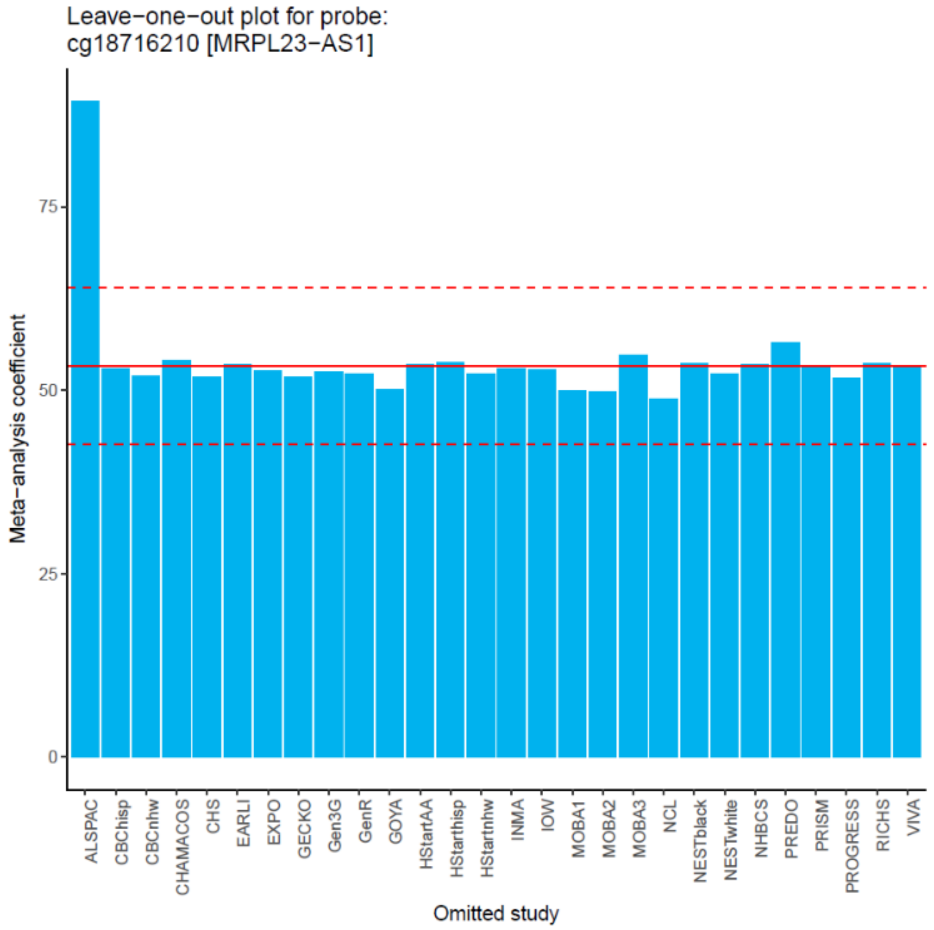
Leave−one−out plot for probe:
cg18716210 [MRPL23−AS1]
Meta−analysis coefficient
0
25
50
75
ALSPAC
CBChisp
CBCnhw
CHAMACOS
CHS
EARLI
EXPO
GECKO
Gen3G
GenR
GOYA
HStartAA
HStarthisp
HStartnhw
INMA
IOW
MOBA1
MOBA2
MOBA3
NCL
NESTblack
NESTwhite
NHBCS
PREDO
PRISM
PROGRESS
RICHS
VIVA
Omitted study

3.4

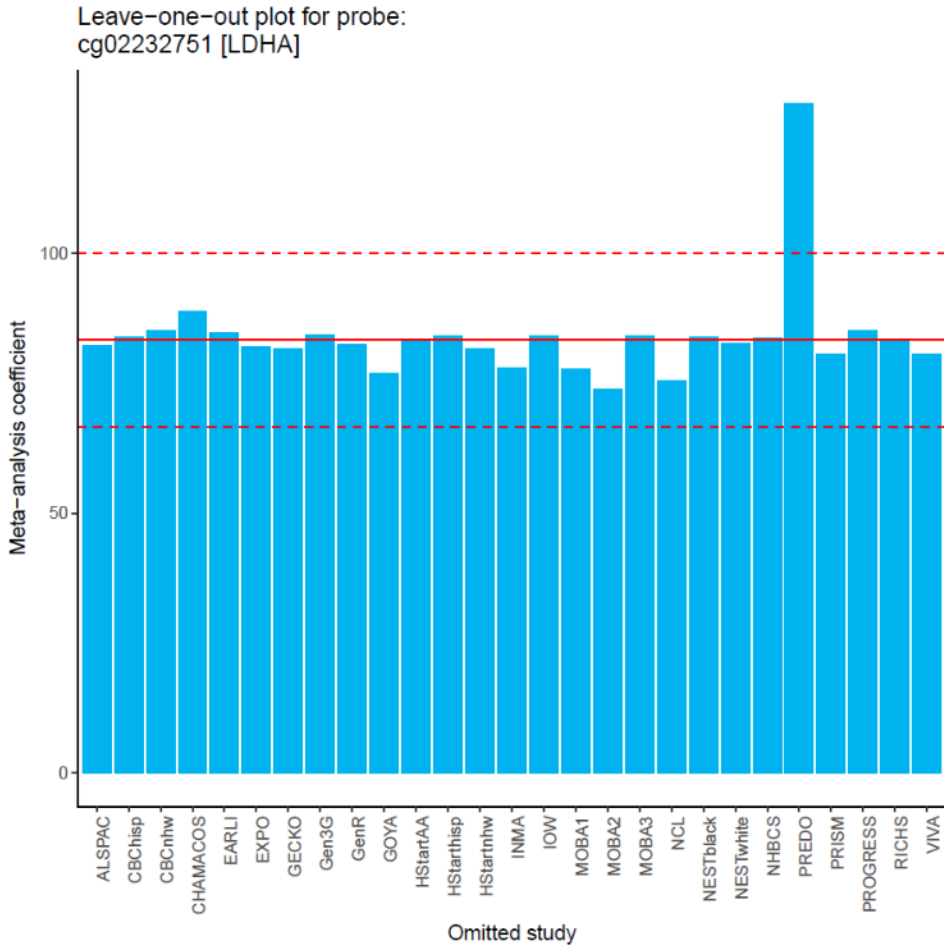
Leave−one−out plot for probe:
cg02232751 [LDHA]
Meta−analysis coefficient
100
50
0
ALSPAC
CBChisp
CBCnhw
CHAMACOS
EARLI
EXPO
GECKO
Gen3G
GenR
GOYA
HStartAA
HStarthisp
HStartnhw
INMA
IOW
MOBA1
MOBA2
MOBA3
NCL
NESTblack
NESTwhite
NHBCS
PREDO
PRISM
PROGRESS
RICHS
VIVA
Omitted study

3.5

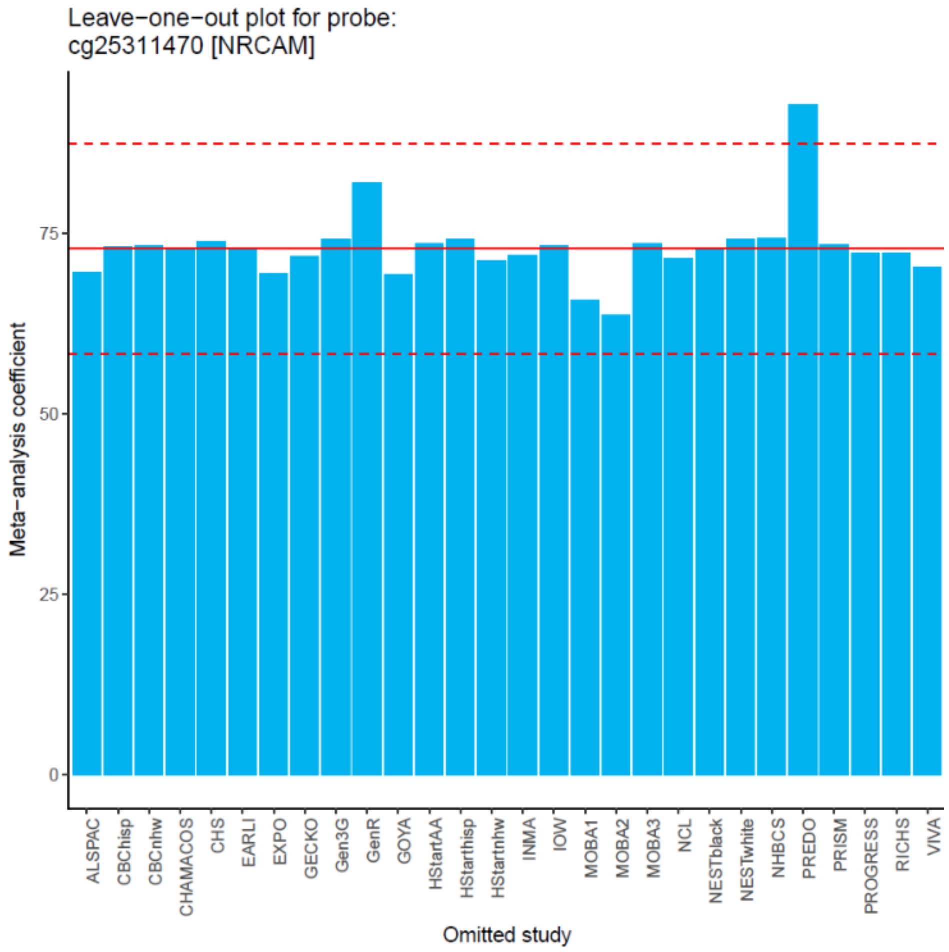
Leave−one−out plot for probe:
cg25311470 [NRCAM]
Meta−analysis coefficient
75
50
25
0
ALSPAC
CBChisp
CBCnhw
CHAMACOS
CHS
EARLI
EXPO
GECKO
Gen3G
GenR
GOYA
HStartAA
HStarthisp
HStartnhw
INMA
IOW
MOBA1
MOBA2
MOBA3
NCL
NESTblack
NESTwhite
NHBCS
PREDO
PRISM
PROGRESS
RICHS
VIVA
Omitted study

3.6

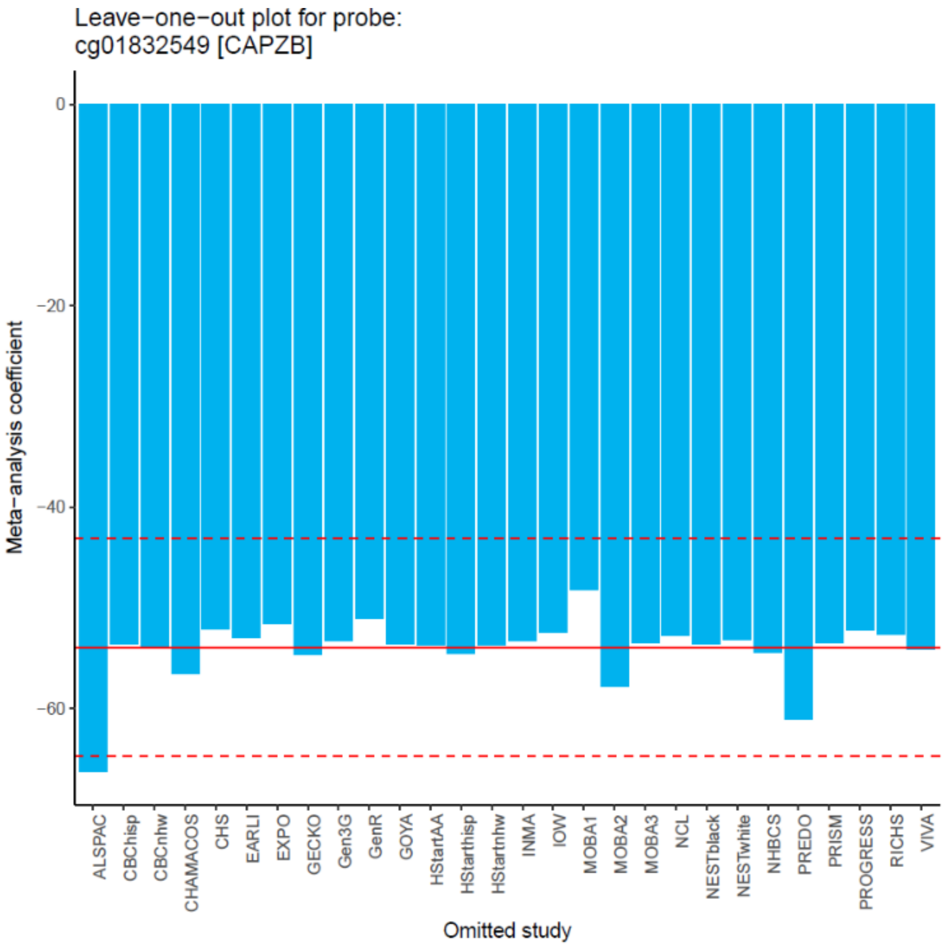

Leave−one−out plot for probe:
cg01832549 [CAPZB]
Meta−analysis coefficient
0
−20
−40
−60
ALSPAC
CBChisp
CBCnhw
CHAMACOS
CHS
EARLI
EXPO
GECKO
Gen3G
GenR
GOYA
HStartAA
HStarthisp
HStartnhw
INMA
IOW
MOBA1
MOBA2
MOBA3
NCL
NESTblack
NESTwhite
NHBCS
PREDO
PRISM
PROGRESS
RICHS
VIVA
Omitted study

3.7

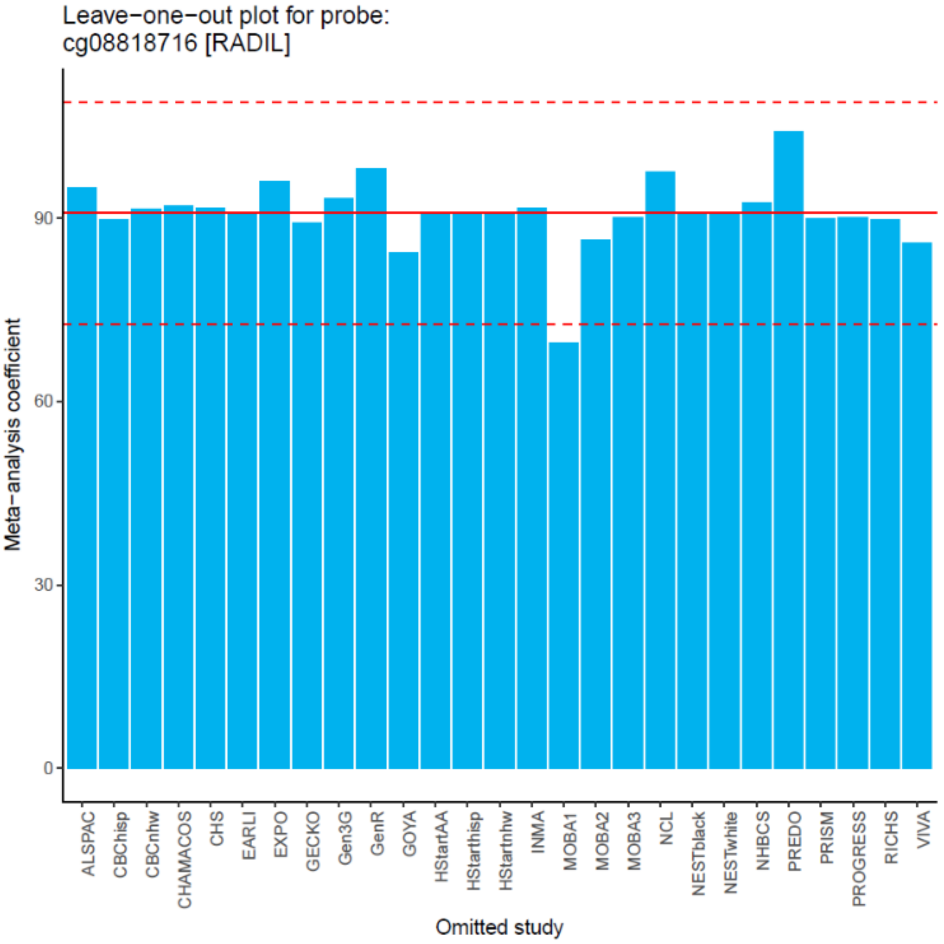

Leave−one−out plot for probe:
cg08818716 [RADIL]
Meta−analysis coefficient
90
60
30
0
ALSPAC
CBChisp
CBCnhw
CHAMACOS
CHS
EARLI
EXPO
GECKO
Gen3G
GenR
GOYA
HStartAA
HStarthisp
HStartnhw
INMA
MOBA1
MOBA2
MOBA3
NCL
NESTblack
NESTwhite
NHBCS
PREDO
PRISM
PROGRESS
RICHS
VIVA
Omitted study

3.8

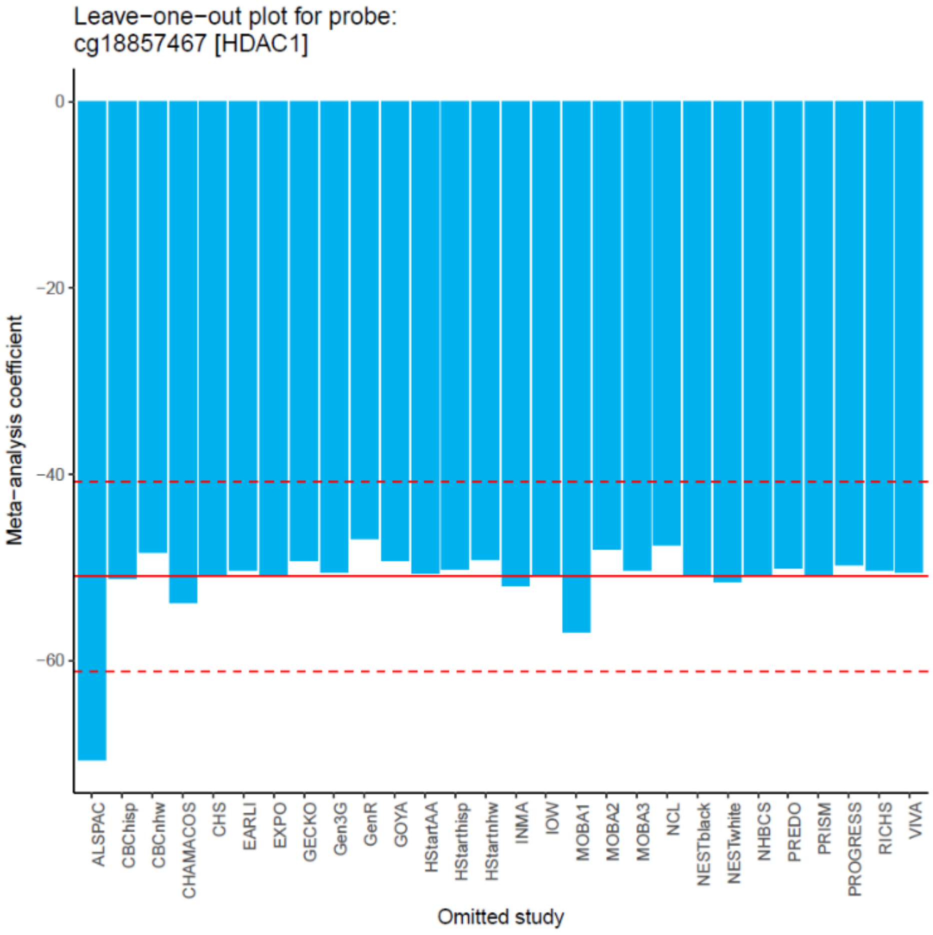
Leave−one−out plot for probe:
cg18857467 [HDAC1]
Meta−analysis coefficient
0
−20
−40
−60
ALSPAC
CBChisp
CBCnhw
CHAMACOS
CHS
EARLI
EXPO
GECKO
Gen3G
GenR
GOYA
HStartAA
HStarthisp
HStartnhw
INMA
IOW
MOBA1
MOBA2
MOBA3
NCL
NESTblack
NESTwhite
NHBCS
PREDO
PRISM
PROGRESS
RICHS
VIVA
Omitted study

3.9

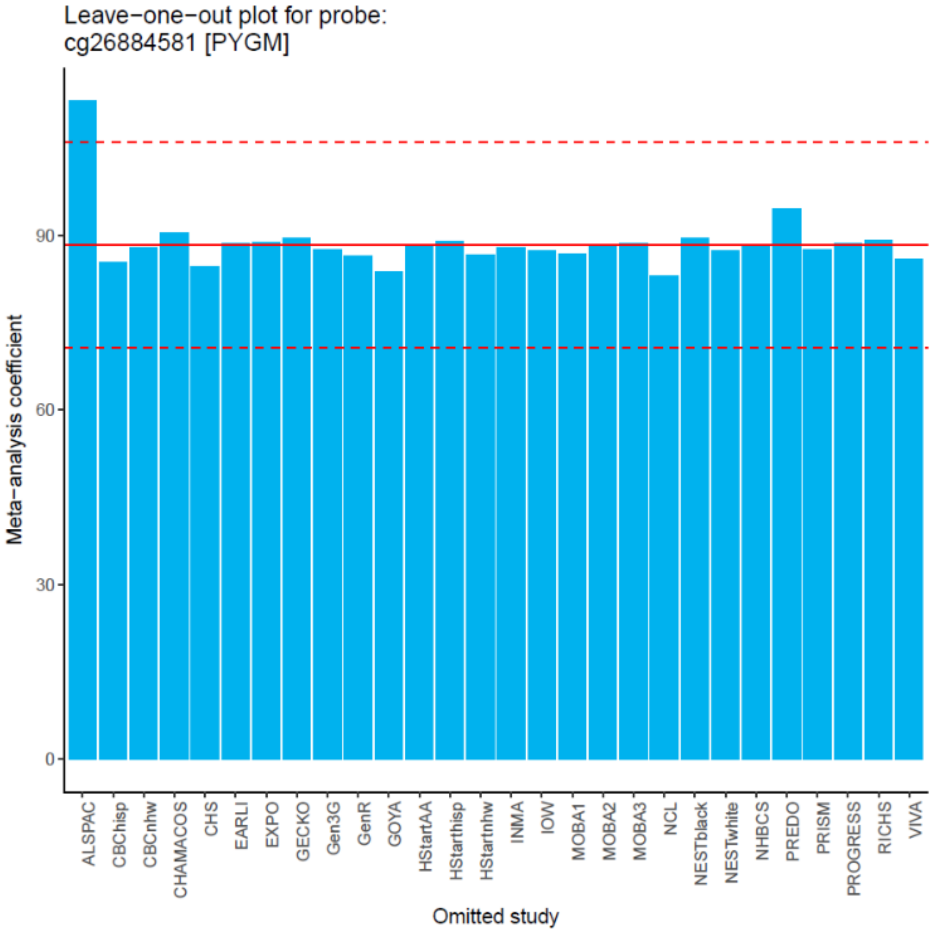
Leave−one−out plot for probe:
cg26884581 [PYGM]
Meta−analysis coefficient
90
60
30
0
ALSPAC
CBChisp
CBCnhw
CHAMACOS
CHS
EARLI
EXPO
GECKO
Gen3G
GenR
GOYA
HStartAA
HStarthisp
HStartnhw
INMA
IOW
MOBA1
MOBA2
MOBA3
NCL
NESTblack
NESTwhite
NHBCS
PREDO
PRISM
PROGRESS
RICHS
VIVA
Omitted study

3.10

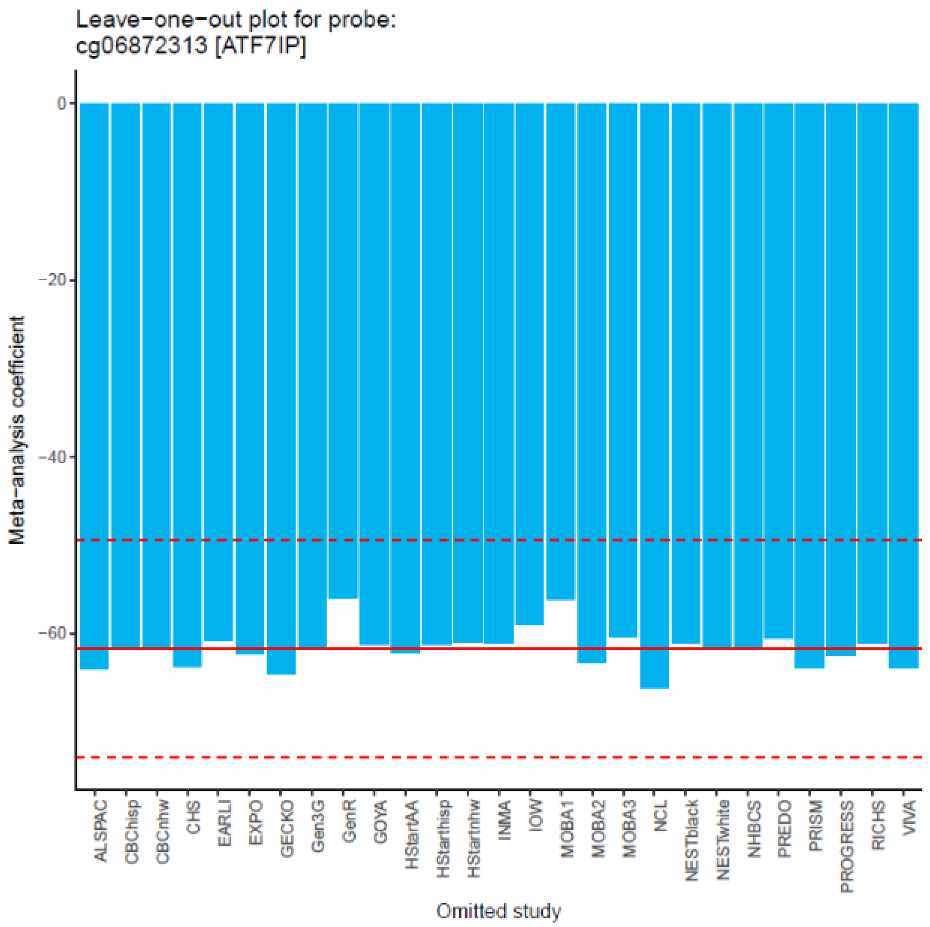

Leave−one−out plot for probe:
cg06872313 [ATF7IP]
Meta−analysis coefficient
0
−20
−40
−60
ALSPAC
CBChisp
CBCnhw
CHS
EARLI
EXPO
GECKO
Gen3G
GenR
GOYA
HStartAA
HStarthisp
HStartnhw
INMA
IOW
MOBA1
MOBA2
MOBA3
NCL
NESTblack
NESTwhite
NHBCS
PREDO
PRISM
PROGRESS
RICHS
VIVA
Omitted study

3.11

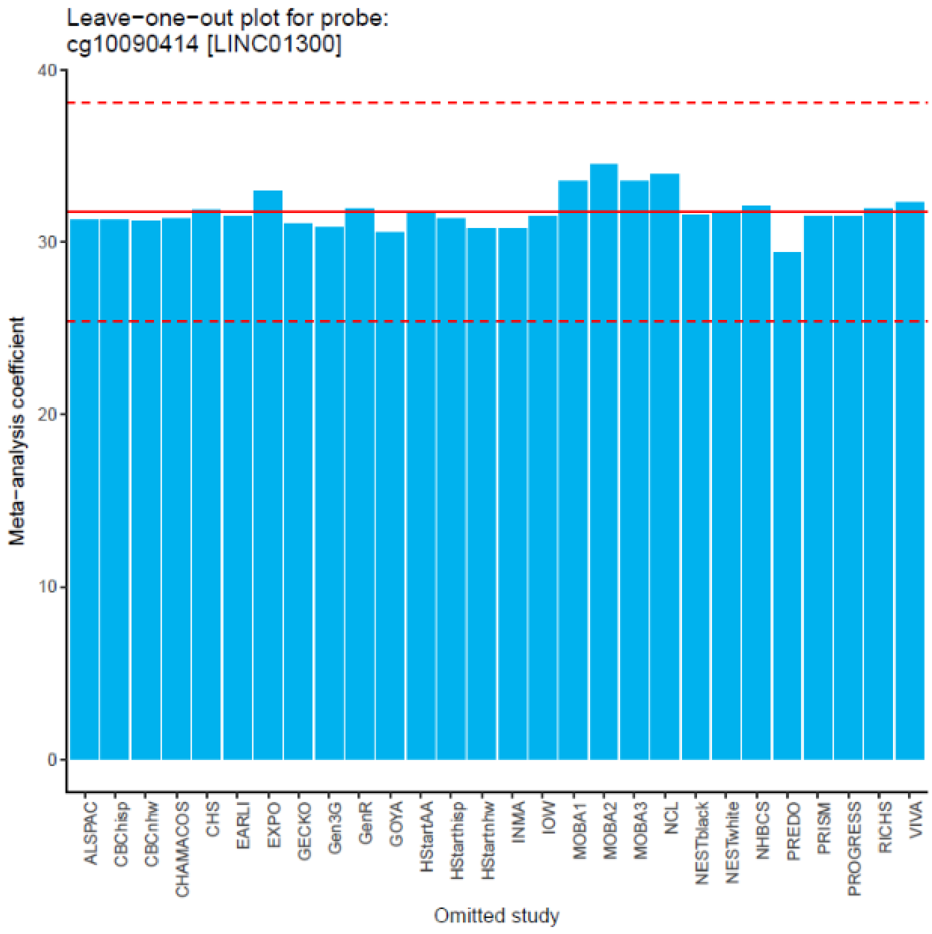

Leave−one−out plot for probe:
cg10090414 [LINC01300]
Meta−analysis coefficient
40
30
20
10
0
ALSPAC
CBChisp
CBCnhw
CHAMACOS
CHS
EARLI
EXPO
GECKO
Gen3G
GenR
GOYA
HStartAA
HStarthisp
HStartnhw
INMA
IOW
MOBA1
MOBA2
MOBA3
NCL
NESTblack
NESTwhite
NHBCS
PREDO
PRISM
PROGRESS
RICHS
VIVA
Omitted study

3.12

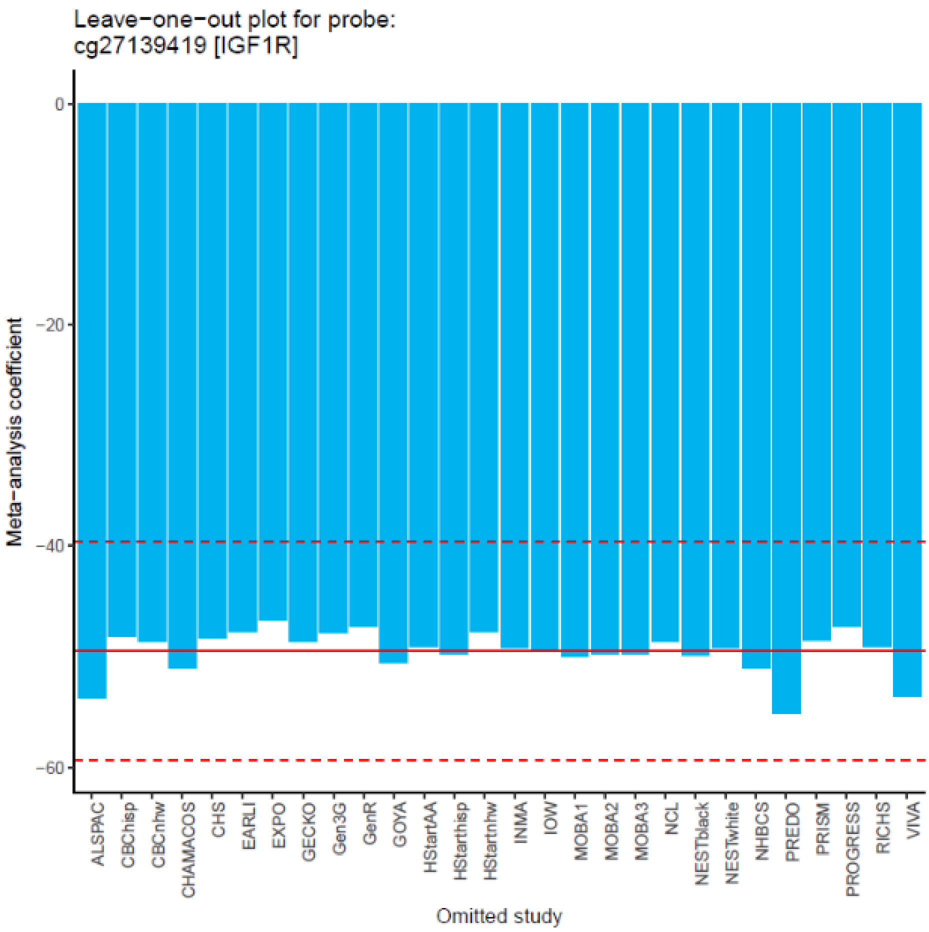
Leave−one−out plot for probe:
cg27139419 [IGF1R]
Meta−analysis coefficient
0
-20
-40
-60
ALSPAC
CBChisp
CBCnhw
CHAMACOS
CHS
EARLI
EXPO
GECKO
Gen3G
GenR
GOYA
HStartAA
HStarthisp
HStartnhw
INMA
IOW
MOBA1
MOBA2
MOBA3
NCL
NESTblack
NESTwhite
NHBCS
PREDO
PRISM
PROGRESS
RICHS
VIVA
Omitted study

3.13

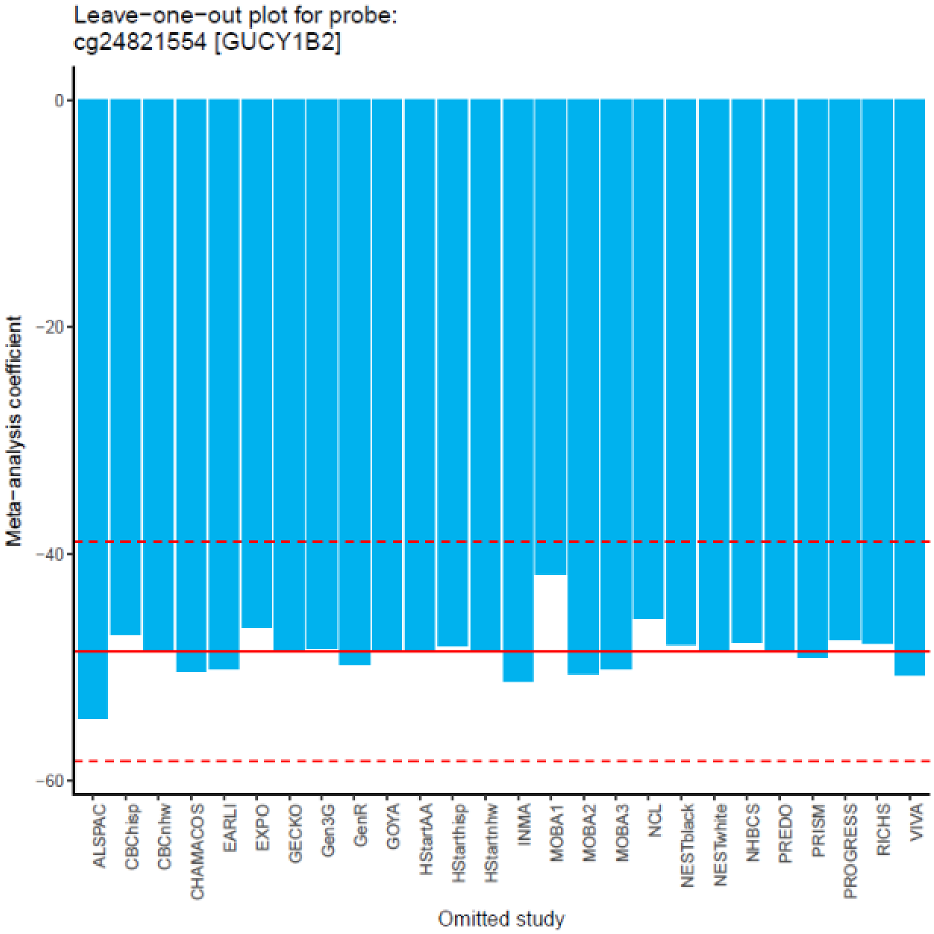
Leave−one−out plot for probe:
cg24821554 [GUCY1B2]
Meta−analysis coefficient
0
-20
-40
-60
ALSPAC
CBChisp
CBCnhw
CHAMACOS
EARLI
EXPO
GECKO
Gen3G
GenR
GOYA
HStartAA
HStarthisp
HStartnhw
INMA
MOBA1
MOBA2
MOBA3
NCL
NESTblack
NESTwhite
NHBCS
PREDO
PRISM
PROGRESS
RICHS
VIVA
Omitted study

3.14

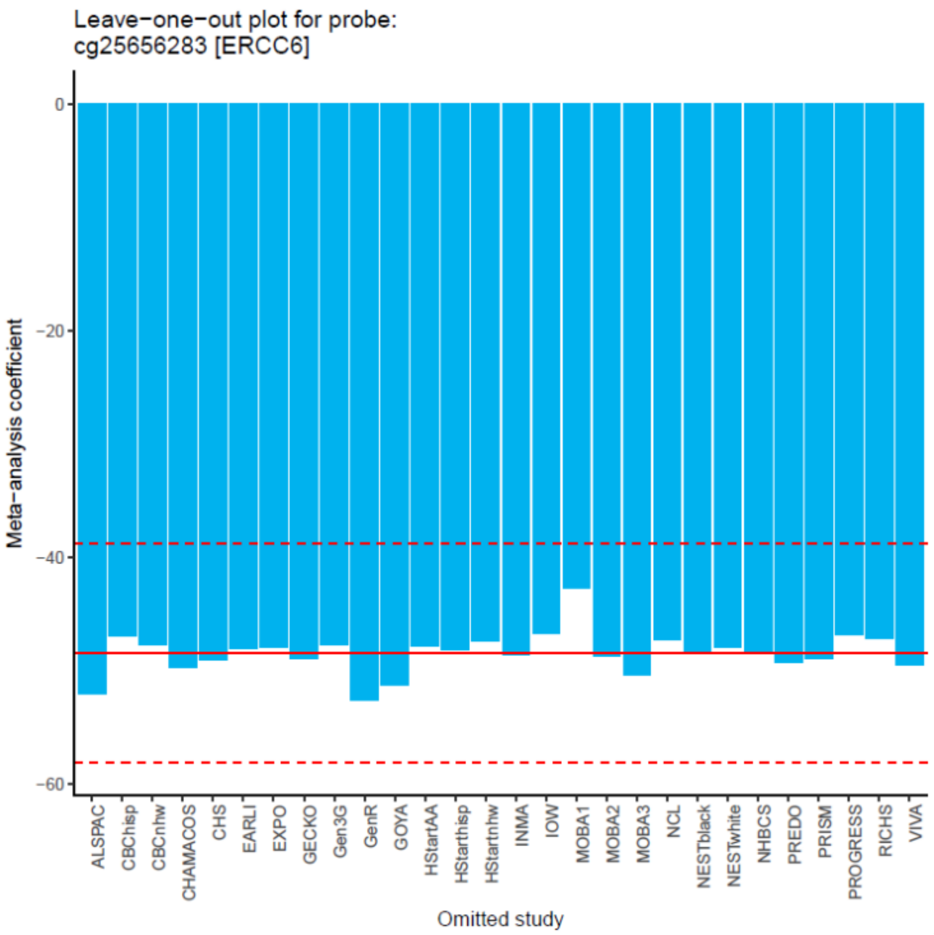
Leave−one−out plot for probe:
cg25656283 [ERCC6]
Meta−analysis coefficient
0
−20
−40
−60
ALSPAC
CBChisp
CBCnhw
CHAMACOS
CHS
EARLI
EXPO
GECKO
Gen3G
GenR
GOYA
HStartAA
HStarthisp
HStartnhw
INMA
IOW
MOBA1
MOBA2
MOBA3
NCL
NESTblack
NESTwhite
NHBCS
PREDO
PRISM
PROGRESS
RICHS
VIVA
Omitted study

3.15

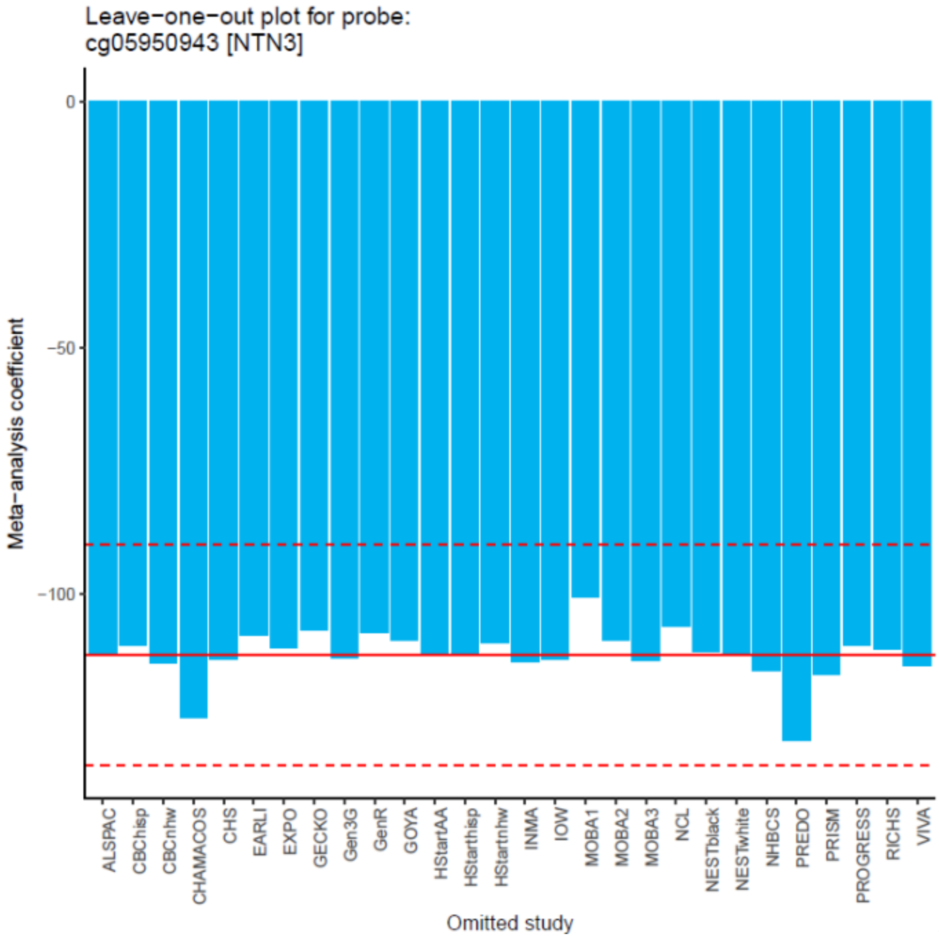
Leave−one−out plot for probe:
cg05950943 [NTN3]
Meta−analysis coefficient
0
−50
−100
ALSPAC
CBChisp
CBCnhw
CHAMACOS
CHS
EARLI
EXPO
GECKO
Gen3G
GenR
GOYA
HStartAA
HStarthisp
HStartnhw
INMA
IOW
MOBA1
MOBA2
MOBA3
NCL
NESTblack
NESTwhite
NHBCS
PREDO
PRISM
PROGRESS
RICHS
VIVA
Omitted study

3.16

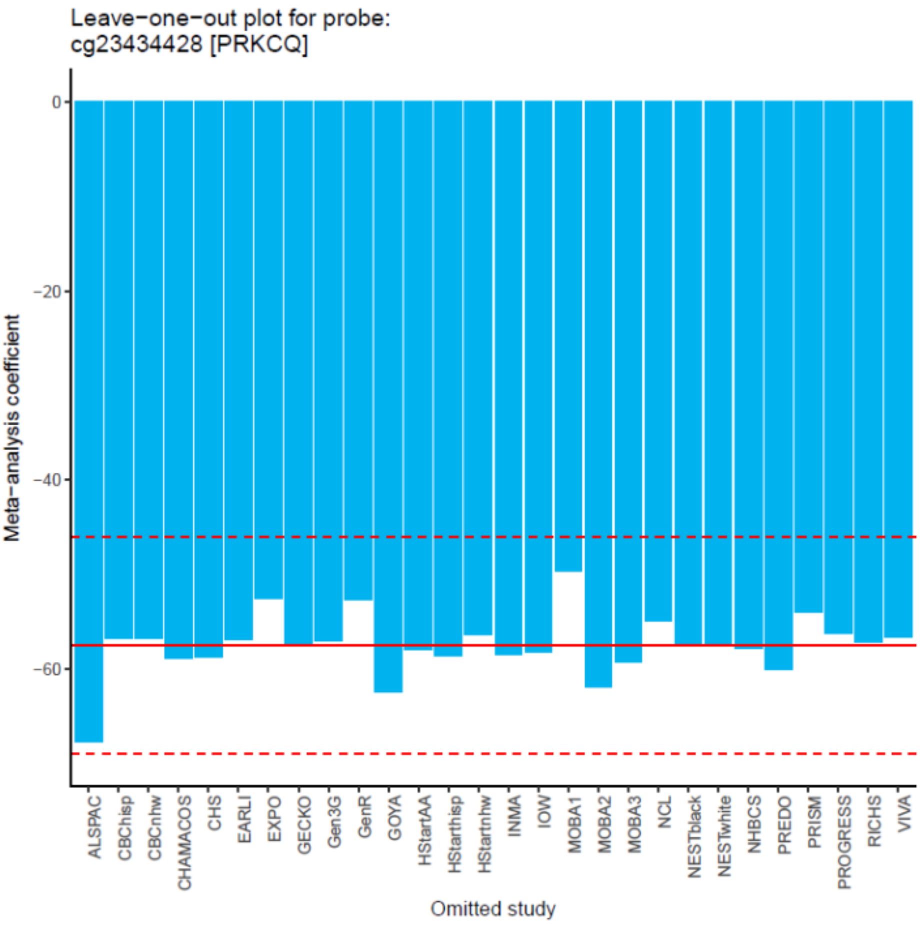

Leave−one−out plot for probe:
cg23434428 [PRKCQ]
Meta−analysis coefficient
0
−20
−40
−60
ALSPAC
CBChisp
CBCnhw
CHAMACOS
CHS
EARLI
EXPO
GECKO
Gen3G
GenR
GOYA
HStartAA
HStarthisp
HStartnhw
INMA
IOW
MOBA1
MOBA2
MOBA3
NCL
NESTblack
NESTwhite
NHBCS
PREDO
PRISM
PROGRESS
RICHS
VIVA
Omitted study

3.17

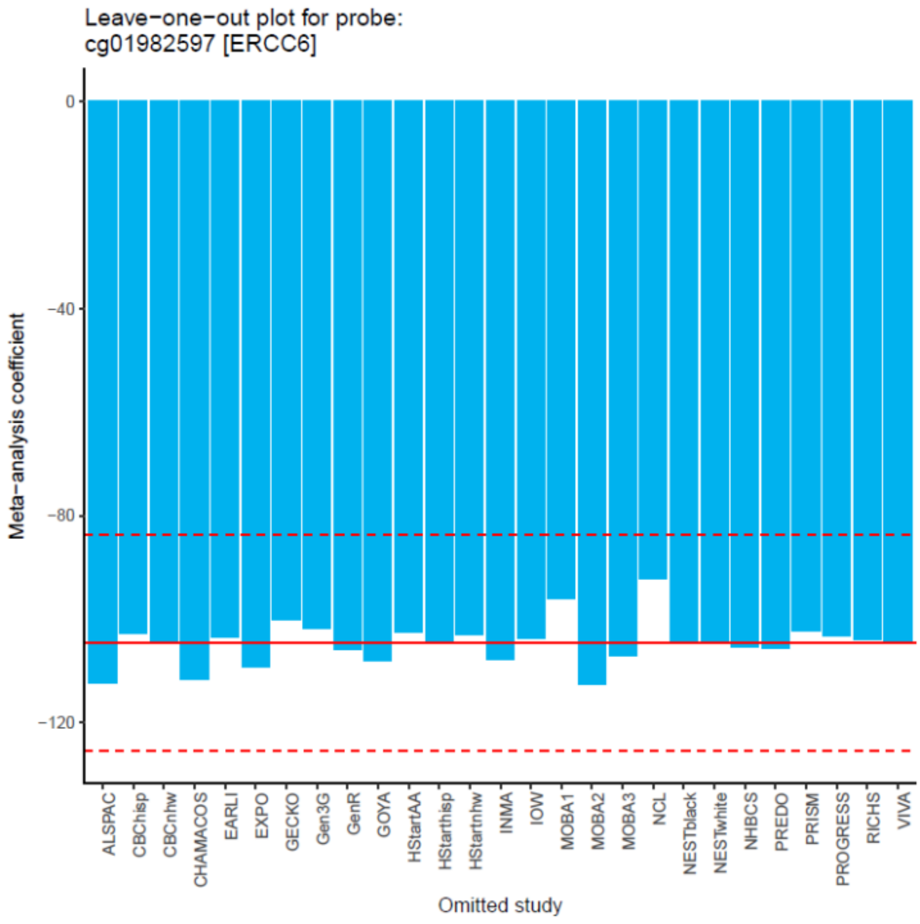

Leave−one−out plot for probe:
cg01982597 [ERCC6]
Meta−analysis coefficient
0
−40
−80
−120
ALSPAC
CBChisp
CBCnhw
CHAMACOS
EARLI
EXPO
GECKO
Gen3G
GenR
GOYA
HStartAA
HStarthisp
HStartnhw
INMA
IOW
MOBA1
MOBA2
MOBA3
NCL
NESTblack
NESTwhite
NHBCS
PREDO
PRISM
PROGRESS
RICHS
VIVA
Omitted study

3.18

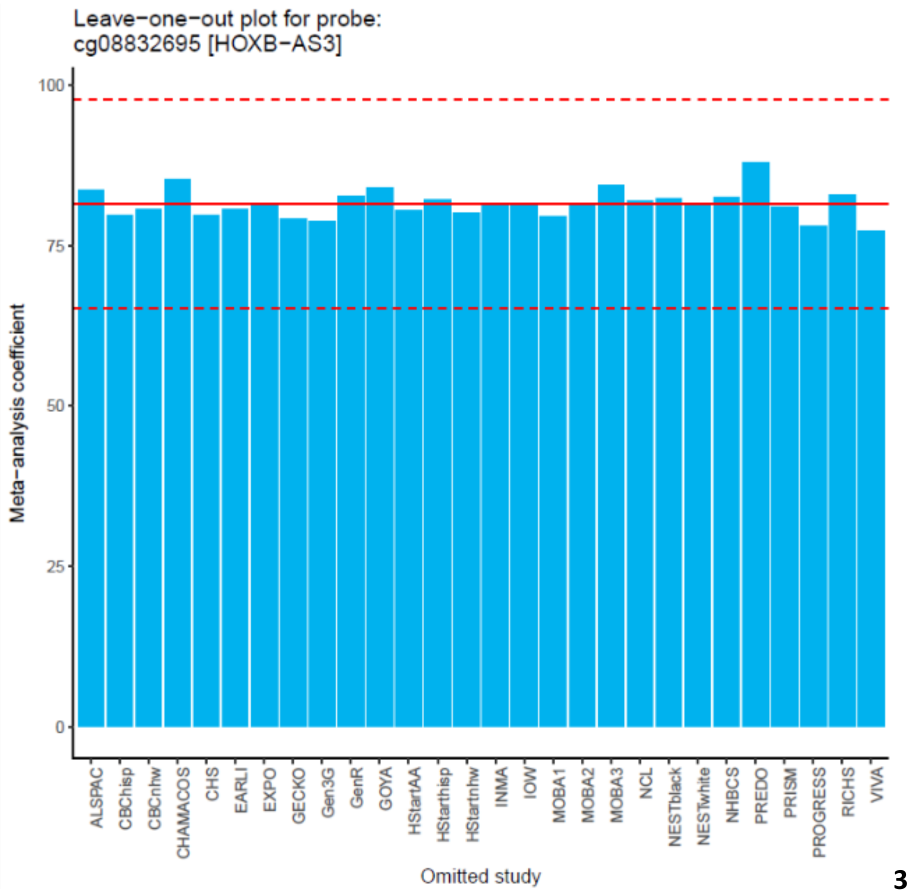

3.19

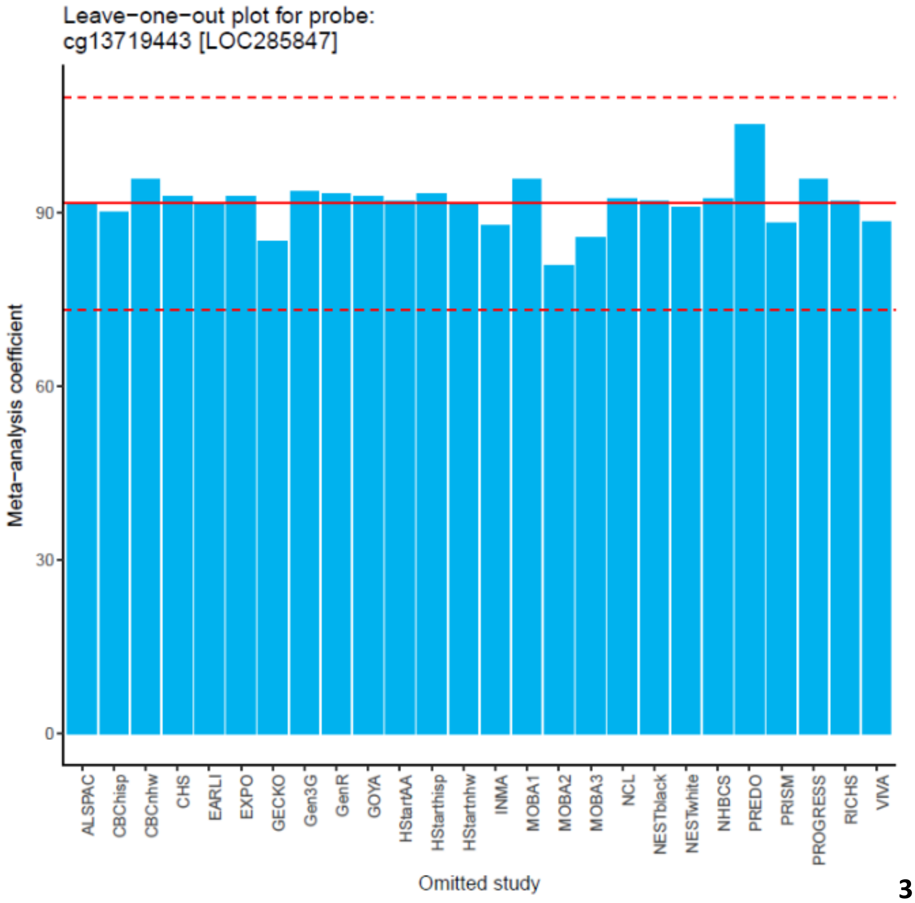

3.20

Supplementary Figures3.1-3.20. Leave-one-out plots for a random 10 (of all 914 birthweight-related) CpGs, showing the association of methylation levels with birthweight as a continuous measure, where removal of one study changed the result by 20% or more (3.1-3.10) and a random 10 where this was not the case (3.11-3.20). The name on the x-axis, representing each bar, is the study that was omitted for that specific meta-analysis.

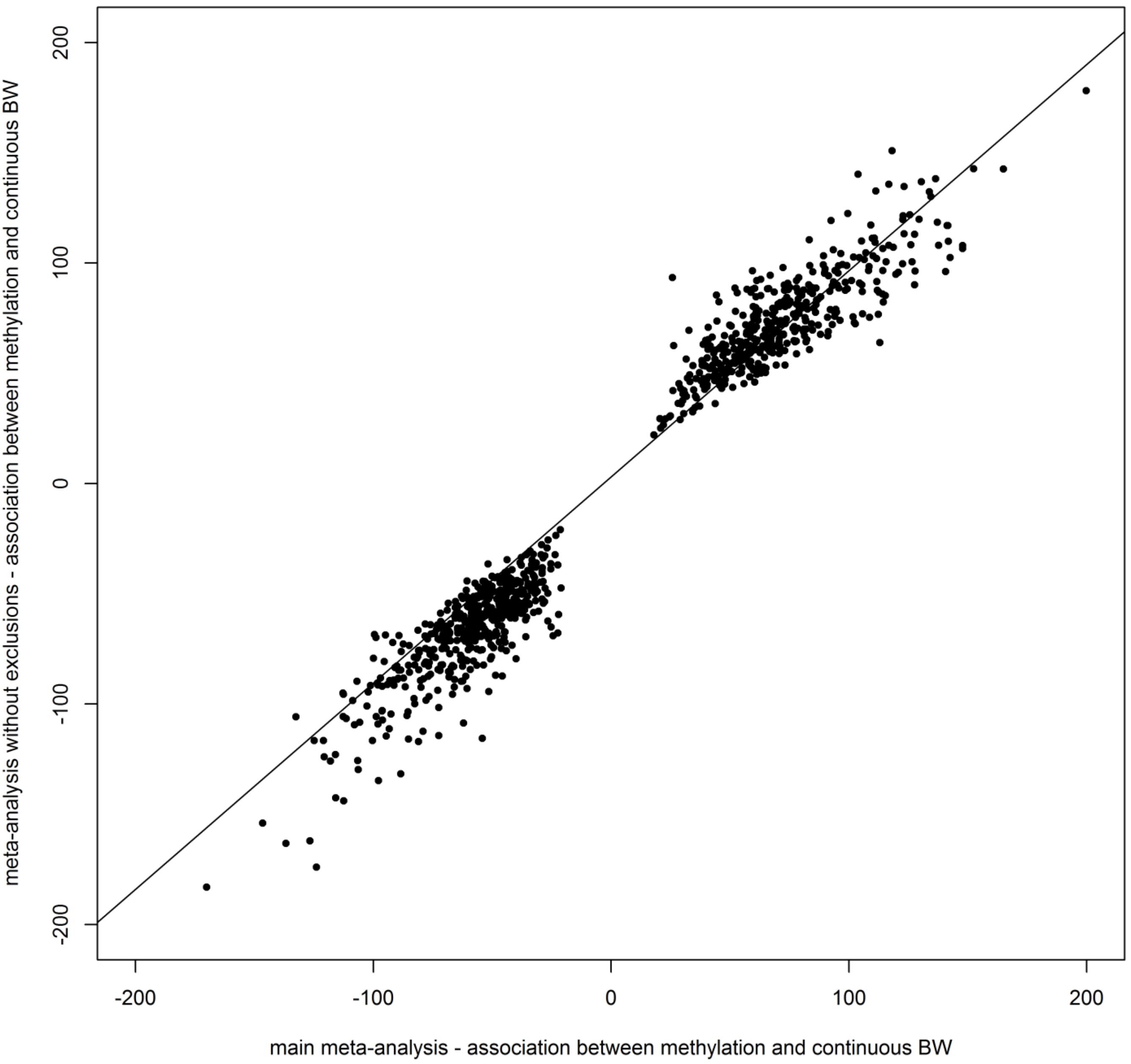

Supplementary Figure 4. Correlation of the point estimates from the meta-analysis including preterms, pre-eclampsia and diabetes versus main meta-analysis excluding these participants (x-axis)

Correlation of the point estimates for a secondary meta-analysis of the main association of DNA methylation with birthweight including infants born preterm, or to mothers with pre-eclampsia or diabetes (5,414 participants from 9 studies, y-axis) with point estimates for the main meta-analysis with 8,809 participants from 24 studies of mixed ancestry (x-axis).

SUPPLEMENTARY NOTE

Supplementary Funding: study specific and in alphabetical order

ALSPAC

The UK Medical Research Council and the Wellcome Trust (Grant ref: 102215/2/13/2) and the University of Bristol provide core support for ALSPAC. The Accessible Resource for Integrated Epigenomics Studies (ARIES) which generated large scale methylation data was funded by the UK Biotechnology and Biological Sciences Research Council (BB/I025751/1 and BB/I025263/1). Additional epigenetic profiling on the ALSPAC cohort and the contributions of LKK, DAL, CLR, were supported by the UK Medical Research Council Integrative Epidemiology Unit and the University of Bristol (MC_UU_12013_1, MC_UU_12013_2, MC_UU_12013_5 and MC_UU_12013_8). Contributions of authors related to this study and/or additional ALSPAC data were also supported by the Wellcome Trust (WT088806), the United States National Institute of Diabetes and Digestive and Kidney Diseases (R01 DK10324), and the European Research Council under the European Union's Seventh Framework Programme (FP/2007-2013) / ERC Grant Agreement (Grant number 669545; DevelopObese). The funders had no role in study design, data collection and analysis, decision to publish, or preparation of the manuscript. As well as funds from national government and charities, DAL declares that she has received industry support from Roche Diagnostics and Medtronic for work unrelated to that presented here. This project received funding from the European Union's Horizon 2020 research and innovation programme (733206, LIFECYCLE).

BAMSE

BAMSE was supported by The Swedish Research Council, The Swedish Heart-Lung Foundation, MeDALL (Mechanisms of the Development of ALLergy) a collaborative project conducted within the European Union (grant agreement No. 261357), Stockholm County Council (ALF), Swedish foundation for strategic research (SSF) (RBc08-0027), the Strategic Research Programme (SFO) in Epidemiology at Karolinska Institutet and the Swedish Research Council Formas. EM is supported by grants from the Swedish Research Council and the Strategic Research Area Epidemiology at Karolinska Institutet.

CBC

This work was supported by the National Institute of Environmental Health Sciences and the Environmental Protection Agency (grant number: P01ES018172 to C.M., R.R. and J.L.W.); The National Institute of Environmental Health Sciences (grant number: R01ES09137 to C.M., R.R. and J.L.W.); The National Cancer Institute (grant number: R01CA155461 to R.R. and J.L.W.); The Swiss Cancer League (grant number: BIL KLS 3124–02–2013 to S. G.); The Swiss Science National Foundation (grants numbers: P2LAP3_158674 to S.G., P2LAP3_148434 to S.N.); The SICPA Foundation to S.G.; the Sutter-Stottner Foundation to S.G.; a Cancer Center Support Grant (5P30CA082103) to R.R. The content is solely the responsibility of the authors and does not necessarily represent the official views of the National Institutes of Health or the Environmental Protection Agency.

CHAMACOS

CHAMACOS cohort research was supported by grants from the National Institute of Environmental Health Science (NIEHS) [P01 ESO09605, 5UG30D023356, R01ES012503, R01ES021369, R01ES023067, R24ES0285529]; Environmental Protection Agency (RD83273401, RD83171001), and the JPB Foundation. Its contents are solely the responsibility of the authors and do not necessarily represent the official views of NIEHS, EPA, or JPB Foundation.

CHS

This work was supported by NIEHS grants K01ES017801, R01ES022216, and P30ES007048.

EARLI

Funding for the EARLI study was provided by the National Institutes of Health (R01ES016443, PI: Newschaffer) and Autism Speaks. The DNA methylation measures in this study were supported by the National Institutes of Health (R01 ES017646, MPI: Fallin, Feinberg). KB was supported by NIH grants R01ES025531, R01ES025574, and R01 AG055406.

EXPOsOMICS

Piccolipiu: The study was approved and initially funded by the Italian National Centre for Disease Prevention and Control (CCM grant 2010) and by the Italian Ministry of Health (art 12 and 12bis Dl.gs.vo 502/92). The methylation assays were funded by the European Community's Seventh Framework Programme FP7/2007–2013 project EXPOsOMICS (grant no. 308610)

Rhea: The methylation assays were funded by the European Community's Seventh Framework Programme FP7/2007-2013 project EXPOsOMICS (grant no. 308610).

ENVIRonAGE: The ENVIRonAGE birth cohort is funded by the European Research Counsil (ERC-2012-StG.310898) and by funds of the Flemisch Scientific Research Council (FWO, N1516112 / G.0.873.11N.10). The methylation assays were funded by the European Community's Seventh Framework Programme FP7/2007-2013 project EXPOsOMICS (grant no. 308610).

ZH and the Epigenetics Group at IARC is supported by the Exposomics EC FP7 grant (Grant agreement no: 308610), INCa/Plan Cancer-EVA-INSERM (France), two grants from the Bill and Melinda Gates Foundation (Grand Challenges Exploration Grant - OPP1061062, and Global Health Grant - OPP1066947) and Association pour la Recherche sur le Cancer (ARC, France). AG was supported by the grant from INCa/IPlan Cancer-EVA-INSERM (France) and by the IARC Postdoctoral Fellowship, partially supported by the EC FP7 Marie Curie Actions-People-Co-funding of regional, national and international programmes.

FLEHS1

We thank FLEHS Supervisory Board for the provision of data. The FLEHS studies were commissioned, financed and steered by the Flemish Government (Department of Economy, Science and Innovations, Agency for Care and Health and Department of Environment). The methylation work was funded by the CEFIC LRI award 2013 that was given to Dr Sabine Langie. SASL is the beneficiary of a post-doctoral fellowship [12L5216N; http://www.fwo.be/] provided by The Research Foundation-Flanders (FWO) and the Flemish Institute for Technological Research (VITO).

GECKO

The GECKO Drenthe birth cohort was funded by an unrestricted grant of Hutchison Whampoa Ld, Hong Kong and supported by the University of Groningen, Well Baby Clinic Foundation Icare, Noordlease and Youth Health Care Drenthe. This methylation project in the GECKO Drenthe cohort was supported by the Biobanking and Biomolecular Research Infrastructure Netherlands (CP2011-19). This project received funding from the European Union's Horizon 2020 research and innovation programme (733206, LIFECYCLE).

Gen3G

This work was supported by a Fonds de recherche du Québec en santé (FRQ-S) operating grant (to M-FH, grant #20697); a Canadian Institute of Health Reseach (CIHR) Operating grant (to M-FH grant #MOP 115071); a Diabète Québec grant (to PP) and a Canadian Diabetes Association operating grant (to J-LA, grant #OG-3-08-2622-JA). LB is a junior scholar from Fonds de recherche du Québec en santé (FRQ-S) and a member of the FRQS-funded Centre de recherche du CHUS (affiliated to the Centre hospitalier universitaire de Sherbrooke). MFH was a FRQ-S Junior 1 research scholar and received a Clinical Scientist Award by the Canadian Diabetes Association and the Maud Menten Award from the CIHR-Institute of Genetics, and an American Diabetes Association Pathways Accelerator Early Investigator Award (No 1-15-ACE-26).

Generation R

The general design of the Generation R Study is made possible by financial support from the Erasmus Medical Center, Rotterdam, the Erasmus University Rotterdam, the Netherlands Organization for Health Research and Development and the Ministry of Health, Welfare and Sport. The EWAS data was funded by a grant to VWJ from the Netherlands Genomics Initiative (NGI)/Netherlands Organisation for Scientific Research (NWO) Netherlands Consortium for Healthy Aging (NCHA; project nr. 050-060-810), by funds from the Genetic Laboratory of the Department of Internal Medicine, Erasmus MC, and by a grant from the National Institute of Child and Human Development (R01HD068437). VWJ received an additional grant from the Netherlands Organization for Health Research and Development (VIDI 016.136.361) and a Consolidator Grant from the European Research Council (ERC-2014-CoG-648916). JFF has received funding from the European Union's Horizon 2020 research and innovation programme under grant agreement No 633595 (DynaHEALTH) and from the European Joint Programming Initiative "A Healthy Diet for a Healthy Life" (JPI HDHL, NutriPROGRAM project, ZonMw the Netherlands no.529051022). This project received funding from the European Union's Horizon 2020 research and innovation programme (733206, LIFECYCLE). LD received funding from the co-funded programme ERA-Net on Biomarkers for Nutrition and Health (ERA HDHL) (ALPHABET project, Horizon 2020 (grant agreement no 696295; 2017), ZonMW The Netherlands (no 529051014; 2017)).

Glaku

The Glaku cohort has been supported by the Academy of Finland, the Signe and Ane Gyllenberg Foundation, the National Doctoral Programme of Psychology, the Finnish Ministry of Education and Culture, the Emil Aaltonen Foundation, the Foundation for Pediatric Research, the Foundation for Cardiovascular Research, the Juho Vainio Foundation, the Novo Nordisk Foundation, the Sigrid Jusélius Foundation, the Yrjö Jahnsson Foundation, and the University of Helsinki Research Funds. Epigenetic analyses were funded by the Hope and Optimism Initiative.

GOYA

The Danish National Birth Cohort was established with a significant grant from the Danish National Research Foundation. Additional support was obtained from the Danish Regional Committees, the Pharmacy Foundation, the Egmont Foundation, the March of Dimes Birth Defects Foundation, the Health Foundation and other minor grants. The DNBC Biobank has been supported by the Novo Nordisk Foundation and the Lundbeck Foundation. Generation of DNA methylation data was funded by the MRC Integrative Epidemiology Unit which is supported by the Medical Research Council (MC_UU_12013/1-9) and the University of Bristol.

Healthy Start

The Healthy Start study is funded by grants from the National Institute of Diabetes and Digestive and Kidney diseases (R01DK076648) and the National Institutes of Health, Office of the Director, Environmental Influences on Child Health Outcomes (ECHO) Program (1UG3OD023248). The DNA methylation analysis in Healthy Start was funded by a grant from the National Institute of Environmental Health Sciences (R01ES022934). APS was funded by a grant from the National Institute of Environmental Health Sciences (K99ES025817).

INMA

This study was funded by grants from Instituto de Salud Carlos III (Red INMA G03/176), Generalitat de Catalunya-CIRIT 1999SGR 00241, and EU Commission (261357). This project received funding from the European Union's Horizon 2020 research and innovation programme (733206, LIFECYCLE).

IOW F1

The IoW 1989 (IOW F2) cohort was supported by the National Institute of Allergy and Infectious Diseases under award number R01 AI091905 (PI: Wilfried Karmaus) and R01 AI061471 (PI: Susan Ewart). The 18-year follow-up by a grant from the National Heart and Blood Institute (R01 HL082925, PI: S. Hasan Arshad).

IOW F2

The third generation study was funded by the National Institute of Allergy and Infectious Diseases (NIAID) at the National Institute of Health, R01 AI091905 (PI: Wilfried Karmaus). The work of Hongmei Zhang and John Holloway is also supported by the fund from NIAID/NIH (R01AI121226, MPI: Hongmei Zhang and John Holloway).

Lifelines

This study is sponsored by grant 4.1.13.007 from the Lung Foundation (Longfonds), the Netherlands. D.A.v.d.P. is supported by grant 4.1.13.007 from Longfonds. The LifeLines cohort study was supported by the Dutch Ministry of Health, Welfare, and Sport; the Ministry of Economic Affairs, Agriculture, and Innovation; the Province of Groningen; the European Union (regional development fund); the Northern Netherlands Provinces; the Netherlands Organization for Scientific Research; University Medical Center Groningen; University of Groningen, de Nierstichting (the Dutch Kidney Foundation); and the Diabetes Fonds (the Diabetic Foundation). The LifeLinesBiobankinitiative has been made possible by funds from FES (Fonds Economische Structuurversterking), SNN (Samenwerkingsverband Noord Nederland) and REP (Ruimtelijke Economisch Programma). The methylation study is funded by grant number 4.1.13.007 of Lung Foundation Netherlands (Longfonds). DAvdP is supported by grant number 4.113.007 of Longfonds.

MoBa1, MoBa2 and MoBa3

The Norwegian Mother and Child Cohort Study are supported by the Norwegian Ministry of Health and Care Services and the Ministry of Education and Research, NIH/NIEHS (contract no N01-ES-75558), NIH/NINDS (grant no.1 UO1 NS 047537-01 and grant no.2 UO1 NS 047537-06A1). For this work, MoBa 1 and 2 were supported by the Intramural Research Program of the NIH, National Institute of Environmental Health Sciences (Z01-ES-49019) and the Norwegian Research Council/BIOBANK (grant no 221097). This work was partly supported by the Research Council of Norway through its Centres of Excellence funding scheme, project number 262700. The work in MoBa3 was supported in part by a Postdoctoral Fellowship grant from the Ullevål Hospitals Research Council (now under Oslo University Hospital) and travel grants from the Unger -Vetlesens foundation and the Norwegian American Womens Club, all to MCMK. MoBa3 methylation sample retrieval was funded by INCA/INSERM-Plan Cancer, France, and the International

Childhood Cancer Cohort Consortium (I4C). The work performed by the Epigenetics Group at the International Agency for Research on Cancer (IARC, Lyon, France), ZH and AG were supported by the grant from Institut National du Cancer (INCa) / INSERM-Plan Cancer (France, 2015), and Association pour la Recherche sur le Cancer (ARC, France). AG was also supported by the IARC Postdoctoral Fellowship, partially supported by the EC FP7 Marie Curie Actions-People-Co-funding of regional, national and international programmes (COFUND). This project received funding from the European Union's Horizon 2020 research and innovation programme (733206, LIFECYCLE).

NCL
This work was supported by Intramural Research Program of the NIH, National Institute of Environmental Health Sciences (Z01 ES049033, and Z01 ES049032).

NEST
The NEST study was funded by NIEHS grants R21ES014947 and R01ES016772 and NIDDK grant R01DK085173. CH and DDJ have received funding from the National Institute of Environmental Health Science (P30 ES025128).

NFBC1966 and NFBC1986
NFBC1966 received financial support from University of Oulu Grant no. 65354, Oulu University Hospital Grant no. 2/97, 8/97, Ministry of Health and Social Affairs Grant no. 23/251/97, 160/97, 190/97, National Institute for Health and Welfare, Helsinki Grant no. 54121, Regional Institute of Occupational Health, Oulu, Finland Grant no. 50621, 54231. NFBC1986 received financial support from EU QLG1-CT-2000-01643 (EUROBLCS) Grant no. E51560, NorFA Grant no. 731, 20056, 30167, USA / NIHH 2000 G DF682 Grant no. 50945. MW was supported by the European Union's Horizon 2020 research and innovation programme under grant agreement No 633212. MRJ and SS are supported by H2020-633595 DynaHEALTH action and academy of Finland EGEA-project (285547). This project received funding from the European Union's Horizon 2020 research and innovation programme (733206, LIFECYCLE).

NHBCS
Funding for the New Hampshire Birth Cohort Study was provided in part by National Institute of Environmental Health Sciences at NIH, USA, awards P42ES007373, P01ES022832, the National Institute of General Medical Sciences award P20GM104416, and by Environmental Protection Agency, USA, under grant number RD83544201. The contents are solely the responsibility of the grantee and do not necessarily represent the official views of the US EPA and the US EPA does not endorse the purchase of any commercial products or services mentioned in the presentation.

NTR
We would like to thank the twin families for their participation and funding agencies for their support, including the Netherlands Organization for Scientific Research(NWO900-562-137, 904-61-090, 985-10-002, 904-61-193, 56-464-14192, 400-03-330, 480-04-004, 400-07-080, 911-09-032, 451-06-004, 451-08-026, 451-10-005),the BBRMI-NL - BIOS Consortium (NWO184.021.007), the Netherlands Organization for Health Research and Development (ZonMW 3100.0038, 940-37-024, 31160008), National Institutes of Health (NIH 5R37DA018673–03, R01 MH059160, 1RC2 MH089951-01, 4R37DA018673-06, 1R01 MH087646-01A1), European Research Council (230374-GMI, 284167), Rutgers University (3797), and the Avera Institute for Human Genetics. We gratefully acknowledge grant NWO 480-15-001/674: Netherlands Twin Registry Repository: researching the interplay between genome and environment. JvD is supported by BBRMI-NL (NWO 184.021.007) and ACTION. ACTION receives funding from the European Union Seventh Framework Program (FP7/2007-2013) under grant agreement no 602768. DIB acknowledges the Academy Professor Award (PAH/6635).

PIAMA
The PIAMA study is supported by The Netherlands Organization for Health Research and Development; The Netherlands Organization for Scientific Research; The Lung Foundation of the Netherlands (grant number AF 45.1.14.001 supported the methylation assays); The Netherlands Ministry of Spatial Planning, Housing, and the Environment; and The Netherlands Ministry of Health, Welfare, and Sport.

PREDO
The Academy of Finland, EraNet, EVO (a special state subsidy for health science research), University of Helsinki Research Funds, the Signe and Ane Gyllenberg foundation, the Emil Aaltonen Foundation, the Finnish Medical Foundation, the Jane and Aatos Erkko Foundation, the Novo Nordisk Foundation, the Päivikki and Sakari Sohlberg Foundation, the Sigrid Juselius Foundation. KR acknowledges funding by the Academy of Finland (284859, 2848591, 312670); European Commission (Horizon 2020 Award SC1-2016-RTD-733280 RECAP); Signe and Ane Gyllenberg Foundation. This project received funding from the European Union's Horizon 2020 research and innovation programme (733206, LIFECYCLE).

PRISM
This research was supported by NIH grants R01 HL095606, R01 HL114396, P30 ES023515, R01 ES021357 and R01 NR013945. ACJ was supported by grant R00 ES023450.

PROGRESS
The PROGRESS project has been funded by grants R01 ES021357 and R01 ES013744 (R.O. Wright, principal investigator). Phenotyping and biostatistical support was funded by grant P30 ES023515.

Project Viva
This study was supported by grants from the National Institutes of Health (R01 NR013945, R01 HL111108, R01 HD 034568, K24 HD069408, K23 ES022242). SL was supported by NIGMS grant T32 GM74905

Raine
The authors acknowledge the contributions to core funding of the Raine Study by the University of Western Australia, the Telethon Kids Institute, the Raine Medical Research Foundation, the Faculty of Medicine, Dentistry and Health Science (UWA), the Women and Infants Research Foundation, Curtin University, and Edith Cowan University. This work was supported by resources provided by the Pawsey Supercomputing Centre with funding from the Australian Government and Government of Western Australia. The authors also acknowledge the long-term support of the National Health and Medical Research Council of Australia. The epigenetic data collection is supported by NHMRC grant #1059711. RCH supported by a National Health Medical Research Fund Fellowship 1053384. This project received funding from the European Union’s Horizon 2020 research and innovation programme (733206, LIFECYCLE).

RICHS
This work was supported by the National Institutes of Health [NIH-NIMH R01MH094609, NIH-NIEHS R01ES022223, NIH-NIEHS P01ES022832, NIH-NIEHS R24ES028507] and by the United States Environmental Protection Agency [US EPA grant RD83544201]. Its contents are solely the responsibility of the grantee and do not necessarily represent the official views of the US EPA. Further, the US EPA does not endorse the purchase of any commercial products or services mentioned in the presentation.

STOPPA
Financial support was provided by the Swedish Research Council through the Swedish Initiative for research on Microdata in the Social And Medical Sciences (SIMSAM) framework grant number 340-2013-5867, grants provided by the Stockholm County Council (ALF projects), the Swedish Heart Lung Foundation, the Swedish Asthma and Allergy Association's Research Foundation, FORTE and Stiftelsen Frimurare Barnahuset Stockholm. CA acknowledges support by the Swedish Research Council (SIMSAM), grants provided by the Stockholm County Council (ALF projects), the Swedish Heart Lung Foundation, the Swedish Asthma and Allergy Association's Research Foundation, FORTE and Stiftelsen Frimurare Barnahuset Stockholm. We acknowledge the Swedish Twin Registry for access to data. The Swedish Twin Registry is managed by Karolinska Institutet and receives funding through the Swedish Research Council under the grant no 2017-00641.

The Gambia
The ENID Trial was jointly funded by the MRC & the Department for International Development (DFID) under the MRC/DFID Concordat agreement (MRC Programme MC-A760-5QX00). The funders had no role in study design, data collection and analysis, decision to publish, or preparation of the manuscript. The aflatoxin sub-study and the methylation analysis were supported by the Bill and Melinda Gates Foundation. YYG received financial support via BMGF OPP1066947.

Supplementary Acknowledgements: study specific and in alphabetical order

ALSPAC

We are extremely grateful to all the families who took part in this study, the midwives for their help in recruiting them, and the whole ALSPAC team, which includes interviewers, computer and laboratory technicians, clerical workers, research scientists, volunteers, managers, receptionists, and nurses. We would like to acknowledge Tom Gaunt, Oliver Lyttleton, Sue Ring, Nabila Kazmi, and Geoff Woodward for their earlier contribution to the generation of ARIES data (ALSPAC methylation data).

BAMSE

We would like to thank all participating BAMSE children and parents for the engagement in our study.

CBC

We are grateful for the efforts of Marty Kharrazi, Robin Cooley, and Steve Graham from the California Department of Health Services, Genetic Diseases Branch.

CHAMACOS

We are grateful to the laboratory and field staff and participants of the CHAMACOS study for their contributions. We are thankful to Hong Quach who helped with 450K methylation analyses.

CHS

We are indebted to the school principals, teachers, students and parents in each of the study communities for their cooperation and especially to the members of the health testing field team for their efforts. We would like to express our sincere gratitude to Steve Graham and Robin Cooley at the California Biobank Program and Genetic Disease Screening Program within the California Department of Public Health for their assistance and advice regarding newborn bloodspots. The biospecimens and/or data used in this study were obtained from the California Biobank Program, (SIS request number(s) 479)" Section 6555(b), 17 CCR. The California Department of Public Health is not responsible for the results or conclusions drawn by the authors of this publication.

EARLI

We thank the EARLI study participants and staff. We thank the Johns Hopkins Biological Repository (JHBR) for archiving and processing the samples. We thank the JHU SNP Center for conducting the DNA methylation measurements.

EXPOsOMICS

Piccolipiu: Our thanks go to all the families who took part in this study, to the midwives for their help in recruiting them, and to the whole Piccolipiu team, which includes doctors, nurses, research scientists and computer/laboratory technicians. The authors acknowledge Paolo Vineis for his input and the coordination of EXPOsOMICS.

Rhea: We thank all of the participants of the Rhea study and the interviewers, statisticians and the hospital personnel for their cooperation and contribution to this study. The authors acknowledge Paolo Vineis for his input and the coordination of EXPOsOMICS.

ENVIRonAGE: The authors acknowledge the participating mothers and neonates, as well as the staff of the maternity ward, midwives and the staff of the clinical laboratory of East-Limburg Hospital in Genk. The authors acknowledge Paolo Vineis for his input and the coordination of EXPOsOMICS.

FLEHS1

The authors thank all the children and their parents for their cooperation, and all the field workers and laboratory personnel involved for their efforts. The authors also thank the groups of Guy Van Camp and Wim Vanden Berghe, University of Antwerp, for their support in the methylation analysis, and the group of Diether Lambrechts at the VIB-KU Leuven Center for Cancer Biology for the bioinformatics analysis.

GECKO

We are grateful to the families who took part in the GECKO Drenthe birth cohort, the midwives, gyneacologists, nurses and GPs for their help for recruitment and measurement of participants, and the whole team from the GECKO Drenthe study.

Gen3G

The authors acknowledge the blood sampling in pregnancy clinic at the CHUS; the assistance of clinical research nurses (M Gérard, G Proulx, S Hayes, and M-J Gosselin) and research assistants (C Rousseau and P Brassard) for recruiting

women and obtaining consent for the study; and the CHUS biomedical laboratory for performing assays. They also thank the Research in obstetrics services, led by collaborator Dr J-C Pasquier for their help in collecting samples at delivery. We also thank our diligent research staff (M Doyon, MC Battista) for recruitment, follow-up, and for running all operating procedures necessary for the initiation and maintenance of Gen3G.

Generation R
The Generation R Study is conducted by the Erasmus Medical Center in close collaboration with the School of Law and Faculty of Social Sciences of the Erasmus University Rotterdam, the Municipal Health Service Rotterdam area, Rotterdam, the Rotterdam Homecare Foundation, Rotterdam and the Stichting Trombosedienst & Artsenlaboratorium Rijnmond (STAR-MDC), Rotterdam. We gratefully acknowledge the contribution of children and parents, general practitioners, hospitals, midwives and pharmacies in Rotterdam. The study protocol was approved by the Medical Ethical Committee of the Erasmus Medical Centre, Rotterdam. Written informed consent was obtained for all participants. The generation and management of the Illumina 450K methylation array data (EWAS data) for the Generation R Study was executed by the Human Genotyping Facility of the Genetic Laboratory of the Department of Internal Medicine, Erasmus MC, the Netherlands. We thank Mr. Michael Verbiest, Ms. Mila Jhamai, Ms. Sarah Higgins, Mr. Marijn Verkerk and Dr. Lisette Stolk for their help in creating the EWAS database. We thank Dr. A.Teumer for his work on the quality control and normalization scripts.

Glaku
We are grateful to the midwives, gynaecologists, nurses, and the study team involved in the Glaku study.

GOYA
The authors want to thank the many pregnant women who have taken part in the study. Without them, there would be no cohort.

Healthy Start
We would like to acknowledge the contributions of the study coordinator, Ms. Mercedes Martinez, MPH, the study staff, and the participating families for their continued involvement in the Healthy Start study.

INMA
The authors would particularly like to thank all the participants for their generous collaboration. A full roster of the INMA Project Investigators can be found at http://www.proyectoinma.org/presentacion-inma/listado-investigadores/en_listado-investigadores.html.

IOW F1
We would like to thank all the participants of the Isle of Wight birth cohort, the research team at David Hide Asthma & Allergy Research Centre (Isle of Wight) for collecting the data, Nikki Graham for technical support and other members of the IoW research group for valuable discussion. DNA methylation data was generated by the Oxford Genomics Centre at the Wellcome Trust Centre for Human Genetics.

IOW F2
We are sincerely thankful to all the families who took part in this study, the nurses for their help in recruiting them, and the whole IOW team. In particular, we would like to thank Stephen Porter, Sharon Matthews, and Frances Mitchell.

Lifelines
We wish to acknowledge the services of the LifeLines Cohort Study, the contributing research centers delivering data to LifeLines and the participating general practitioners, and all the study participants.

MoBa1, MoBa2 and MoBa3
We are grateful to all the participating families in Norway who take part in this on-going cohort study, and to the International Childhood Cancer Cohort Consortium (I4C), which MoBa3 is part of.

NCL
We would like to thank all individuals for participating in the Norway Facial Clefts Study, Norwegian Mother and Child Cohort Study. We are grateful to all the participating families in Norway who take part in this ongoing cohort study.

NEST
We thank the parents and other caregivers of the Newborn Epigenetics STudy. We also thank the field and laboratory staff for their effort.

NFBC1966 and NFBC1986
We thank Professor Paula Rantakallio (launch of NFBC1966 and initial data collection). We gratefully acknowledge the contributions of the participants in the Northern Finland Birth Cohort 1966 study and the Northern Finland Birth Cohort 1986. We also thank all the field workers and laboratory personnel for their efforts.

NHBCS
We are grateful to the research staff involved in data collection and thank all of the families that participated in the New Hampshire Birth Cohort Study.

NTR
We would like to thank the twins and their family members who participated in the NTR studies.

PIAMA
We thank all the children and their parents for their cooperation. We also thank all the field workers and laboratory personnel involved for their efforts, and Marjan Tewis for data management.

PREDO
The PREDO study would not have been possible without the dedicated contribution of the PREDO Study group members: A Aitokallio-Tallberg, A-M Henry, VK Hiilesmaa, T Karipohja, R Meri, S Sainio, T Saisto, S Suomalainen-König, V-M Ulander, T Vaitilo (Department of Obstetrics and Gynaecology, University of Helsinki and Helsinki University Central Hospital, Helsinki, Finland), L Keski-Nisula (Kuopio University Hospital, Kuopio Finland), E Koistinen, T Walle, R Solja, P Taipale (Northern Karelia Central Hospital, Joensuu, Finland), M Kurkinen (Päijät-Häme Central Hospital, Lahti, Finland), P Staven (Iisalmi Hospital, Iisalmi, Finland), J Uotila (Tampere University Hospital, Tampere, Finland). We also thank the PREDO cohort mothers, fathers and children for their enthusiastic participation.

PRISM
We are grateful for the ongoing participation of PRISM families.

PROGRESS
We thank the American British Cowdray (ABC) Hospital in Mexico City for providing research and valuable assistance in the data collection.

Project Viva
We are grateful to families who participate in Project Viva.

Raine
The authors are grateful to the Raine Study participants and their families, and the Raine Study management team for cohort co-ordination and data collection.

RICHS
We are grateful to the research staff involved in data collection and thank all of the families that participated in the Rhode Island Child Health Study.

STOPPA
First, we direct our greatest appreciation to the twins and parents of the STOPPA cohort, without whose participation this study could not have been performed. We are also indebted to the STOPPA research nurses and database managers for their excellent data collection and data managing. We also want to direct our thanks to the eight paediatric allergy clinics around Sweden for their great collaboration during our visits for clinical examinations.

The Gambia
We thank the women of West Kiang who patiently participated in the ENID study. We acknowledge the enthusiastic work of the ENID study team, especially the fieldworkers, village assistants, midwives, clinical staff and laboratory technicians who tirelessly collected the data and samples. We acknowledge the support of Mr Bakary Sonko and colleagues in the Keneba data office, for the development and management of the ENID database.

Supplementary Methods: study specific and in alphabetical order

Avon Longitudinal Study of Parents and Children (ALSPAC)
Design and study population
ALSPAC is a large, prospective cohort study based in the South West of England. 14,541 pregnant women resident in the previous county of Avon (centred around the city of Bristol), UK with expected dates of delivery 1st April 1991 to 31st December 1992 were recruited. Detailed information has been collected on these women, their partners and their offspring at regular intervals to the present date*(1, 2)*. The study website contains details of all the data that is available through a fully searchable data dictionary (http://www.bris.ac.uk/alspac/researchers/data-access/data-dictionary/).
Written informed consent has been obtained for all ALSPAC participants. Ethical approval for the study was obtained from the ALSPAC Ethics and Law Committee and the Local Research Ethics Committees.

Birthweight
Information on birthweight was recorded by healthcare professionals at the time of birth and extracted from birth records. Within ALSPAC, there were no values +/- 5SD from the mean.

Methylation measurements
Cord blood was collected according to standard procedures, spun and frozen at -80˚C. DNA methylation analysis and data pre-processing were performed at the University of Bristol as part of the ARIES project (ariesepigenomics.org.uk)*(3)*. Following extraction, DNA was bisulfite converted using the Zymo EZ DNA MethylationTM kit (Zymo, Irvine, CA). Following conversion, the genome-wide methylation status of over 485,000 CpGs was measured using the Illumina Infinium® HumanMethylation450k BeadChip assay according to the standard protocol. The arrays were scanned using an Illumina iScan and initial quality review was assessed using GenomeStudio (version 2011.1). The level of methylation is expressed as a "Beta" value (β-value), ranging from 0 (no cytosine methylation) to 1 (complete cytosine methylation). Samples from all time-points in ARIES were distributed across slides using a semi-random approach (sampling criteria were in place to ensure that all time-points were represented on each array) to minimize the possibility of confounding by batch effects. Samples failing quality control (average probe detection p-value ≥ 0.01) were repeated. As an additional quality control step genotype probes on the HumanMethylation450k were compared between samples from the same individual and against SNP-chip data to identify and remove any sample mismatches. Data were pre-processed in R (version 3.0.1) with the WateRmelon package according to the subset quantile normalization approach described by Touleimat & Tost in an attempt to reduce the non-biological differences between probes.
We removed probes that had a detection P-value >0.05 for >5% of samples (3034 probes), probes on the X or Y chromosomes and SNPs (rs probes). 471192 probes remained.

Covariates
Data on maternal parity, socio-economic status, smoking, pre-pregnancy body mass index and maternal age at delivery were self-reported by questionnaire during pregnancy. Newborn sex was extracted from birth records and questionnaires after birth. Gestational age at delivery was also extracted from birth records. Obstetric practice and antenatal care at the time means that for most participants gestational age will have been estimated based on the last menstrual period (LMP), supplemented by ultrasound scans and paediatric/obstetric assessment of the newborn at birth. Information on pre-eclampsia and diabetes were extracted from hospital records. For pre-eclampsia all records of any blood pressure and proteinuria across the entire pregnancy were extracted and the International Society for the Study of Hypertension in Pregnancy (ISSHP) criteria applied (Systolic blood pressure >139mmHg OR diastolic blood pressure >89mmHg on at least 2 occasions after 20 weeks of gestation in women who had not previously been diagnosed with hypertension outside of pregnancy, together with proteinuria of 30mg/dl occurring at the same time as the two occasions of elevated blood pressure)*(4)*.

Cell type correction
We used the Reinius-based Houseman method*(5, 6)* with the *estimateCellCounts* function in the Minfi package*(7)* in R*(8)* to estimate relative proportions of six white blood cell subtypes (CD4+ T-lymphocytes, CD8+ T-lymphocytes, NK (natural killer) cells, B-lymphocytes, monocytes and granulocytes).

Batch correction
Ten surrogate variables were generated using the sva package in R and included in models to adjust for technical batch.

Exclusion criteria
For the main analysis we excluded n=38 participants: 19 infants born preterm (<37 weeks), and whose mothers experienced pre-eclampsia (n=12) or diabetes (n=3) during pregnancy, or participants with missing data on either of these. This information was obtained from questionnaires completed by the mothers shortly after delivery and combined with medical birth records.

Children (Barn), Allergy, Milieu, Stockholm, Epidemiology (BAMSE)
Design and study population

BAMSE (Children, Allergy, Milieu, Stockholm, Epidemiology in Swedish) is a prospective population-based cohort study of children recruited at birth and followed during childhood and adolescence. Details of the study design, inclusion criteria, enrolment and data collection are described elsewhere*(9)*. In short, 4,089 children born between 1994 and 1996 in four municipalities of Stockholm County were enrolled. At baseline, when the infant was approximately 2 months of age, parents completed a questionnaire that assessed residential characteristics, as well as socioeconomic and lifestyle factors. When children were 1, 2, 4, 8, 12 and 16 years, the parents completed questionnaires focusing on children's symptoms related to wheezing and allergic diseases, as well as various exposures. The survey response rates were 96%, 94%, 91%, 84%, 82% and 78%, respectively*(10)*. Furthermore, blood was obtained at ages 4, 8 and 16 years from 2,605 (63.7%), 2,470 (60.4%) and 2,547 (62.2%) children, respectively. The baseline and follow-up studies were approved by the Regional Ethical Review Board, Karolinska Institutet, Stockholm, Sweden, and the parents of all participating children provided written informed consent.

Birthweight

Birthweight was assessed by questionnaire at the time of recruitment (at a median age of the children of 2 months), and used as a continuous variable in the analyses*(11)*.

Methylation measurements

For this methylation study, we used data from the 8- and 16-year follow-up. At 8 years, epigenome-wide DNA methylation was measured in 472 Caucasian children and at 16 years, methylation was measured in 269 Caucasian children*(12)*. 500 ng DNA per sample underwent bisulfite conversion using the EZ-96 DNA Methylation kit (Zymo Research Corporation, Irvine, USA). Samples were plated onto 96-well plates in randomized order. Samples were processed with the Illumina Infinium HumanMethylation450 BeadChip (Illumina Inc., San Diego, USA). Quality control of analyzed samples was performed using standardized criteria. Samples were excluded in case of sample call rate <99%, colour balance >3, low staining efficiency, poor extension efficiency, poor hybridization performance, low stripping efficiency after extension and poor bisulfite conversion. We also applied multidimensional scaling (MDS) plot to evaluate gender outliers based on chromosome X data, that produced two separated clusters for male and female. Samples that did not belong to the distinct cluster were removed. Furthermore, we applied median intensity plot for methylated and unmethylated intensity by using the minfi R package (samples below the 10.5 cutoff were excluded). Applying these criteria resulted in exclusion of 8 (8-year DNA) and 2 (16-year DNA) samples, respectively. Probes with a single nucleotide polymorphism in the single base extension site with a frequency of >5% were excluded*(13)*, as were probes with non-optimal binding (non-mapping or mapping multiple times to either the normal or the bisulphite-converted genome), and the probed belonging to chr X and chr Y, resulting in the exclusion of 46,799 and 47,654 probes (8- and 16-year DNA), leaving a total of 438,713 and 437,858 probes, respectively, in the analyses. Furthermore, we implemented "DASEN" recommended from wateRmelon package to do signal correction and normalization*(14)*.

Covariates

Information on maternal BMI in the first trimester and parity was collected from the Birth Registry and parity was categorized into 0 and ≥1*(10)*. Maternal age, sex, maternal smoking status and maternal socio-economic status information were collected from a questionnaire administered at enrolment. Maternal socio economic was categorized into blue collar worker, white collar worker and other students, unemployed, housewives. Gestational age was from both the Birth Registry and a questionnaire administered at enrolment. Current age and current BMI was collected from the 8- and 16-year questionnaires, respectively. Ever doctor's diagnosis of asthma was considered to be a selection factor in the 8-year data, but not at 16 years.

Cell type correction

We used the Reinius-based Houseman method*(5, 6)* with the estimateCellCounts function in the Minfi package*(7)* in R*(15)* to estimate relative proportions of six white blood cell subtypes (CD4+ T-lymphocytes, CD8+ T-lymphocytes, NK (natural killer) cells, B-lymphocytes, monocytes and granulocytes).

Batch correction

The covariate batch was also accounted for in the models, based on the bisulfite treatment date at 8 years. At 16 years, the empirical Bayes method via ComBat was applied for batch correction based on sample plate and sentrix position using the sva package in R*(16)* (Johnson et al. 2007)

Exclusion criteria

We applied the following exclusion criteria: multiple births (i.e. singleton only analysis), preterm births (i.e. ≥37 weeks only), maternal diabetes, pre-eclampsia.

California Birth Cohort (CBC)

Design and study population

The California Department of Public Health maintains a repository of all neonatal birth bloods as blood dried on a filter paper (Guthrie card), which we term the California Birth Cohort (CBC). These are available for qualified researchers to perform specified health research as monitored by local and State level institutional review boards; all work here was performed under ethics approval at the State of California and University of California. Our current research project using CBC resources is a case-control study of childhood leukemia, the California Childhood Leukemia Study (CCLS) which identifies children with leukemia and matched

controls (birthdate, gender, and ethnicity) from around the State of California*(17)*. Control subjects were chosen as birthdate, gender, and ethnic matches to leukemia cases. Cases included all consecutive cases at 13 California hospitals which treat leukemia.

Birthweight
Birthweight data was collected by obstetricians in the delivery room and recorded on the birth certificate, which was used for the current analysis. We double checked and did not observe birthweight values +/- 5 SD from the mean. For the main analysis model, we treated birthweight as a continuous trait in grams and for the dichotomous model we categorised birthweight in high (>4000g) versus normal (2500-4000g) birthweight.

Methylation measurements
Approximately 300-500 ng of high molecular weight DNA was extracted from a 1/4 section of a 1.5 cm2 archived neonatal DBS (stored at -20°C from the time of birth) using Qiagen blood card extraction protocol and bisulfite treated using the EZ DNA Methylation-Direct™ Kit (Zymo). Genome-wide DNA methylation was then measured in these bisulfite-converted DNA samples using Illumina(C) Infinium HumanMethylation450 BeadChip arrays. CpGs with detection p-values > 0.01 were defined as bad CpGs and discarded. CpGs with >15% of absence of information (i.e. >15% of total samples) were totally excluded from the analysis. A total of 540 CpGs were excluded. Samples with >15% of bad CpGs (of the 450K loci) were also excluded from the analysis. The DNA methylation data preprocessing consisted of functional normalization according to Fortin et al.*(18)* to control for batch and position effects. Additional correction for probe types was accomplished with BMIQ normalization*(19)*. Joo et al. have demonstrated that DNA methylation measured by the HM450k array on archived dried blood spots is fully correlated with DNA methylation measured by the same platform on same individuals' matched frozen buffy coats (correlation coefficient = 0.99), therefore proving that this material is suitable for DNA methylation analyses*(20)*.

Covariates
Gestational age, gender, trichotomous annual household income <=$44,999/<=$74,999/>=$75,000 as maternal social class, dichotomous maternal smoking status, maternal age and maternal BMI, parity were used as covariates in the models. Models were stratified by ethnicity group (Hispanic and Caucasian).

Cell type correction
Estimation of six different white blood cell types (CD8+ T and CD4+ T lymphocytes, CD56+ natural killer cells, CD19+ B cells, CD14+ monocytes, and granulocytes) by Houseman method*(6)* was performed using the default implementation of the *estimateCellCounts* function in the minfi package*(7)*.

Batch correction
Analyses were adjusted for batch by including the Beadchip ID.

Exclusion criteria
Information on maternal diabetes and pre-eclampsia was not available, thus we were not able to filter data based on these criteria. For this study we only included control participants, participants with leukemia were excluded.

The Center for the Health Assessment of Mothers and Children of Salinas study (CHAMACOS)
Design and study population
The Center for the Health Assessment of Mothers and Children of Salinas (CHAMACOS) study is a longitudinal birth cohort study of the effects of exposure to pesticides and environmental chemicals on the health and development of Mexican-American children living in the agricultural region of Salinas Valley, CA. Detailed description of the CHAMACOS cohort has previously been published*(21, 22)*. Briefly, 601 pregnant women were enrolled in 1999-2000 at community clinics and 527 liveborn singletons were born. Follow up visits occurred at regular intervals throughout childhood. Study protocols were approved by the University of California, Berkeley Committee for Protection of Human Subjects and written informed consent was obtained from all mothers; oral assent was obtained from children beginning at age 7, and written assent at age 12.

Birthweight
Infant birthweight (grams) was collected from medical records and abstracted by a registered nurse. We did not observe birthweight values +/- 5 SD from the mean.

Methylation measurements
DNA methylation was measured in DNA isolated from the cord blood of 378 CHAMACOS newborns, 244 nine year samples, and 60 twelve year samples by Illumina Infinium HumanMethylation450 (450K) BeadChips. DNA samples were bisulfite converted using Zymo Bisulfite Conversion Kits (Zymo Research, Irvine, CA), whole genome amplified, enzymatically fragmented, purified, and applied to the 450K BeadChips (Illumina, San Diego, CA) according to manufacturer protocol. 450K BeadChips were handled by robotics and analyzed using the Illumina Hi-Scan system. DNA methylation was measured at 485,512 CpGs.

Probe signal intensities were extracted by Illumina GenomeStudio software (version XXV2011.1, Methylation Module 1.9) methylation module and background subtracted. QA/QC was performed systematically by assessment of assay repeatability batch effects using 38 technical replicates, and data quality established as previously described [3]. Quality was also ensured by only retaining samples where 95 % of sites assayed had detection p> 0.01. The same threshold (95% detection at p>0.01) was imposed to CpGs as well (n= 460 removed). Sites with annotated probe SNPs and with common SNPs (minor allele frequency >5%) within 50bp of the target identified in the MXL (Mexican ancestry in Los Angeles, California) HapMap population were excluded from analysis (n=49,748). This left a total of 435,369 CpGs in the analysis. Color channel bias, batch effects and difference in Infinium chemistry were minimized by application of ASMN algorithm*(23)*, followed by BMIQ normalization*(19)*.

Covariates
Maternal age, parity and education were assessed by participant interview at baseline visit (~13 weeks gestation). Maternal education was used as a proxy for maternal social class. Maternal education was treated as categorical, with two levels: less than a completed high school education, or having completed high school education or beyond. Maternal pre-pregnancy BMI was calculated using self-reported pre-pregnancy weight when interviewed at enrolment and measured height. Information on maternal smoking status was obtained through participant interview at baseline (~13 weeks gestation), follow up interview (~26 weeks gestation), and delivery.

Cell type correction
We used the Reinius-based Houseman method*(5, 6)* with the *estimateCellCounts* function in the Minfi package*(7)* in R*(8)* to estimate relative proportions of six white blood cell subtypes (CD4+ T-lymphocytes, CD8+ T-lymphocytes, NK (natural killer) cells, B-lymphocytes, monocytes and granulocytes).

Batch correction
Analysis was also adjusted for batch effects by including 450K plate (n=10) as additional covariates.

Exclusion criteria
For the main analysis we excluded n=85 unique participants after overlap: 31 infants born preterm (<37 weeks), and whose mothers experienced pre-eclampsia (n=36) or diabetes (n=80) during pregnancy. This information was obtained from questionnaires completed by the mothers shortly after delivery and combined with medical birth records.

Children's Health Study (CHS)
Design and study population
The Children's Health Study (CHS) is a population-based prospective cohort study from age 5 onwards in Southern California, which has been described in detail elsewhere*(24)*. The study protocol was approved by the University of Southern California Institutional Review Board and informed, written consent and assent were provided by the parents and children respectively. A total of 5341 children were recruited, all of whom were born between 1995 and 1997 and are currently being followed until age 18.

Birthweight
Newborn birthweight was obtained from California birth certificates. We did not observe birthweight values +/- 5 SD from the mean.

Methylation measurements

Based on the availability of newborn bloodspots archived by the state of California, a subset of 273 children was selected for a sub-study in which epigenome-wide DNA methylation was assessed in newborn bloodspots using the Infinium HumanMethylation450 BeadChip (HM450). Laboratory personnel performing DNA methylation analysis were blinded to study subject information. DNA was extracted from whole blood cells using the QiaAmp DNA blood kit (Qiagen Inc, Valencia, CA) and stored at -80 degrees Celcius. 700-1000ng of genomic DNA from each sample was treated with bisulfite using the EZ-96 DNA Methylation Kit™ (Zymo Research, Irvine, CA, USA), according to the manufacturer's recommended protocol and eluted in 18 μl. Chips were analysed in three batches over a period of a couple of months. The results of the Infinium HumanMethylation450 BeadChip (HM450) were compiled for each locus and were reported as beta (β) values. A normal-exponential background correction with dye bias correction was applied to the raw intensities at the array level to reduce background noise*(25)*. We then normalized each sample's methylation values to have the same quantiles to address sample to sample variability*(26)*. CpG loci on the HM450 array were removed from analyses if they were on the X and Y chromosomes, or if they contained SNPs, deletions, repeats, or if they have more than 10% missing values, leaving a total of 383,857 probes for analysis. Beta values were considered as outliers and were removed if they fall below Quartile 1-3×IQR or above Quartile 3+3×IQR.

Covariates
Gestational age at birth, newborn gender, maternal age at birth and maternal parity were obtained from California birth certificates. Data on maternal social class and maternal smoking status during pregnancy were obtained from parent-completed questionnaires at study entry when the subjects were around 6 years old. Ancestry was assessed from CHS genome-wide genotypic

data using the program STRUCTURE from a set of ancestral informative markers that were scaled to represent the proportion of African American, Asian, Native American and white admixture*(27)*. The CHS sample consisted of 55.3% Hispanic white, 32.7% non-Hispanic white, and 12.1% Asian/other acenstries.

Cell type correction
Six white blood cell subtypes were estimated using the Reinius-based Houseman method*(5, 6)*. Estimated cell subpopulations (CD8+ T-lymphocytes, CD4+ T-lymphocytes, natural killer cells, B-lymphocytes, monocytes and granulocytes) were subsequently included as linear predictors in regression models.

Batch correction
We additionally corrected the analyses for batch effect by including the Illumina Infinium HumanMethylation450 BeadChip plate number (n=3).

Exclusion criteria
For the main analysis we excluded n=24 participants: 12 infants born preterm (<37 weeks), and whose mothers experienced pre-eclampsia (n=4) or diabetes (n=5) during pregnancy. This information was obtained from California birth certificates. Additionally, we excluded subjects with missing data on birthweight or any of the other covariates in the model (gestational age, gender, maternal education level, maternal smoking status, maternal age, parity, methylation plate, estimated cell proportions and ancestry), leaving 199 subjects in the analysis.

Early Autism Risk Longitudinal Investigation (EARLI)
Design and study sample
The Early Autism Risk Longitudinal Investigation (EARLI) is an enriched risk prospective pregnancy cohort to study autism etiology*(28)*. The EARLI study was reviewed and approved by Human Subjects Institutional Review Boards (IRBs) from each of the four study sites (Johns Hopkins University, Drexel University, University of California Davis, and Kaiser Permanente Northern California). This longitudinal study recruited mothers of confirmed autism spectrum disorder (ASD) children who were early in a subsequent pregnancy or were trying to become pregnant. There were 232 mothers with a subsequent sibling born through this study. All children were born between November 2009 and March 2012. Demographics, maternal behaviors, food frequency, medical history were all collected via questionnaire. Biosamples and house samples were collected during pregnancy, at birth, and during development. EARLI consisted of 49.6% European, 13.0% Hispanic, 7.6% African and 29.8% other ancestries.

Birthweight
Birthweight was obtained from medical records abstraction. No birthweight values met the exclusion criteria (+/- 5SD).

Methylation measures
Biospecimens including cord blood and placenta were collected and archived at 213 births. Cord blood DNA was extracted using the DNA Midi kit (Qiagen, Valencia, CA) and samples were bisulfite treated and cleaned using the EZ DNA methylation gold kit (Zymo Research, Irvine, CA). DNA was plated randomly and assayed on the Infinium HumanMethylation450 BeadChip (Illumina, San Diego, CA) at the Johns Hopkins SNP Center, a shared lab and informatics operation with the Center for Inherited Disease Research (Johns Hopkins University). Methylation control gradients and between-plate repeated tissue controls were used. We used the minfi library (version 1.18.2) in R (version 3.3) to process raw Illumina image files into noob background corrected methylation values*(18, 25)*. Probes with failed detection P-value (>0.05) in >10% of samples were removed (n=508). Samples with discordant methylation predicted sex and observed sex were removed (n=2) as were samples that appeared as outliers on the first principal component of methylation data across the genome prior to normalization (n=2). Of the remaining 175 cord blood 450k samples, birthweight and complete covariate status were available on 131 participants. This resulted in 2 low birthweight, 16 high birthweight, and 113 normal birthweight children. We excluded control probes, cross-reactive probes*(13)* and probes that did not meet our criteria of a detection p value of the samples, resulting in 455,698 remaining CpGs.

Covariates
Models were adjusted for self-reported maternal education, maternal age, and smoking level. We also adjusted for newborn sex and gestational age, which were obtained from medical records. We further adjusted for ancestry principal components 1 and 2, estimated cell counts (n=6) and hybridization date. By selection, maternal parity for the study was >1 and we did not adjust models for parity.

Cell type correction
Proportion of cell types was estimated using the estimateCellCounts function in the minfi package*(29)* using the Houseman method*(6)* with the adult reference panel*(5)*. Cell types estimated were CD4+ T-lymphocytes, CD8+ T-lymphocytes, NK (natural killer) cells, B-lymphocytes, monocytes and granulocytes.

Batch correction
We adjusted noob normalized data for batch effect by hybridization date using ComBat in the sva package (version 3.9.1)*(30)*.

Exclusion criteria
For the main analysis we excluded n=44 participants: 34 missing birthweight data, 2 sustained smokers, 1 missing gestational age data. No infants were born preterm (<37 weeks). This information was obtained from questionnaires completed by the mothers throughout pregnancy and shortly after delivery and combined with medical birth records.

EXPOsOMICS
Design and study population
Within the EXPOsOMICS collaborative European project, a combination of three population-based birth cohorts ENVIRONAGE, Rhea and Piccolipiu was established to conduct DNA methylation analyses*(31)*. The phenotypic variables were harmonized across the three cohorts, and their biospecimen were randomized on the DNA methylation arrays.

Piccolipiu: Piccolipiu is a multicentric Italian birth cohort that recruited 3338 newborns and their mothers in 5 centres: Turin, Trieste, Florence, Viareggio and Rome between 2011 and 2015. Details about the study protocol have been published elsewhere*(32)*. Families were contacted 6, 12, 24 and 48 months after delivery to collect follow-up information using questionnaires, and children underwent a medical examination at 4 years of age. Cord blood was collected and stored in a centralized biobank. Ethical approvals have been obtained from the Ethics committees of the Local Health Unit Roma E (management centre), of the Istituto Superiore di Sanità (National Institute of Public Health) and of each local centre. Parents provided written informed consent. A sample of 99 children from the Turin centre -who were resident in Turin, with growth data at birth and until at least 2 years of age and full availability of cord blood samples- was included in the EXPOsOMICS Children Studies.

Rhea: The mother-child “Rhea” study in Crete is a prospective cohort examining a population sample of pregnant women and their children, at the prefecture of Heraklion (n = 1500)*(33)*. Ethical approval was obtained and informed consent was given. The study aims are to evaluate a) nutritional, environmental, biological and psychosocial exposures in the prenatal period and in early childhood, b) the association of these exposures with the development of the foetus and the child, c) mother’s health during and after pregnancy, and d) genetic susceptibility and the interactions between genetic and environmental factors affecting child health. A set of 100 children from the RHEA-cohort is included in the EXPOsOMICS children studies, for which data on cord blood DNA-methylation is available.

ENVIRonAGE: The ENVIRonAGE (ENVIRonmental influence ON AGEing in early life) cohort includes 1500 mother-infant pairs (Belgium) and further recruitment is ongoing*(34)*. Ethical approval was obtained and written informed consent was given by the parents. Data include mothers’ lifestyle and socio-economic status, gestational history, measurements including the new-borns’ blood pressure (all healthy), bio-banked placental tissue and cord blood including RNA/DNA, toxic metals in cord blood and placenta, and in utero and early life exposure to fine particulates and NO2 using a spatial temporal interpolation method. A set of 200 children from the ENVIRonAGE-cohort is included in the EXPOsOMICS children studies, for which data on cord blood DNA methylation is available.

Birthweight
Piccolipiu: Newborn’s birthweight was abstracted from obstetric records. Outliers were identified through inspection of the joint distribution of birthweight and gestational duration. We did not observe birthweight values +/- 5 SD from the mean. For the main analysis model, we treated birthweight as a continuous trait in grams, and, for the dichotomous model, we categorised birthweight in high (>4000g) versus normal (2500-4000g) birthweight.

Rhea: Newborn’s birthweight was abstracted from obstetric records. Outliers were identified through inspection of the joint distribution of birthweight and gestational duration. We did not observe birthweight values +/- 5 SD from the mean. For the main analysis model, we treated birthweight as a continuous trait in grams, and, for the dichotomous model, we categorised birthweight in high (>4000g) versus normal (2500-4000g) birthweight.

ENVIRonAGE: Newborn’s birthweight was abstracted from obstetric records. Outliers were identified through inspection of the joint distribution of birthweight and gestational duration. We did not observe birthweight values +/- 5 SD from the mean. For the main analysis model, we treated birthweight as a continuous trait in grams, and, for the dichotomous model, we categorised birthweight in high (>4000g) versus normal (2500-4000g) birthweight.

Methylation measurements
The size of the selected sample set from EXPOsOMICS birth cohorts is 399 newborns: Environage (n=200), Picolli+ (n=99) and RHEA (n=100). From these newborns, cord blood DNA was used for the epigenome-wide DNA methylation analyses, and the samples were randomized across the three cohorts before the subsequent processing steps. Bisulfite conversion (600 ng DNA/sample) was performed using the EZ-96 DNA methylation kit (Zymo research Corporation, Irvine, USA). Then the Infinium HumanMethylation450 BeadChip (Illumina Inc., San Diego, USA) was used to measure the methylation level as a beta value ranging from 0 (no methylation) to 1 (complete methylation). All samples passed the quality control. However, we identified 10 samples

as potential gender mismatches which we further excluded in the analysis. The distributions of raw data were already coherent across samples before functional normalization*(18)*, which was done using preprocessFunnorm function from the minfi R package*(29)*. We filtered out cross-reactive probes and sex-chromosome probes. The final number of CpGs that was analysed was 470964. We included a cohort-specific variable in our analysis, for which we adjusted for in order to remove residual cohort-specific effects following the data harmonization and biospecimen randomization. The data was generated at the Epigenetics Group, International Agency for Research on Cancer, Lyon, France.

Covariates
Data on maternal parity, socio-economic status, smoking, pre-pregnancy body mass index, age at birth, newborn gender and gestational age at birth and cohort were self-reported in questionnaires during pregnancy. Based on place of birth of mother and father, approximately 97% of the participants were of European ethnicity.

Cell type correction
We used the Reinius-based Houseman method*(5, 6)* with the *estimateCellCounts* function in the Minfi package*(29)* in R to estimate relative proportions of six white blood cell subtypes (CD4+ T-lymphocytes, CD8+ T-lymphocytes, NK (natural killer) cells, B-lymphocytes, monocytes and granulocytes).

Batch correction
We additionally corrected the analyses for batch effect using surrogate variable analysis (SVA).

Exclusion criteria
In the methylation subset of the EXPOsOMICS cohort we excluded infants born preterm (<37 weeks) (n=28), whose mothers experienced pre-eclampsia (n=2) or diabetes during pregnancy (n=24), from whom we that didn't have complete data for covariates (n=15) and the potential gender mismatches (n=10). The total number of exclusions is 75 because there are 4 overlaps between different exclusion criteria. This information was obtained from questionnaires completed by the mothers shortly after delivery and combined with medical birth records.

Flemish Environment and Health Study (FLEHS1)
Design and study population
The Flemish Environment and Health Study I (FLEHSI) was established for human biomonitoring in different geographically representative samples of the Flemish population in Belgium*(35, 36)*. The FLEHS I birth cohort consists of 1196 mother–child pairs whom were systematically recruited between September 2002 and February 2004 via 25 maternities across Flanders; covering 20% of Flanders' area and 65 different municipalities. Cord blood samples were collected at delivery. Details of the recruitment protocol have been previously reported*(37)*. Inclusion criteria were living for at least five years in the area of interest and being able to fill out Dutch questionnaires. Informed consent was provided by all participating mothers and the campaign was approved by the ethical committee of the University of Antwerp. During follow-up of the cohort at 10 years of age (n=595) data on growth, diet, physical activity, medical conditions and life-style were gathered. A subcohort (n=99) of those followed-up at age 10 years agreed to provide blood samples plus questionnaire data at the age of 11 years.

Birthweight
The outcome of interest was birthweight, which was recorded in grams at birth among other clinical parameters (such as length, Apgar score). We did not have to excluded any of the children based on birthweight being out of range, and there were no missing values.

Methylation measurements
Peripheral blood mononuclear cells (PBMC) were isolated from the blood collected at age 11 years using Lymphoprep™ (Axis-Shield, Oslo, Norway). Whole genomic DNA was isolated from the PBMC fraction. From the 99 blood samples, 92 DNA samples passed the DNA quality check. About 500 ng of gDNA was bisulfite converted using the EZ DNA methylation kit (Zymo Research, Cambridge Bioscience, Cambridge, UK) according to manufacturer's instructions. Genome-wide DNA methylation profiles were generated with Infinium HumanMethylation450 BeadChip Array (Illumina, San Diego, CA, USA) according to the standard Infinium HD Assay Methylation Protocol Guide (Part #15019519, Illumina). The raw methylation intensities for each probe were represented as methylation β-values (ranging from 0, unmethylated, to 1, fully methylated) and extracted from GenomeStudio Methylation Module software without background correction and normalization. Raw data analysis, QC and normalization were performed using "minfi" R-package. In brief, the raw Red/Green channel data from the 450K-llumina methylation array were read by the 'read.450k.exp' function, converted to methylation values by 'preprocessRaw' and subsequently normalized using 'preprocessSWAN', an implementation of the Subset-quantile Within Array Normalization (SWAN) normalization procedure*(38)*. Principal component analysis and unsupervised clustering were used to check for sample outliers (i.e. 2 because of bad quality). All samples that passed quality controls were loaded into the IMA-package used for further processing. Samples having >75% of CpGs with a detection p-value > 1e-05 were removed (all samples passed this filer). Cg-probes with a detection p-value greater than 0.01 in all samples and cg-probes on the X and Y chromosome were removed. 470,562 sites were retained from the original 485,512 sites.

Covariates
Covariate data (i.e. maternal parity, socio-economic status, smoking, pre-pregnancy body mass index, age at birth, newborn gender and gestational age at birth) were obtained from questionnaires and medical record information for children and their mothers. Maternal smoking status was not included as a covariate in the dichotomous analysis, since none of the mothers of children in the high birthweight group had smoked during pregnancy.

Cell type correction
To estimate the proportion of various cell types in the PBMC samples the statistical deconvolution method described by Houseman and colleagues and implemented in minfi-package as the 'estimateCellCountsMset' function was used*(6)*. Reference methylomes from leukocyte subtypes were obtained from the study of Reinius *et al.(5)*

Batch correction
Analyses were additionally adjusted for batch effects by including the batch number (n=2) since there was a gap of a year in between the analyses of 2 batches of samples.

Exclusion criteria
In total 5 participants were excluded: 3 preterm birth (i.e. <37 weeks), 1 maternal diabetes, and 1 pre-eclampsia case. Data on preterm birth, maternal diabetes and pre-eclampsia during pregnancy were obtained from questionnaires completed by the mothers directly after delivery.

GECKO Drenthe

Design and study population
The Groningen Expert Center for Kids with Obesity (GECKO) Drenthe cohort is a population-based prospective birth cohort study in Drenthe, a northern province in the Netherlands. All mothers of infants born between April 2006 and April 2007 were invited to participate during the third trimester of pregnancy. Of all 4,778 infants born in this period, a total of 2,874 newborns (60%) participated in the study. This study has been approved by the Medical Ethical Committee of the University Medical Center Groningen and parents of all participants gave written informed consent. Details about this cohort have been described elsewhere*(39)*.

Birthweight
Newborn birthweight was recorded by obstetricians or midwives in the delivery room or abstracted from obstetric records. We double checked and did not observe birthweight values +/- 5 SD from the mean.

Methylation measurements
Within the GECKO Drenthe birth cohort, we selected 258 infants for the methylation study: 129 exposed to maternal smoking during pregnancy and 129 unexposed to both maternal and paternal smoking during pregnancy*(40)*. From these 258 infants, we used DNA which was extracted from cord blood for the epigenome-wide DNA methylation analyses. To limit batch effects, we randomized all samples over the 96-well plates, based on gender and smoking status. Samples (500 ng per sample) were placed on three 96-well plates. Bisulfite conversion was performed using the EZ-96 DNA methylation kit (Zymo research Corporation, Irvine, USA). Then we used the Infinium HumanMethylation450 BeadChip (Illumina Inc., San Diego, USA) to measure the methylation level as a beta value ranging from zero (no methylation) to one (complete methylation). During the quality control, we excluded two males that clustered in the female group, based on X chromosome betas, which was probably due to maternal blood contamination. We performed Illumina-suggested background normalization, colour correction and Subset-quantile Within Array Normalization (SWAN). We excluded one sample because it did not meet the criteria of ≥99% of the CpGs with detection p value <0.05. This resulted in 129 exposed and 126 unexposed children. We excluded control probes, probes on X or Y chromosomes and probes that did not meet our criteria of a detection p value of <0.05 in ≥99% of the samples, resulting in 465,891 remaining CpGs.

Covariates
Data on maternal parity, socio-economic status, smoking, pre-pregnancy body mass index, age at birth, newborn gender and gestational age at birth were self-reported in questionnaires during pregnancy.

Cell type correction
We used the Reinius-based Houseman method*(5, 6)* with the *estimateCellCounts* function in the Minfi package*(7)* in R*(8)* to estimate relative proportions of six white blood cell subtypes (CD4+ T-lymphocytes, CD8+ T-lymphocytes, NK (natural killer) cells, B-lymphocytes, monocytes and granulocytes).

Batch correction
We additionally corrected the analyses for batch effect by including the Illumina Infinium HumanMethylation450 BeadChip number (n=3).

Exclusion criteria

In the methylation subset of the GECKO Drenthe cohort we excluded infants born preterm (<37 weeks), and whose mothers experienced pre-eclampsia or diabetes during pregnancy, this information was obtained from questionnaires completed by the mothers shortly after delivery and combined with medical birth records.

The Genetics of Glucose regulation in Gestation and Growth (Gen3G)

Design and study population

The Genetics of Glucose regulation in Gestation and Growth (Gen3G) is a population-based prospective birth cohort study in Sherbrooke, Qc, Canada. Between January 2010 and June 2013, we invited pregnant women aged ≥18 years old who visited the blood sampling in pregnancy clinic in Sherbrooke for their first trimester clinical blood samples. 1034 women accepted to participate in our cohort study. This study has been approved by the CHUS Ethics Review Board for Studies with Humans and every participant gave written informed consent before enrolment in the study. Details about this cohort have been described elsewhere*(41)*.

Birthweight

We collected birthweight from clinical obstetric records. We verified and did not observe birthweight values +/- 5 SD from the mean.

Methylation measurements

Within the Gen3G birth cohort we randomly selected 182 infants for the methylation study in the single pregnancies without gestational diabetes. From these 182 infants, we used DNA which was extracted from cord blood for the epigenome-wide DNA methylation analyses. To limit batch effects, we randomly placed all samples (500 ng per sample) over 96-well plates. Bisulfite conversion was performed using the EZ-96 DNA methylation kit (Zymo research Corporation, Irvine, USA). We used the Infinium HumanMethylation450 BeadChip (Illumina Inc., San Diego, USA) to measure methylation levels as a beta value ranging from 0 (no methylation) to 1 (complete methylation). During quality control procedures, we excluded 5 participants that clustered in the wrong sex group and one that was considered an outlier from the MDS plot. We excluded 6 samples because they did not meet the criteria of ≥99% of the CpGs with detection p value <0.01. Finally, we removed 8 samples from this analysis, because of missing values (n=6) or because they were not born at term (n=2). Thus, we conducted this analysis in 162 children. We performed DASEN normalization from the watermelon package. We excluded probes that did not meet our criteria of a detection p value of <0.01 in ≥80% of the samples, resulting in 482,992 remaining CpGs.

Covariates

Data on maternal parity, smoking, age at birth were self-reported in questionnaires during first trimester of pregnancy. Maternal pre-pregnancy BMI was calculated with self-reported pre-gestational weight and height measured at first trimester. Newborn gender and gestational age at delivery were abstracted from obstetric records. Maternal social class was not available in our study.

Cell type correction

We used the Reinius-based Houseman method*(5, 6)* with the *estimateCellCounts* function in the Minfi package*(7)* in R*(8)* to estimate relative proportions of six white blood cell subtypes (CD4+ T-lymphocytes, CD8+ T-lymphocytes, NK (natural killer) cells, B-lymphocytes, monocytes and granulocytes).

Batch correction

We additionally corrected the analyses for batch effect by including the Illumina Infinium HumanMethylation450 Sample plate number in our regression analyses.

Exclusion criteria

For the main analysis we excluded n=2 infants born preterm (<37 weeks). This information was obtained from medical birth records. For our study using Illumina Infinium HumanMethylation450 we had a priori decided to select samples from babies born from mothers who did not develop gestational diabetes (based on oral glucose tolerance test) and without pre-eclampsia (medical records).

The Generation R Study

Design and study population

The Generation R Study is a prospective population-based cohort in Rotterdam, the Netherlands*(42)*. All pregnant women residing in Rotterdam with a delivery date between April 2002 and January 2006 were invited to participate. The Medical Ethical Committee of Erasmus MC, University Medical Center Rotterdam, approved the study and an informed consent was obtained for all participating children. In total, 9,778 mothers were enrolled in the study*(42)*.

Birthweight

Birthweight was obtained from community midwife and hospital registries. All birthweight values were within +/-5 SD from the mean. Birthweight was analysed in grams.

Methylation measurements

DNA extracted (using the salting-out method) from cord blood and blood samples from children aged around 10 years of European-ancestry was used for this analysis. 500 ng DNA per sample underwent bisulfite conversion using the EZ-96 DNA Methylation kit (Shallow) (Zymo Research Corporation, Irvine, USA). Samples were plated onto 96-well plates in no specific order. Samples were processed with the Illumina Infinium HumanMethylation450 BeadChip (Illumina Inc., San Diego, USA), which analyses methylation at 485,577 CpGs. Quality control of analyzed samples was performed using standardized criteria, separately for the cord blood samples and the child samples, as the latter were added at a later stage.

Quality control and normalisation of cord blood samples
For the cord blood samples, reasons for sample exclusions were sample call rate <99%, colour balance >3, low staining efficiency, poor extension efficiency, poor hybridization performance, low stripping efficiency after extension, poor bisulfite conversion and gender mismatch. After quality control, 969 samples remained in the analysis. Probes with a single nucleotide polymorphism in the single base extension site with a frequency of >1% in the GoNLv4 reference panel were excluded*(43)*, as were probes with non-optimal binding (non-mapping or mapping multiple times to either the normal or the bisulphite-converted Genome*(44)*, resulting in the exclusion of 49,564 probes, leaving a total of 436,013 probes in the analysis. We ran DASES normalization using a pipeline adapted from that developed by Touleimat and Tost*(26)*. DASES normalization includes background adjustment, between-array normalization applied to type I and type II probes separately, and dye bias correction applied to type I and type II probes separately and is based on the DASEN method described by Pidsley *et al*. but adds the dye bias correction, which is not included in DASEN*(45)*.

Quality control and normalisation of child blood samples
Preparation and normalization of the HumanMethylation450 BeadChip array data was performed according to the CPACOR workflow[1] using the software package R[2]. In detail, the idat files were read using the minfi package. Probes that had a detection p-value above background (based on sum of methylated and unmethylated intensity values) ≥ 1E-16 were set to missing per array. Next, the intensity values were stratified by autosomal and non-autosomal probes and quantile normalized for each of the six probe type categories separately: type II red/green, type I methylated red/green and type I unmethylated red/green. Beta values were calculated as proportion of methylated intensity value on the sum of methylated+unmethylated+100 intensities. Arrays with observed technical problems such as failed bisulfite conversion, hybridization or extension, as well as arrays with a mismatch between sex of the proband and sex determined by the chr X and Y probe intensities were removed from subsequent analyses. Additionally, only arrays with a call rate > 95% per sample were processed further. The final dataset contained 425 samples at age 10.

Covariates
Data on maternal age, educational level and parity were collected by questionnaires in early pregnancy. Maternal body mass index was assessed at intake. Maternal smoking during pregnancy was assessed by questionnaires in each trimester of pregnancy. Gestational age and sex of the child were determined by ultrasound during pregnancy as described previously*(46)*. Current age and BMI of the children included in the sensitivity analysis were recorded/measured at our research center.

Cell type correction
Cell type correction was applied using the reference-based Houseman method*(6)* in the minfi Package*(29)* in R*(8)*. This method estimates the relative proportions of six white blood cell subtypes (CD4+ T-lymphocytes, CD8+ T-lymphocytes, NK (natural killer) cells, B-lymphocytes, monocytes and granulocytes), based on a standard reference population*(5)*.

Batch correction
We additionally corrected the analyses for batch effect by adjusting for plate number.

Exclusion criteria
For the main analysis we excluded n=165 participants: 23 infants born preterm (<37 weeks) and 49 postterm (>42 weeks), and whose mothers experienced pre-eclampsia (n=81) or diabetes (n=12) during pregnancy. This information was obtained from questionnaires completed by the mothers shortly after delivery and combined with medical birth records.

Glycyrrhizin in Licorice (Glaku)
Design and study population
The study participants were included from an urban community-based birth cohort Glycyrrhizin in Licorice (GLAKU)*(47)*. It is a prospective study cohort of 1049 infants born between March and November of 1998 at the Helsinki University Hospital and Helsinki City Maternity Hospital, Finland. A primary objective of the cohort was to examine the effects of maternal licorice consumption during pregnancy on their offspring's developmental outcomes. Between 2009 and 2011, all 920 cohort members who had given permission to be contacted and whose addresses were traceable were invited to participate in a follow-up study; 692 (75.2%) were contacted by phone, and 451 (65.2% of the women who were contacted) participated. Of the offspring of the participants to the follow-up, 240 children donated blood and there were 216 children (54.2% girls) in the final analytical sample. Their mean age was 12.4 years (SD=0.5, range 11.1-13.1 years). Details of the initial sample collection and follow-up in 2009-2011 have been reported elsewhere*(47, 48)*. The study protocol was approved by the ethical committees of the City of Helsinki and the Uusimaa Hospital District. Written informed consent was obtained from the mothers after delivery and from the parent/guardian and adolescent at the follow-up assessment.

Birthweight
Infant weight (kg) was measured immediately after birth with a standard electronic scale by midwives in the delivery room or abstracted from obstetric records. There were no birthweight values +/- 5 SD from the mean.

Methylation measurements
Blood was drawn in 2009-2011 visit in participating children and DNA was extracted from for the epigenome-wide DNA methylation analyses. Bisulfite conversion was performed using the EZ-96 DNA methylation kit (Zymo research Corporation, Irvine, USA). 240 blood samples were ran on Illumina EPIC Methylation arrays (Illumina Inc., San Diego, USA). The quality control pipeline was set up using the R-package minfi. Methylation beta-values were normalized using the funnorm function. One IDs showed density artefacts after normalization and was removed from further analysis. We excluded any probes on chromosome X or Y, probes containing SNPs and cross-hybridizing probes according to Chen *et al.(13)*, Price *et al.(49)* and McCartney *et al.(50)* Furthermore, any CgGs with a detection p-value > 0.01 in at least 25% of the samples were excluded. The final dataset contains 812,943 CpGs and 239 IDs.

Covariates
Data on newborn gender was extracted from the social security number and gestational age at birth was estimated based on ultrasound. Data on smoking, pre-pregnancy body mass index, parity, and age at birth were self-reported in questionnaires during pregnancy. In addition, data on maternal education, child BMI, and age at the follow-up were self-reported. We also used first three MDS components based on genome-wide genotyping with Illumina OmniExpressExome 1.2 array data to adjust for population stratification.

Cell type correction
We used the Reinius-based Houseman method*(5, 6)* with the *estimateCellCounts* function in the Minfi package*(7)* in R*(8)* to estimate relative proportions of six white blood cell subtypes (CD4+ T-lymphocytes, CD8+ T-lymphocytes, NK (natural killer) cells, B-lymphocytes, monocytes and granulocytes).

Batch correction
We used ComBat to check and adjust for the batch effects (row).

Exclusion criteria
For the main analysis we excluded n=7 due to preterm birth (<37 weeks based on ultrasound estimate of gestational age), which is 2.9% of the Glaku participants with methylation and birthweight data. This information was obtained from medical birth records.

Genome-Wide Population-based Association Study of Extremely Overweight Young Adults (GOYA)
Design and study population
The Genome-Wide Population-based Association Study of Extremely Overweight Young Adults (GOYA) study is described previously by Paternoster *et al.(51, 52)* It is based on the Danish National Birth Cohort (DNBC) that included 92,000 pregnant women and their pregnancies during 1996-2002. Of 67,853 women who had given birth to a live born infant, had provided a blood sample during pregnancy and had BMI information available, 3.6% of these women with the largest residuals from the regression of BMI on age and parity (all entered as continuous variables) were selected for GOYA. The BMI for these 2451 women ranged from 32.6 to 64.4. From the remaining cohort a random sample of similar size (2450) was also selected. DNA methylation data were generated for the offspring of 1000 mothers in the GOYA study. I.e. "cases" had mothers with a BMI>32 and "controls" were sampled from the normal BMI distribution (can include mothers with a BMI>32). All participants in the DNBC gave written informed consent and the collection and use of their data has ethics approval. Ethnicity was obtained using register data.

Birthweight
Information on birthweight was recorded by healthcare professionals at the time of birth and extracted from birth records within the National Hospital Discharge Registry. Within GOYA, there were no values +/- 5SD from the mean.

Methylation measurements
Cord blood was collected according to standard procedures, spun and frozen at -80˚C. DNA methylation analysis and data pre-processing were performed at the University of Bristol. Following extraction, DNA was bisulfite converted using the Zymo EZ DNA MethylationTM kit (Zymo, Irvine, CA). Following conversion, the genome-wide methylation status of over 485,000 CpGs was measured using the Illumina Infinium® HumanMethylation450k BeadChip assay according to the standard protocol. The arrays were scanned using an Illumina iScan and initial quality review was assessed using GenomeStudio (version 2011.1). The level of methylation is expressed as a "Beta" value (β-value), ranging from 0 (no cytosine methylation) to 1 (complete cytosine methylation). Samples from all time-points in ARIES were distributed across slides using a semi-random approach (sampling criteria were in place to ensure that all time-points were represented on each array) to minimize the possibility of confounding by batch effects. Samples failing quality control (average probe detection p-value ≥ 0.01) were repeated. As an additional quality control step genotype probes on the HumanMethylation450k were compared between samples from the same individual and against SNP-chip data to identify and remove any sample mismatches. Data were normalized using the functional normalization approach in the Minfi R package.

We removed probes that had a detection P-value >0.05 for >5% of samples, probes on the X or Y chromosomes and SNPs (rs probes). 473864 probes remained.

Covariates
Data on maternal parity, socio-economic status, smoking and pre-pregnancy body mass index were collected via a telephone interview at around 16 weeks' gestation. Maternal age was derived from the mother's report of her own date of birth. Newborn sex and gestational age at birth were extracted from birth records.

Cell type correction
We used the Reinius-based Houseman method*(5, 6)* with the *estimateCellCounts* function in the Minfi package*(7)* in R*(8)* to estimate relative proportions of six white blood cell subtypes (CD4+ T-lymphocytes, CD8+ T-lymphocytes, NK (natural killer) cells, B-lymphocytes, monocytes and granulocytes).

Batch correction
Ten surrogate variables were generated using the sva package in R and included in models to adjust for technical batch.

Exclusion criteria
For the main analysis we excluded n=25 participants: 12 infants born preterm (<37 weeks), and infants whose mothers experienced pre-eclampsia (n=10) or diabetes (n=5) during pregnancy, there was some overlap between these. This information was obtained from questionnaires completed by the mothers shortly after delivery and combined with medical birth records.

Healthy Start
Design and study population
The Healthy Start study is an ongoing, prospective pre-birth cohort in Colorado, USA*(53, 54)*. Eligible pregnant women were recruited from the University of Colorado Hospital Outpatient Pavilion, 2009-2014. Eligibility criteria were: age 16 years or older, singleton pregnancy, no prior stillbirths, and gestational age < 24 weeks. We recruited 1,410 pregnant women, approximately 50% of those eligible. Study procedures were approved by the Colorado Multiple Institutional Review Board and written informed consent was obtained from all participating mothers.

Birthweight
Birthweight was obtained from the medical record at delivery.

Methylation measurements
Umbilical cord blood was collected at delivery. Eligibility for DNA methylation analysis was determined by the availability of cord blood and maternal blood and urine samples during pregnancy. Methylation analysis of cord blood samples (N=600) was conducted using the Illumina Infinium HumanMethylation450 BeadChip, and processed in the University of Colorado Genomics Core lab. Samples with predicted child sex inconsistent with reported sex were excluded. Probes with high detection p-value (>0.05) or low beadcount (<3 in at least 5% of the samples) were excluded. All analyses were conducted in R version 3.3.0*(8)*. The preprocessQuantile function in the R package Minfi*(29)* was used for normalization. The total number of CpGs analysed was 484,261.

Covariates
Models were stratified by maternal race/ethnicity group (non-Hispanic white, Hispanic, or non-Hispanic African American). Models were adjusted for the following covariates: gestational age at delivery and infant sex (obtained from the medical record at delivery), maternal age, parity, pre-pregnancy BMI, education completed, smoking during pregnancy (obtained from the mother via questionnaire or from the prenatal medical record).

Cell type correction
We used the estimateCellCounts function in Minfi to estimate relative proportions of six white blood cell subtypes (CD4+ T-lymphocytes, CD8+ T-lymphocytes, NK (natural killer) cells, B-lymphocytes, monocytes and granulocytes).

Batch correction
We corrected for batch effects using ComBat*(16)*.

Exclusion criteria
For the main analysis we excluded n=19 infants born preterm (<37 weeks), and/or whose mothers experienced pre-eclampsia (n=21) or diabetes (n=33) during pregnancy, and 80 participants missing information on maternal smoking during pregnancy. We additionally excluded n=28 participants who belonged to racial/ethnic groups other than the 3 major groups, due to small numbers. This information was obtained from questionnaires completed by the mothers shortly during pregnancy or shortly after delivery, and combined with information from medical birth records. Finally, we excluded 4 participants for whom reported child sex was inconsistent with predicted child sex based on DNA methylation. The total sample size after exclusions was 432 (240 non-Hispanic white, 115 Hispanic, 77 non-Hispanic African American).

Infancia y Medio Ambiente (INMA)

Design and study population

The INMA—INfancia y Medio Ambiente—(Environment and Childhood) Project is a network of birth cohorts in Spain that aims to study the role of environmental pollutants in air, water and diet during pregnancy and early childhood in relation to child growth and development*(55)*. Mothers were enrolled at week 12 of pregnancy from 1997 to 2008 in seven regions of Spain (Flix, Granada, Menorca, Asturias, Gipuzkoa, Sabadell and Valencia). The cohort consisted of 3,768 children at birth. During the follow-up visits information on environmental exposures and health outcomes (reproductive, growth and obesity, lung function, allergies and neurovelopment) were assessed through questionnaires, biomarker measurements, clinical data, and physical exploration. The study website contains details of the design and data available in INMA project (http://www.proyectoinma.org/). The study was approved by the Ethical Committees of each participating centre and written consent was obtained from parents.

In this study, DNA methylation data was available for 166 cord blood samples from INMA Sabadell subcohort.

Birthweight

Birthweight was recorded by specially trained midwives at delivery. We did not observe birthweight values +/- 5 SD from the mean for the gestational age or sex. We only participated in the main analysis using birthweight as continuous. The dichotomous analyses were excluded of this paper as only seven of our children were categorized as high (>4000g) birthweight.

Methylation measurements

Cord blood was extracted using the Chemagen kit (Perkin Elmer). DNA concentration was determined by a NanoDrop spectrophotometer (Thermo Scientific) and with the Quant-iT PicoGreen dsDNA Assay Kit (Life Technologies).

Blood methylation data was produced in the Genome Analysis Facility of the University Medical Center Groningen (UMCG) in Holland as part of the MeDALL (Mechanisms of the Development of Allergy) project. Samples were randomized and processed following the Illumina protocol for the Infinium HumanMethylation450 BeadChip. Briefly, 500 ng of DNA was bisulfite-converted using the EZ 96-DNA methylation kit, and DNA methylation was measured through hybridization on the BeadChips.

DNA methylation data were pre-processed in R*(8)* using minfi*(29)*, from the original idat files extracted from the HiScanSQ scanner. Samples that did not provide significant methylation signals in more than 10% of probes (detection P>0.01) were excluded from further analysis. Samples were also excluded in cases of low staining efficiency, low single base extension efficiency, low stripping efficiency of DNA from probes after single base extension, poor hybridization performance, poor bisulphite conversion and high negative control probe staining. Further, we assessed the methylation distribution of the X-chromosome to verify gender. Finally, we normalized the beta values using dasen*(45)*. We removed 65 SNPs probes, the probes on sex chromosomes, potential cross reactive probes, and probes containing SNPs at the target CpGs with a MAF>10% (polymorphic) were excluded*(13)*. A total of 166 subjects and 439,306 CpG probes were analyzed.

Covariates

We calculated gestational age from the date of the last menstrual period (LMP) reported at recruitment and confirmed using estimates based on the first ultrasound examination (about 12th week of gestation). When the difference between the LMP reported at recruitment and estimated from the ultrasound was ≥ 7 days, we estimated LMP using the crown-rump length*(56)*. Data on age at birth, and maternal height and pre-pregnancy weight (for BMI calculations) were collected from a self-reported using a questionnaire at enrolment (week 12 of pregnancy). Maternal socioeconomic status was based on maternal occupation at pregnancy, it was categorized into three levels: low (levels V/VI semi-skilled/unskilled occupations), medium (levels III/IV skilled manual/non-manual) or high (managers/technicians). Pregnant women were asked whether they were current smokers (at week 32 of pregnancy) and if so, how much. They were also asked if they had stopped smoking due to pregnancy and when (before pregnancy or at what month of pregnancy). Any smoking was defined as smoking any number of cigarettes at any time during pregnancy.

Cell type correction

We used the *estimateCellCounts* function from minfi which estimates a constrained projection using quadratic programming*(6)* we estimated six white blood cell subtypes (CD4+ T-lymphocytes, CD8+ T-lymphocytes, NK (natural killer) cells, B-lymphocytes, monocytes and granulocytes) using the Reinius reference*(5)*.

Batch correction

Batch correction was attained including the significant (permutation p-value< 10^{-4}) principal components derived from the 613 negative control probes presented in 450K arrays. After 10.000 permutations 5 PCs were retained. The beta-values were batch corrected incorporating these 5 PCs and calculating the residuals of the linear model.

Exclusion criteria

For the main analysis we excluded n=17 participants: 5 infants born preterm (<37 weeks), and whose mothers experienced pre-eclampsia (n=6) or diabetes (n=15) during pregnancy. This information was obtained from questionnaires completed by the mothers shortly after delivery and combined with medical birth records.

Gene expression analysis
INMA gene expression data was collected at 4 years of age. Whole blood was collected in PAXGene tubes and extracted using the kit recommended by the company. All samples had a RNA Integrity Number (RIN) higher than seven. Gene expression data was obtained using Affymetrix Human Transcriptome Array 2.0 at the European Institute for Systems Biology and Medicine in Lyon as part of the MeDALL project. Gene expression was normalized with the RMA algorithm using the ExpressionConsole Software from Affymetrix and probes were clustered to the transcript level. Expression transcripts were annotated using version 36 of Affymetrix annotation. Four samples were excluded because they were outliers defined as more than 3 SD from the mean for PC1 or PC2 (N=4). The final sample size was 112.

DNA methylation and DNA extraction has been described elsewhere in the text. In brief, the beta-values that passed the quality control filtering were background corrected using methylumi-noob and functionally normalized*(18)*. As different laboratory batches were processed for this specific analysis, we applied ComBat*(16)* to reduce this undesired technical variability. Next, the effect of sex and blood cell proportions estimates were controlled out in a linear regression model. Cell proportions were estimated using the constrained projection quadratic programming (CP/QP) approach of Houseman*(6)*, using the estimateCellCounts function in the minfi R package*(8, 29)*. We used the Reinius panel as the reference*(5)*.

Only transcripts inside a 500 kb window of a selected CpG (250 kb downstream and 250 kb upstream) were considered in the analysis. The model was controlled for technical and unwanted biological variation when estimating gene expression residuals, 13 surrogate variables were detected and added to the model including sex and 6 cell estimates. Surrogate variables were estimated using the sva R package*(57)*. Cell estimates used the Reinius dataset as the reference*(5)*.

Isle of Wight Birth Cohort (IOW F1)
Design and study population
A whole population birth cohort was established on the Isle of Wight, UK, in 1989 to prospectively study the natural history of allergic diseases from birth onwards*(58)*. Both the Isle of Wight and the study population are 99% Caucasian. Ethics approvals were obtained from the Isle of Wight Local

Research Ethics Committee (now named the National Research Ethics Service, NRES Committee

South Central –Southampton B) at recruitment and for the 1, 2, 4, 10 and 18 years follow-up. Of the

1536 children born between January 1, 1989, and February 28, 1990, written informed consent was obtained from parents to enroll 1456 newborns. Children were followed up at the ages of 1 (n =

1167), 2 (n = 1174), 4 (n = 1218), 10 (n = 1373), and 18 years (n = 1313). Demographic information of parents and offspring, status of allergic diseases, phenotypic measures on allergic sensitization, IgE, and lung function, and environmental exposures, along with other phenotypic measures, were collected at birth and updated at each follow-up.

Birthweight
Newborn birthweight was collected from stored clinical records. The outliers for observe birthweight values +/- 5 SD from the mean was checked. For the main analysis model birthweights were treated as continuous variables in grams and for the dichotomous model birthweights were categorised in high (>4000g) versus normal (2500-4000g) birthweight.

Methylation measurements
For 367 aged 18 years subjects, we measured DNA methylation from whole blood processed with the Illumina Infinium HumanMethylation450 BeadChip (Illumina Inc., San Diego, USA). CPACOR*(59)* pipeline was used for QC and normalisation of the data. Methylation markers on 65 single nucleotide polymorphism (SNP) and sex chromosomes were removed. We applied Illumina background

Correction to all intensity values. Any intensity values having detection p-values >= 10-16 were set as missing data. Samples with call rate < 98% were excluded. After the QC, 461,230 sites remain for the subsequent analysis. A quantile normalisation was applied using limma on intensity values

separately based on six different probe-type categories (Type-I M red, Type-I U red, Type-I M green,

Type-I U green, Type-II red, and Type-II green). Beta values were then calculated from these normalised intensity values

Covariates
Covariates were collected via questionnaires collected at recruitment, before and during pregnancy.

Maternal age was derived from mothers' date of birth. Child's gender and gestational age at birth were collected from stored clinical information. In case of maternal BMI, we used BMI at 1st trimester. Maternal smoking status and parity were collected

from the responses from the questionnaires. Maternal smoking status in pregnancy (Yes/No) was defined as any smoking in pregnancy or no smoking in pregnancy. Socioeconomic status was defined using maternal socioeconomic cluster information (high, low, low-low, low-mid, and mid).

Cell type correction
We used the Reinius-based Houseman method*(5, 6)* with the estimateCellCounts function in the Minfi package*(7)* to estimate relative proportions of six white blood cell subtypes (CD4+ T-lymphocytes, CD8+ T-lymphocytes, NK (natural killer) cells, B-lymphocytes, monocytes and granulocytes).

Batch correction
Indicator of different batches that DNA methylation data were generated and ComBat*(16)* was used to adjust for batch effect.

Exclusion criteria
In the methylation subset of the samples were excluded infants born preterm (<37 weeks), and whose mothers experienced pre-eclampsia or diabetes during pregnancy. This information was obtained from questionnaires completed by the mothers shortly after delivery and combined with medical birth records.

Isle of Wight Third Generation (IOW F2)
Design and study population
The recruitment of newborns started from April 2010. Data used in the analyses were from infants born between April 2010 to May 2014*(58)*. In total, 200 newborns were recruited such that at least one of their parents is in the IOW birth cohort (IOW F1) and the recruitment is ongoing. For each infant, along with other phenotypic information such as gender and birthweight, status of wheezing and eczema was recorded, measures of wheal size from skin prick test as well as IgE were recorded.

Birthweight
Newborn birthweight was collected from stored clinical records. The outliers for observe birthweight values +/- 5 SD from the mean was checked. For the main analysis model birthweights were treated as continuous variables in grams and for the dichotomous model birthweights were categorised in high (>4000g) versus normal (2500-4000g) birthweight.

Methylation measurements
For 118 subjects, we measured DNA methylation from whole blood processed with the Illumina Infinium HumanMethylation450 BeadChip (Illumina Inc., San Diego, USA). CPACOR*(59)* pipeline was used for QC and normalisation of the data. Methylation markers on 65 single nucleotide polymorphism (SNP) and sex chromosomes were removed. We applied Illumina background

Correction to all intensity values. Any intensity values having detection p-values >= 10-16 were set as missing data. Samples with call rate < 98% were excluded. After the QC, 389,828sites remain for the subsequent analysis. A quantile normalisation was applied using limma on intensity values

separately based on six different probe-type categories (Type-I M red, Type-I U red, Type-I M green,

Type-I U green, Type-II red, and Type-II green). Beta values were then calculated from these normalised intensity values

Covariates
Covariates were collected via questionnaires collected at recruitment, before and during pregnancy.

Maternal age was derived from mothers' date of birth. Child's gender and gestational age at birth were collected from stored clinical information. In case of maternal BMI, we used BMI at 1st trimester. Maternal smoking status and parity were collected from the responses from the questionnaires. Maternal smoking status in pregnancy (Yes/No) was defined as any smoking in pregnancy or no smoking in pregnancy. Socioeconomic status was defined using maternal education: 1) left before general certificate of secondary education, 2) completed education at 16 years, 3) completed education at 18 years, and 4) Other, e.g., vocational training. Subjects who completed education at 16 years or above were included into group 1.

Cell type correction
We used the Reinius-based Houseman method*(5, 6)* with the estimateCellCounts function in the Minfi package*(7)* to estimate relative proportions of six white blood cell subtypes (CD4+ T-lymphocytes, CD8+ T-lymphocytes, NK (natural killer) cells, B-lymphocytes, monocytes and granulocytes).

Batch correction
Indicator of different batches that DNA methylation data were generated and ComBat*(16)* was used to adjust for batch effect.

Exclusion criteria
In the methylation subset of the samples were excluded infants born preterm (<37 weeks), and whose mothers experienced pre-eclampsia or diabetes during pregnancy. This information was obtained from questionnaires completed by the mothers shortly after delivery and combined with medical birth records.

LifeLines Cohort study
Design and study population
The LifeLines Cohort Study is a multi-disciplinary prospective large population-based cohort study examining in a unique three-generation design the health and health-related behaviors of 167,729 persons living in the North of the Netherlands. It employs a broad range of investigative procedures in assessing the biomedical, socio-demographic, behavioral, physical and psychological factors which contribute to the health and disease of the general population, with a special focus on multi-morbidity and complex genetics. In addition, a biobank was established as a resource for research on complex interactions between environmental, phenotypic and genomic factors in the development of chronic diseases and healthy ageing*(60, 61)*. Between 2006 and 2013, inhabitants of the northern part of The Netherlands and their families were invited to participate, thereby contributing to a three-generation design. Participants visited one of the LifeLines research sites for a physical examination and completed extensive questionnaires. Baseline data were collected for 167,729 participants, aged from 6 months to 93 years. Follow-up visits are scheduled every 5 years, and in between participants receive follow-up questionnaires. This study has been approved by the Medical Ethical Committee of the University Medical Center Groningen and all participants gave written informed consent.

Birthweight
Birthweight was measured by questionnaire. All subjects with a self-reported birthweight between 950 and 5000 grams were included. For the main analysis model we treated birthweight as a continuous trait in grams and for the dichotomous model we categorised birthweight in high (>4000g) versus normal (2500-4000g) birthweight. We had complete data (including all covariates and methylation data) on 745 and 728 subjects for the continuous and dichotomous analyses respectively.

Methylation measurements
The methylation levels at the baseline assessment of LifeLines were determined for use in a study on environmental exposures, DNA methylation, and respiratory health. In total, 1,656 subjects were selected based on having complete data on sex, age, height, smoking history (pack-years, never- or current-smoker), airway obstruction (FEV1/FVC<70%), and occupational related exposures. The Illumina Infinium Human Methylation 450K arrays were used to define genome-wide DNA methylation levels in whole blood at >450.000 specific CpGs. We randomized 1,656 subjects based on sex, exposure, and airway obstruction over the chips. Using 500 ng DNA for each sample, we first performed a bisulphite conversion using the EZ- 96 DNA methylation kit (Zymo research Corporation, Irvine, USA), which was validated using commercially available bisulphite conversion 1 control samples (Zymo Research Corporation, Irvine, USA). After this step the samples were processed according to the Illumina 450K protocol (Illumina Inc., San Diago, USA). Quality-control (QC) steps were performed using the Minfi package in R*(29)*, and included the removal of samples with >1% of all probes having a detection p-value >0.01, and samples with an incorrect sex or SNP prediction. We removed single probes with a detection p-value >0.01, sex chromosome probes, cross-reactive probes, probes measuring SNPs, and probes where the CpG itself or the single base extension (SBE) site is a SNP*(13)*. The data were normalized using DASEN implemented in the wateRmelon package in R*(45)*. The final data set contained data for 1,622 subjects and 420,938 CpG probes. Beta-values were used to represent DNA methylation levels, which is the ratio between the intensities of methylated versus unmethylated probes, ranging from 0 to 1.

Covariates
Data on age (at the time of blood collection), gender, gestational age, maternal smoking, maternal age at delivery, parity, current social class (defined by educational level), and current smoking were determined by questionnaires. Current BMI was measured during the baseline visit. Data on maternal social class and pre-pregnancy BMI were not available. All subjects were Dutch Caucasians, so the analysis was not adjusted for etnicity.

Cell type correction
The minfi package was used to calculate the cell proportions using the Houseman method*(6)*. These include: CD8T, CD4T, NK, Bcell, Mono and Gran.

Batch correction
To estimate possible batch effects, a principal component (PC) analysis was performed using the control probes included on the 450K chip*(59)*. We included seven PCs in the final model that explained >1% of the variance, and captured in total >95% of the technical variance.

Exclusion criteria
In the methylation subset of the LifeLines cohort we excluded infants born preterm (<37 weeks).

Norwegian Mother and Child Cohort Study (MoBa1, MoBa2 and MoBa3)
Design and study population
Participants represent three subsets of mother-offspring pairs from the national Norwegian Mother and Child Cohort Study (MoBa)*(62)*. MoBa is a prospective population-based pregnancy cohort study conducted by the Norwegian Institute of Public Health. The years of birth for MoBa participants ranged from 1999-2009. MoBa mothers provided written informed consent. Each

subset is referred to here as MoBa1, MoBa2, and MoBa3. MoBa1 is a subset of a larger study within MoBa that included a cohort random sample and cases of asthma at age three years*(63)*. We previously reported an association between maternal smoking during pregnancy and differential DNA methylation in MoBa1 newborns*(64)*. We subsequently measured DNA methylation in additional newborns (MoBa2) in the same laboratory (Illumina, San Diego, CA)*(65)*. MoBa2 included cohort random samples plus cases of asthma at age seven years and non-asthmatic controls. MoBa3 was designed to evaluate the association between differential cord blood DNA methylation and later childhood cancer status. Methylation measurements for MoBa3 were made at IARC. Years of birth were 2002-2004 for children in MoBa1, 2000-2005 for MoBa2, and 200-2008 for MoBa3. The establishment and data collection in MoBa has obtained a license from the Norwegian Data Inspectorate and approval from The Regional Committee for Medical Research Ethics. All three studies were approved by the Regional Committee for Ethics in Medical Research, Norway. In addition, MoBa1 and MoBa2 were approved by the Institutional Review Board of the National Institute of Environmental Health Sciences, USA.

Birthweight
In MoBa, birthweight was collected in the Medical Birth Registry of Norway (MBRN). The distribution was checked for values outside +/-5SD for removal (0 in MoBa1, MoBa2 and MoBa3). Both the continuous and the dichotomous high (>4000g) versus normal (2500-4000g) birthweight models were analyzed in MoBa1, MoBa2, and MoBa3.

Methylation measurements
Details of the DNA methylation measurements and quality control for the MoBa1 participants were previously described*(66)* and the same protocol was implemented for the MoBa2 participants. Briefly, umbilical cord blood samples were collected and frozen at birth at -80°C. All biological material was obtained from the Biobank of the MoBa study*(66)*. Bisulfite conversion was performed using the EZ-96 DNA Methylation kit (Zymo Research Corporation, Irvine, CA) and DNA methylation was measured at 485,577 CpGs in cord blood using Illumina's Infinium HumanMethylation450 BeadChip*(67)*. Raw intensity (.idat) files were handled in R using the minfi package to calculate the methylation level at each CpG as the beta-value (β=intensity of the methylated allele (M)/(intensity of the unmethylated allele (U) + intensity of the methylated allele (M) + 100)) and the data was exported for quality control and processing. Probe and sample-specific quality control was performed in the MoBa1, MoBa2, and MoBa3 datasets separately. Similar protocols were applied to MoBa1 and Moba2, as follows: Control probes (N=65) and probes on X (N=11 230) and Y (N=416) chromosomes were excluded in both datasets. Remaining CpGs missing > 10% of methylation data were also removed (N=20 in MoBa1, none in MoBa2). Samples indicated by Illumina to have failed or have an average detection p value across all probes < 0.05 (N=49 MoBa1, N=35 MoBa2) and samples with gender mismatch (N=13 MoBa1, N=8 MoBa2) were also removed. For MoBa1 and MoBa2, we accounted for the two different probe designs by applying the intra-array normalization strategy Beta Mixture Quantile dilation (BMIQ)*(19)*. After quality control exclusions, the sample sizes were 1,068 for MoBa1 and 685 for MoBa2. For MoBa3, samples were completely randomized, and bisulfite conversion and methylation measurements were performed by the Epigenetics Group at IARC (Lyon, France). Similar data quality control and processing was applied with some slight differences. Methylation features were filtered from (i) cross-reactive probes, (ii) probes mapping to sex chromosomes and (iii) probes overlapping with a known single nucleotide polymorphism (SNP) with an allele frequency of at least 5% in the overall population (all ethnic groups), resulting in the exclusion of 36 231 probes. Data quality was further assessed using box plots for the distribution of methylated and unmethylated signals, and multidimensional scaling plots and unsupervised clustering were used to check for sample outliers. After background correction and color-bias adjustment, type I and type II probe distributions were aligned using the intra-sample BMIQ normalization*(19)* from the watermelon package. After quality control, the sample size for MoBa3 was 253.

Covariates
For all three datasets, information on gestational age, child's sex, maternal age, smoking during pregnancy, education, pre-pregnancy BMI, and parity was collected via questionnaires completed by the mother or from birth registry records as previously described (4). Gestational age, maternal age, and pre-pregnancy BMI were included as continuous variables. Child's sex and parity were included as dichotomous variables. Maternal smoking status during pregnancy was classified into three groups: non-smoker, stopped smoking in early pregnancy, and smoked throughout pregnancy. Maternal educational level was categorized into four groups based on years of education: less than high school/secondary school, high school/secondary school completion, some college or university, or 4 years of college/university or more.

The current analyses include the children who had cord blood DNA methylation measurements that passed quality control, birthweight and covariate data (N=1025 from MoBa1; N=642 from MoBa2; N=205 from MoBa3). Each dataset was analysed independently.

Cell type correction
We used the Houseman method*(6)* with the estimateCellCounts function in the Minfi package*(7)* in R*(8)* to estimate relative proportions of six white blood cell subtypes (CD4+ T-lymphocytes, CD8+ T-lymphocytes, NK (natural killer) cells, B-lymphocytes, monocytes and granulocytes).

Batch correction

The Empirical Bayes method via ComBat was applied separately in MoBa1, MoBa2 and MoBa3 for batch correction using the sva package in R*(57)*.

Exclusion criteria
For the main analysis we excluded infants born preterm (<37 weeks; N=37 for MoBa1, N=30 for MoBa2, and N=0 for MoBa3), and whose mothers experienced pre-eclampsia (N=34 for MoBa1, N=26 for MoBa2, and N=0 for MoBa3) or diabetes (N=18 for MoBa1, N=2 for MoBa2, and N=0 for MoBa3) during pregnancy. This information was collected via questionnaires completed by the mother or from birth registry records as previously described*(62, 68)*.

Norway Facial Clefts Study (NCL)
Design and study population
The Norway Facial Clefts Study is a national population-based case-control study of cleft lip and cleft palate, disorders characterized by the incomplete fusion of the lip and/or palate during development. The study design has been previously described in detail*(69)*. Study approval was obtained by the Norwegian Data Inspectorate and Regional Medical Ethics Committee of Western Norway and informed consent was provided by both the mother and father. Briefly, between the years of 1996 and 2001 all families of newborns referred for cleft surgery in Norway were contacted and, of those eligible, 88% agreed to participate (N=573). Controls were selected by a random sampling of roughly 4 per 1000 live births in Norway during that same time period and, of those eligible, 76% agreed to participate (N=763). After completion of data collection and linkage with the Medical Birth Registry, all identifiers were permanently stripped from the data set, with no opportunity for further follow-up.

Birthweight
Newborn birthweight was recorded by obstetricians or midwives in the delivery room or abstracted from obstetric records. We double checked and did not observe birthweight values +/- 5 SD from the mean.

Methylation measurements
Epigenome-wide DNA methylation was measured in 898 newborns, using DNA extracted from heel stick blood samples that were collected 2-3 days after delivery as part of a standardized program of testing for phenylketonuria (PKU). One microgram of DNA was bisulfite converted using the EZ DNA Methylation kit following the manufacturer's protocol. 898 newborn and 60 technical control samples were run on Illumina HumanMethylation450 BeadChips according to the manufacturer's instructions at the NIH Center for Inherited Disease Research. 22 samples having percentage of low quality data points (undetectable (detection $p > 0.05$) CpG or number of beads < 3) greater than 1% were excluded; 6 samples were excluded due to missing data for the covariates in the analysis models as required for the complete data analysis; total of 68 samples were excluded due to: 51 preterm births (gestational week <37), 11 non-singletons and 6 samples were both preterm birth and non-singleton. After exclusions, 802 samples remained for analysis.

Raw methylation intensity data were first background corrected using ENmix model*(70)*, and then Methylated (M) or Unmethylated (U) intensities were separately quantile normalized across samples for Infinium I or II probes. The β-value (M/(M+U+100)) was then adjusted for probe type bias using BMIQ method*(19)*.

Covariates
Information on maternal age, parity, and maternal education was collected in the same self-administered questionnaires (around 3-4 months after delivery). Facial cleft status was categorized as none (control), cleft lip with or without cleft palate, and cleft palate only. Analyses were additionally adjusted for a categorical variable kids birth year.

Cell type correction
We used the Houseman method*(6)* with the estimateCellCounts function in the Minfi package*(7)* in R*(8)* to estimate relative proportions of six white blood cell subtypes (CD4+ T-lymphocytes, CD8+ T-lymphocytes, NK (natural killer) cells, B-lymphocytes, monocytes and granulocytes).

Batch correction
We additionally corrected the analyses for batch effect by including bisulfite plate variable.

Exclusion criteria
For the main analysis we excluded n=68 participants; 57 infants born preterm (<37 weeks), and non-singleton infants (n=11). This information was obtained from questionnaires completed by the mothers shortly after delivery and combined with medical birth records.

The Newborn Epigenetics Study (NEST)
Design and study population
The Newborn Epigenetics STudy (NEST) is a multiethnic birth cohort designed to identify the effects of early exposures on epigenetic profiles and phenotypic outcomes. Pregnant women were recruited from prenatal clinics serving Duke University

Hospital and Durham Regional Hospital Obstetrics facilities in Durham, North Carolina from April 2005 to July 2009. Gestational age at enrollment ranged from 6 to 42 weeks (median 30 weeks). Eligibility criteria were women aged 18 years or older, English speaking, pregnant, and an intention to use one of the two obstetrics facilities. Among these, women infected with HIV or intending to give up custody of the offspring of index pregnancy were excluded. Current smokers were targeted for the first ~200 participants. Of the 1101 women who met eligibility criteria and were approached, 895 (81%) were enrolled and umbilical cord blood was collected from 741 infants. The current analysis was limited to the 413 infants with 450k and covariate data. This study was approved by the Duke Institutional Review Board. Additional details about NEST may be found in previous publications*(71, 72)*.

Birthweight
Birthweight was recorded by the clinician responsible for the delivery of the baby and abstracted from the medical records by the research team. Values +/- 5 SD from the mean were not found in the data.

Methylation measurements
Genomic DNA from buffy coat specimens was extracted from umbilical cord blood using Puregene Reagents (Qiagen, Valencia, CA). Bisulfite conversion was performed using the EZ-96 DNA Methylation Kit (Zymo Research Corporation) and DNA methylation was measured at 485,577 CpGs using Illumina Infinium HumanMethylation450 BeadChip (Illumina Inc., San Diego, USA). Illumina's GenomeStudio Methylation module version 1.0 (Illumina Inc.) was used to calculate the methylation level at each CpG as the beta value. Probe and sample-specific quality control was performed in the NEST cohort using a similar approach to MoBa1 and MoBa2 cohorts. Specifically, control probes (N=65) and probes on X (N=11 230) and Y (N=416) chromosomes were excluded as well as CpGs missing > 10% of methylation data. Samples indicated by Illumina to have failed or have an average detection P-value across all probes < 0.05 and samples with gender mismatch were also removed. The two different probe designs by applying the intra-array normalization strategy Beta Mixture Quantile dilation (BMIQ)*(19)*.

Covariates
The gestational age and gender of the child were collected from medical records following delivery. Maternal smoking status, socioeconomic status (education), age, prepregnancy body mass index (BMI), parity, and race were reported by the mother on a questionnaire completed during pregnancy. Models were stratified by race/ethnicity group (Caucasian or African American).

Cell type correction
The Reinius-based Houseman method*(5, 6)* was used with the estimateCellCounts function in the Minfi package*(7)* in R*(8)* to estimate relative proportions of six white blood cell subtypes (CD4+ T-lymphocytes, CD8+ T-lymphocytes, NK (natural killer) cells, B-lymphocytes, monocytes and granulocytes).

Batch correction
The Empirical Bayes method via ComBat was applied for batch correction using the sva package in R*(16)*.

Exclusion criteria
For the main analysis we excluded n=217 participants: 90 infants born preterm (<37 weeks), non-singleton births (n=3), missing covariates (n=54) and whose mothers experienced pre-eclampsia (n=24) or diabetes (n=29) during pregnancy or were a race other than Caucasian or African-American (n = 17). This information was obtained from questionnaires completed by the mothers shortly after delivery and combined with medical birth records.

The Northern Finland Birth Cohorts (NFBC1966 and NFBC1986)
Design and study population
NFBC1966: The Northern Finland Birth Cohort 1966 is a prospective follow-up study of children from the two northernmost provinces of Finland*(73)*. 96% of all woman in this region with expected delivery dates in 1966 were recruited though maternity health centres (12,058 live births). All individuals still living in northern Finland or the Helsinki area (n = 8,463) were contacted and invited for clinical examination. A total of 6007 participants attended the clinical examination at the participants' age of 31 years. DNA was extracted from blood samples given at the clinical examination (5,753 samples available)*(74)*.The subset with DNA is representative of the original cohort in terms the major environmental and social factors known to influence the tested trait. An informed consent for the use of the data including DNA was obtained from all subjects. DNA methylation was measured for 807 randomly selected subjects that attended the clinical examination and completed the questionnaire For DNA methylation marker calling we used a detection P-values threshold of <10-16 A call rate filter of 95% was applied to the all autosomal Illumina probes yielding 459378 probes for association testing. 67 samples were excluded due to low marker call rate (<95%). 7 samples were excluded for gender inconsistency, one sample for globally outlying DNA methylation values (1st PC score of the DNA methylation values outside mean +/- 4SD).

NFBC1986: The Northern Finland Birth Cohort 1986 consists of 99% of all children, who were born in the provinces of Oulu and Lapland in Northern Finland between 1 July 1985 and 30 June 1986. 9,203 live-born individuals entered the study*(75)*. At the age of 16, the subjects living in the original target area or in the capital area (n=9,215) were invited to participate in a follow-up study

including a clinical examination. 7344 participants attend the study in year 2001/2002, of which 5654 completed the postal questionnaire, the clinical examination and provided a blood sample*(76)*. DNA was extracted from all 5654 blood samples. An informed consent for the use of the data including DNA was obtained from all subjects. DNA methylation was recoded on Illumina HumanMethlation450K array for 566 randomly selected subjects. 24 technical replicates were excluded. 18 samples did not reach a call rate of >95% applying a detection P-value filter of 10^{-16}. We excluded 7 samples with gender inconsistency, no sample was outlying from the overall data structure (1st PC score of the DNA methylation values outside mean +/- 4SD). DNA methylation data of 517 samples with 466290 autosomal probes (call rate filter 95%) each were used for this analysis.

Birthweight
New-born birthweight was recorded by obstetricians or midwives. We did not observe birthweight values +/- 5 SD from the mean.

Methylation measurements
Methylation of genomic DNA was quantified using the Illumina HumanMethylation450 array according to manufacturer's instructions. Bisulfite conversion of genomic DNA was performed using the EZ DNA methylation kit according to manufacturer's instructions (Zymo Research, Orange, CA).

Covariates
Data on maternal parity, socio-economic status, smoking, pre-pregnancy body mass index, age at birth, newborn gender and gestational age at birth were self-reported in questionnaires during pregnancy.

Cell type correction
Potential confounding effects of blood cell subtypes were estimated by the Houseman method*(6)*.

Batch correction
To account for batch effects in the data, beta values underwent a functional normalization approach described by Fortin *et al.(18)* using the first 10 PCs of the Illumina 450K array control probes. This approach includes subset quantile normalization of the data and normal-exponential out-of-band background correction.

Exclusion criteria
We excluded preterm born children (n=24 in NFBC66; n=12 in NFBC86) and children of mothers diagnosed with gestational diabetes cases (n=2 in NFBC66; n=4 in NFBC86) from the analysis.

New Hampshire Birth Cohort Study (NHBCS)

Design and study population
The New Hampshire Birth Cohort Study (NHBCS), which began in 2009, is an ongoing prospective study of over 1500 women receiving prenatal care in New Hampshire, USA*(77)*. Pregnant mothers were recruited between 24-28 weeks gestation. Eligibility criteria included literacy in English, between 18–45 years of age, reported use of a private well as the primary source of home drinking water, and singleton pregnancies. All participants provided written informed consent in accordance with the requirements of the Institutional Review Board (IRB) at Dartmouth College. Pre- and post-delivery questionnaires were administered to collect self-reported sociodemographic, lifestyle, and medical history data, and a structured medical records review was employed to collect information about the pregnancy and delivery. Cord blood samples are collected on ~80% of eligible deliveries.

Birthweight
Participants also consented to a medical record review, which allowed additional information to be recorded about prenatal infections, medication use, birth outcomes and delivery details, and general health of the women and their infants after birth.

Methylation measurements
This study consisted of the first participants born in the study with available cord blood samples for DNA methylation analysis, complete information for all covariates (n=96). DNA was bisulfite converted using the EZ DNA Methylation kit and subsequently subjected to epigenome-wide DNA methylation assessment using the Illumina Infinium HumanMethylation450 BeadChip at the University of Minnesota Genomics Core Facility following standardized protocols. Post-array processing was conducted in the 'minfi' package in R. Array control probes were used to assess the quality of our samples and evaluate potential poor bisulfite conversion or color-specific issues for each array. Probes with detection p-values > 0.01 in at least one sample were removed. Data was then normalized using functional normalization (funNorm).

Covariates
Self-reported smoking during pregnancy (any smoking during pregnancy vs. no smoking during pregnancy), maternal highest educational attainment (high school graduation or less vs. at least some post high school education), parity (ever previously gave birth vs. never previously gave birth), and maternal age (continuous) were collected via questionnaire. Infant sex and gestational age were collected via medical records abstraction.

Cell type correction

Proportions of cell types were estimated from the 450K DNAM via the estimateCellCounts function*(5, 6)* within the 'minfi' package in R. Due to the sum of the six estimated cell proportions adding to 1.0 for each participant, only five of the cell-types were added to the models as covariates (granulocytes were excluded).

Batch correction
Batch effects were removed from the methylation data via ComBat; removal of batch effects was confirmed with principal components analysis.

Exclusion criteria
For the main analysis we excluded n=19 participants: 7 infants born preterm (<37 weeks), and whose mothers experienced pre-eclampsia (n=1) or diabetes (n=11) during pregnancy. This information was obtained from medical birth records.

Netherlands Twin Register (NTR)
Design and study population
The subjects were twins who participated in longitudinal survey studies from the Netherlands Twin Register (NTR)*(78)* and participated in the NTR biobank project between 2004 and 2011*(79)*. The NTR is a longitudinal twin-family study with no other selection criteria than being a multiple or one of their family members. Longitudinal surveys have been described in detail previously*(78)*. Complete information (DNA methylation, birthweight and all covariates from model 9) were available for 175 samples from 168 individuals who met the inclusion criteria. For 7 individuals, longitudinal methylation data (two samples) were included in the EWAS. Informed consent was obtained from all participants. The study was approved by the Central Ethics Committee on Research Involving Human Subjects of the VU University Medical Centre, Amsterdam, an Institutional Review Board certified by the U.S. Office of Human Research Protections (IRB number IRB00002991 under Federal-wide Assurance-FWA00017598; IRB/institute codes, NTR 03-180).

Birthweight
Data on birthweight were obtained from self-report by the twins themselves or their parents. Data collected across multiple surveys and projects were combined and consistency across family members and time was checked. When multiple data points differed by less than 200 grams, the average was taken, and in the cases of larger differences, data were excluded. In accordance to the PACE analysis plan, individuals with a birthweight lower than 2500 grams were excluded. After this exclusion, birthweight values > 5SD above the mean or lower than 5 SD below the mean did not occur in this dataset. Only the continuous birthweight analyses were performed. The dichotomous analysis (high (>4000g) versus normal (2500-4000g)) was not performed because few very twins had a birthweight greater than 4000g.

Methylation measurements
Blood sampling procedures have been described in detail previously*(79)*. DNA methylation was assessed with the Infinium HumanMethylation450 BeadChip Kit (Illumina, San Diego, CA, USA Illumina, San Diego, CA, USA) by the Human Genotyping facility (HugeF) of ErasmusMC, the Netherlands (http://www.glimdna.org/) as part of the Biobank-based Integrative Omics Study (BIOS) consortium*(80)*. DNA methylation measurements have been described previously*(80, 81)*. Genomic DNA (500ng) from whole blood was bisulfite treated using the Zymo EZ DNA Methylation kit (Zymo Research Corp, Irvine, CA, USA), and 4 µl of bisulfite-converted DNA was measured on the Illumina 450k array following the manufacturer's protocol. A number of sample- and probe-level quality checks and sample identity checks were performed (described in detail previously*(81)*). In short, sample-level QC was performed using MethylAid*(82)*. Probes were set to missing in a sample if they had an intensity value of exactly zero, or a detection *p*>.01, or a bead count of<3. After these steps, probes that failed based on the above criteria in >5% of the samples were excluded from all samples (only probes with a success rate≥ 0.95 were retained). Probes were also excluded from all samples if they mapped to multiple locations in the genome*(18)*. The methylation data were normalized with functional normalization*(6)* and normalized intensity values were converted into beta (β)-values. In accordance to the PACE analysis plan, the methylation dataset was trimmed on: (25th percentile -3*IQR) and (75th percentile+3*IQR). In total, 453288 sites were analysed.

Covariates
The following covariates were included in model 9: gestational age, sex, maternal educational attainment, maternal smoking during pregnancy, maternal age, maternal BMI, older siblings (yes/no), array row number, 3 PCs based on genome-wide methylation data, Bcell, CD4T, CD8T, Gran, Mono, NK. The following covariates were included in model 11: gestational age, sex, maternal educational attainment, maternal smoking during pregnancy, maternal age, maternal BMI, older siblings (yes/no), array row number, 3 PCs based on genome-wide methylation data, Bcell, CD4T, CD8T, Gran, Mono, NK, age, BMI, smoking, educational attainment person. Information on BMI and smoking status were collected as part of the NTR biobank project at the moment of blood draw*(79)*. Data on gestational age, maternal age at the time of delivery, and maternal pre-pregnancy BMI were obtained retrospectively from self-report of the mother. Information on whether the twins had older siblings was obtained through self-report by the twins. Data on educational attainment and maternal educational attainment were obtained from self-report by the

twins themselves or their parents. Data on educational attainment that were collected across multiple surveys were combined and consistency across family members and time was checked. Educational attainment was defined as the highest completed level of education at the age of 25 or higher. We analysed information on maternal smoking during pregnancy that was obtained in NTR Survey 10 (data collection in 2013) with the following question: Did your mother ever smoke during pregnancy? with answer categories: no, yes, I don't know. For twin pairs, the answers were checked for consistency and missing data for one twin were supplemented with data from the co-twin where possible: If one twin answered "yes" or "no", and his/her co-twin did not fill out the survey, or did not answer this question, or answered "I don't know", the missing information was replaced by the information supplied by the co-twin. If one twin answered "yes" and the co-twin answered "no" (3.6% of twin pairs for which an answer was available for both twins), data from both twins were set to missing. Ethnicities was defined using genetic data, which was checked for consistency with self or maternal reports.

Cell type correction
The minfi package was used to calculate the cell proportions using the Houseman method*(6)*. These include: CD8T, CD4T, NK, Bcell, Mono and Gran.

Batch correction
Illumina 450k array row and 3 PCs based on the genome-wide methylation data were included as covariates to account for technical variability between samples, as these PCs were correlated with several indices related to the lab procedure, such as sample plate and order of processing, as described previously.

Exclusion criteria
In accordance to the PACE analysis plan, we excluded preterm births (i.e. ≥37 weeks only) and individuals with a birthweight lower than 2500 grams. Information on maternal diabetes and pre-eclampsia were not available for these subjects.

Prevention and Incidence of Asthma and Mite Allergy (PIAMA)
Design and study population
The PIAMA study is a birth cohort study of children born between 1996-1997. Details of the study design have been published previously*(83)*. In brief, pregnant women were recruited during their first trimester from the general population in 1996-1997 through antenatal clinics in the north, west and center of the Netherlands. Non-allergic pregnant women were invited to participate in a ''natural history'' study arm. Pregnant women identified as allergic through the screening questionnaire were allocated primarily to an intervention arm with a random subset allocated to the natural history arm. The intervention involved the use of mite-impermeable mattress and pillow covers. The study started with 3,963 newborns. Parents completed questionnaires on demographic factors, risk factors for asthma and respiratory symptoms at the child's age of 3 months, annually from 1 to 8 years of age, and at 11, 14, and 16 years of age. Clinical examinations were performed in subgroups at ages 4, 8, 12 and 16 years.

Birthweight
Parents were asked to report their child's birthweight, as stated in the delivery report, in the 3- months questionnaire.

Methylation measurements
In the PIAMA study, DNA from peripheral blood samples was extracted using the QIAamp blood kit (Qiagen or equivalent protocols), followed by precipitation-based concentration using GlycoBlue (Ambion). DNA concentration was determined by Nanodrop measurement and Picogreen quantification. 500 ng of DNA was bisulphite-converted using the EZ 96-DNA methylation kit (Zymo Research), following the manufacturer's standard protocol. After verification of the bisulphite conversion step using Sanger Sequencing, DNA concentration was normalized and the samples were randomized to avoid batch effects. The DNA methylation was measured using the Illumina Infinium HumanMethylation450 beadchip. Each chip included one control DNA sample for quality control purposes. Data preprocessing was performed using the Minfi package*(29)*. We implemented sample filtering to remove bad quality samples (call rate <99%). Moreover, we used 65 SNP probes to check for concordances between paired DNA samples. Paired samples from the same individuals which show Pearson correlation coefficient <0.9 were regarded as sample mixed ups and were excluded from the study. We further assessed the methylation distribution of the X-chromosome to verify gender. After QC, 207 good quality 8 years and 640 good quality 16 years PIAMA samples were used in the analysis. During processing, the probes on sex chromosomes, the probes that mapped to multiple loci, 65 SNP-probes and the probes containing SNPs at the target CpGs with a MAF>5% were excluded*(13)*. We implemented "DASEN"*(45)* to perform signal correction and normalization.

Covariates
Maternal age was defined as a continuous variable. Maternal social class was defined as the highest attained educational level and coded in three categories: 1=primary school, lower vocational or lower secondary education (low) 2=intermediate vocational education or intermediate/higher secondary education (intermediate) 3= higher vocational education and university (high). Parity was defined as older siblings living in the PIAMA home. Pre-pregnancy maternal BMI was calculated using height and weight of

the mother before pregnancy, self-reported in the questionnaire when the child was 1 year of age. Maternal smoking during pregnancy was coded as 1= no smoking in pregnancy, 2=smoking but stopped in the first 16 weeks, 3=smoking for longer than 16 weeks. The current status of asthma was also added as co-variable since the samples were enriched with asthma cases.

Cell type correction
Cell type correction was applied using the reference-based Houseman method*(6)* in the Minfi package*(29)*. This method estimates the relative proportions of six white blood cell subtypes (CD4+ T-lymphocytes, CD8+ T-lymphocytes, NK (natural killer) cells, B-lymphocytes, monocytes and granulocytes).

Batch correction
In age 8 models, bisulfite conversion kits (2 batches) have been used as batch variables. In age 16 models, the samples were run into two batches, and therefore batch was included in the model.

Exclusion criteria
For the main analysis of age 8, we excluded n=10 participants due to low birthweight (<2, 500g). For the main analysis of age 16, we excluded 19 participants due to low birthweight (<2,500g).

Prediction and Prevention of Preeclampsia and Intrauterine Growth Restriction study (PREDO)
Design and study population
Data were from the Prediction and Prevention of Preeclampsia and Intrauterine Growth Restriction (PREDO) Study, which is a longitudinal multicenter pregnancy cohort study of Finnish women and their singleton children born alive between 2006-2010*(84)*. We recruited 1079 pregnant women, of whom 969 had one or more and 110 had none of the known risk factors for preeclampsia and intrauterine growth restriction. The recruitment took place in arrival order when these women attended the first ultrasound screening at 12+0-13+6 weeks+days of gestation in one of the ten hospital maternity clinics participating in the study. The cohort profile*(84)* contains details of the study design and inclusion criteria. The study protocol was approved by the Ethical Committees of the Helsinki and Uusimaa Hospital District and by the participating hospitals. A written informed consent was obtained from all women. The study has been registered as ClinicalTrials.gov identifier ISRCTN14030412.

Birthweight
Newborn birthweight was measured by obstetricians or midwives in the delivery room and abstracted from birth records. We double checked and did not observe birthweight values +/- 5 SD from the mean.

Methylation measurements
Cord blood samples were ran on Illumina 450K Methylation arrays. To limit batch effects, we randomized all samples over the 96-well plates, based on gender and maternal risk factors for pre-eclampsia. Samples were placed on 96-well plates. Bisulfite conversion was performed using the EZ-96 DNA methylation kit (Zymo research Corporation, Irvine, USA). Then we used the Infinium HumanMethylation450 BeadChip (Illumina Inc., San Diego, USA) to measure the methylation level as a beta value ranging from 0 (no methylation) to 1 (complete methylation).The quality control pipeline was set up using the R-package minfi*(8, 29)*. Three IDs were excluded as they were outliers in the median intensities. Furthermore, 20 IDs showed disconcordance between phenotypic sex and estimated sex and were excluded. Nine IDs were contaminated with maternal DNA (Morin et al, submitted) and were also removed. Methylation beta-values were normalized using the funnorm function. We excluded any probes on chromosome X or Y, probes containing SNPs and cross-hybridizing probes according to Chen *et al.(13)* and Price *et al.(49)* Furthermore, any CgGs with a detection p-value > 0.01 in at least 25% of the samples were excluded.

Covariates
Data on maternal parity, socio-economic status and smoking during pregnancy, pre-pregnancy body mass index, age at childbirth, newborn sex and gestational age at birth were extracted from the Finnish Medical Birth Register.

Cell type correction
We used the method by Houseman*(6)* with the estimateCellCounts function in the Minfi package in R to estimate relative proportions of six white blood cell subtypes (CD4+ T-lymphocytes, CD8+ T-lymphocytes, NK (natural killer) cells, B-lymphocytes, monocytes and granulocytes).

Batch correction
After normalization two batches, i.e. slide and well, were significantly associated and were removed iteratively using the Combat method*(16)*.

Exclusion criteria
For the main analysis we excluded n=254 participants: 33 infants born preterm (<37 weeks), and whose mothers experienced pre-eclampsia (n=62) or diabetes (n=192) during pregnancy. This information was obtained from questionnaires completed by the mothers shortly after delivery and combined with medical birth records.

Programming of Intergenerational Stress Mechanisms (PRISM)

Design and study population

The PRogramming of Intergenerational Stress Mechanisms (PRISM) study, is a prospective pregnancy cohort of mother-child pairs originally designed to examine how perinatal stress influences respiratory health in children. Women were recruited from prenatal clinics during the first or second trimester (<28 weeks gestation) from the Beth Israel Deaconess Medical Center (BIDMC) and the East Boston Neighborhood Health Center in Boston Massachusetts, USA, from March 2011 to August 2012 and from the Icahn School of Medicine at Mount Sinai in New York, New York, USA, starting in April 2013 where collections are still underway. Recruitment sites were chosen to ensure desired heterogeneity in sociodemographic and racial/ethnic characteristics. Eligibility criteria included: (i) English- or Spanish-speaking; (ii) age ≥18 years at enrollment; and (iii) singleton pregnancy. Procedures were approved by the Institutional Review Boards at the Brigham and Women's Hospital (BWH) and the Icahn School of Medicine at Mount Sinai. Beth Israel Deaconess Medical Center (BIDMC) relied on BWH for review and oversight of the protocol. Written consent was obtained from all participants. Additional details about the cohort and methylation measures have been described elsewhere*(85, 86)*. PRISM consisted of 41.3% white European, 38.4% African American, 10.1% Hispanic and 10.1% other ancestries.

Birthweight

Newborn birthweight was abstracted from obstetric records. The distribution was checked for plausibility, with no values +/- 3 SD from the mean after restricting to the analytic dataset of full term births. Given the limited sample size, we treated birthweight as a continuous trait in grams.

Methylation measurements

Cord blood was collected prior to delivery of the placenta from all samples that could be retrieved during delivery. Cord blood was separated into plasma and buffy coat by centrifugation and buffy coat was stored at -20°C. DNA was isolated using Qiagen Tissue DNA extraction kits (Qiagen, Valencia CA) and quantified using an Implen Nanophotometer Pearl (Westlake Village, CA). 500ng of DNA was bisulfite treated using the EZ DNA Methylation-Gold™ Kit (Zymo Research, Orange, CA) and analyzed by the Illumina Infinium HumanMethylation450 BeadChip array. Samples were arranged on chips and plates with a stratified randomization followed by statistical checks for balance on birthweight z-score, gestational age, sex, and city of collection. The presence of failed arrays or outliers was checked with detection p-values (all samples passed with detection p-values <0.05 in >99% of probes) and through visualization of principal components analysis (PCA). Potential batch effects were further assessed with five pairs of technical replicates per tissue that were arranged across chips and plates. Sample identity was checked via imputed sex and agreement of genotype with paired tissues. Probes with detection p-values >0.05 or beadcount <3 in >1% of samples (a total of 1217 probes) were dropped using the wateRmelon pfilter function resulting in 484,360 remaining probes*(45)*. Data were preprocessed using background correction*(25)*, dye bias and probe type adjustment*(19)*. BMIQ (Beta Mixture Quantile dilation) intra-sample normalization was applied to all probes to adjust the methylation values of Infinium II probes into a statistical distribution characteristic of Infinium I probes.

Covariates

Data on maternal primiparity (dichotomous), education (categorical with five levels) as a proxy for socio-economic status, any smoking during pregnancy (dichotomized), pre-pregnancy body mass index, and maternal age were self-reported in questionnaires administered during the second or third trimester of pregnancy. Newborn sex and gestational age at birth were abstracted from the medical record.

Cell type correction

We used the Reinius-based Houseman method*(5, 6)* with the estimateCellCounts function in the Minfi package [8] in R [9] to estimate relative proportions of six white blood cell subtypes (CD4+ T-lymphocytes, CD8+ T-lymphocytes, NK (natural killer) cells, B-lymphocytes, monocytes and granulocytes).

Batch correction

We additionally corrected the analyses for batch effect by including the 96-well bisulfite conversion plate (laboratory batch) as a covariate (categorical variable with seven levels).

Exclusion criteria

From the main analysis we excluded 10 participants because of gestational diabetes, 9 participants because of gestational hypertension and 10 because pre-term borns.

Programming Research in Obesity, GRowth Environment and Social Stress (PROGRESS)

Design and study population

Programming Research in Obesity, GRowth Environment and Social Stress (PROGRESS) is a prospective birth cohort of healthy pregnant women. We recruited women if they were less than 20 weeks of gestation through the Mexican Social Security System between July 2007 and February 2011. We enrolled women greater than 18 years old that have access to a telephone and a plan to reside within Mexico City for the following 3 years. We excluded people with diagnosis of heart or kidney disease, use of steroids

or anti-epilepsy drugs and daily alcohol consumption. Women provided written informed consent for themselves and their children.

Birthweight

Birth length and weight were recorded at delivery or abstracted from the infants' hospital chart. At later study visits, length/height and weight were measured using the Infant-o-meter (Health O Meter Inc.) for children 1-12 months old and the Health-o-meter (Health O Meter Inc.) for children older than 12 months. For the analysis model we treated birthweight as a continuous variable in grams.

Methylation measurements

We collected umbilical cord venous blood at the time of delivery and stored whole blood in PAXgene™ (PreAnalytiX GmbH, Hombrechtikon Switzerland) tubes and extracted DNA using PAXgene kits. Umbilical cord blood samples were aliquoted and frozen until manual DNA extraction including a red blood cell lysis step followed by isopropanol and ethanol extraction of DNA from total white blood cells. Resulting DNA samples were randomized for plating and bisulfite converted and analyzed on the Illumina 450k BeadChip by Illumina FastTrack Services (Illumina Inc., San Diego CA), prior to preprocessing and quality control with the methylumi package.

Covariates

Major covariates include maternal pre-pregnancy BMI (calculated as BMI during 2nd trimester minus 2 kg/m2), parity, socio-economic status, smoking, age at birth and new born gender were obtained from baseline questionnaires, hospital records or field measurements. Gestational age was based on the difference between the birth date and the mother's report on enrolment of her last menstrual period.

Cell type correction

We estimated the proportions of the six white blood cell subtypes: CD4+ T-lymphocytes, CD8+ T-lymphocytes, NK (natural killer) cells, B-lymphocytes, monocytes and granulocytes, using the Reinius-based Houseman method with the *estimateCellCounts* function in the Minfi package of R.

Batch correction

We additionally corrected the analyses for batch effect by including the Illumina Infinium HumanMethylation450 BeadChip number.

Exclusion criteria

For the main analysis we excluded infants born preterm (<37 weeks, n=118), which automatically excluded the participants whose mothers experienced pre-eclampsia and hypertension during the pregnancy. This information was obtained from the mothers' questionnaires completed shortly after the delivery and combined with medical birth records.

Project Viva

Design and study population

Project Viva is a population-based prospective birth cohort in Eastern Massachusetts. Pregnant women at <22 weeks gestation with the ability to answer questions in English and singleton pregnancies were recruited at their first prenatal visit at a participating obstetric office from 1999 to 2002. Of 2218 live births, 1018 cord blood samples were collected. All women gave written informed consent for the study, and 507 mothers additionally gave written informed genetic consent. Institutional review boards at all participating institutions gave approval for this study*(87)*.

Birthweight

Newborn birthweight in grams was obtained from hospital medical records. We did not observe birthweight values +/- 5 SD from the mean.

Methylation measurements

For the 507 samples that gave genetic consent, DNA was extracted from cord blood using the Qiagen Puregene Kit (Valencia, CA) and sodium bisulphate converted using the EZ DNA Methylation-Gold Kit (Zymo Research, Irvine, CA). Methylation beta values (0=no methylation, 1=complete methylation) were measured using the Infinium HumanMethylation450 BeadChip (Illumina Inc., San Diego, USA). During sample quality control, 22 samples were removed due to genotype mismatch, sex mismatch, and low quality, leaving 485 samples. After performing background correction with the normal-exponential out-of-band (noob)*(25)* method with dye-bias correction, we adjusted for probe type using Beta-Mixture Quantile dilation (BMIQ)*(19)*. We removed 3091 non-CG probes, 11,648 sex chromosome probes, and 459 probes with detection p-value<0.05 in less than 75% of samples, leaving 470,411 probes for analysis.

Covariates

Data on maternal parity, maternal education (as a measure of socio-economic status), smoking, pre-pregnancy body mass index, maternal age at enrollment, newborn gender and gestational age at birth were collected via mailed questionnaires and clinical records.

Cell type correction
We used the Reinius-based Houseman method*(5, 6)* with the *estimateCellCounts* function in the Minfi package*(7)* in R*(8)* to estimate relative proportions of six white blood cell subtypes (CD4+ T-lymphocytes, CD8+ T-lymphocytes, NK (natural killer) cells, B-lymphocytes, monocytes and granulocytes).

Batch correction
We additionally corrected the analyses for batch effect using ComBat*(16)*, using Sentrix position within each of two scanning batches as the batch variable. The batch variable had 19 unique levels.

Exclusion criteria
Because analyses were stratified by self-reported race and we had very few non-white children, we excluded children of non-white mothers (n=141) in our analysis. For the main analysis of white children, we excluded n=15 participants: 12 infants born preterm (<37 weeks), 2 infants whose mothers had pre-gestational diabetes, and 1 infant whose mother did not have a recorded pre-pregnancy BMI. This information was obtained from questionnaires completed by the mothers shortly after delivery and combined with medical birth records.

The West Australian Pregnancy Cohort (Raine)
Design and study population
The Western Australia Pregnancy Cohort (Raine) study (http://www.rainestudy.org.au) is a longitudinal Australian birth cohort that has serially assessed the offspring of 2900 pregnant women from 18 weeks gestation in utero. Follow-up of the offspring has been undertaken at 1, 2, 3, 5, 8, 10, 14, 17 and 24 years*(88, 89)*.

Birthweight
Birthweight was measured by hospital midwives immediately after birth on standardized hospital neonatal scales.

Methylation measurements
DNA was extracted from whole blood samples obtained at 17-year-old follow up. Bisulphite conversion was prepared from whole blood cells by standard phenol:chloroform extraction and ethanol precipitation. Processing of the Illumina Infinium HumanMethylation450 BeadChips was carried out by the Centre for Molecular Medicine and Therapeutics (CMMT) (http://www.cmmt.ubc.ca). The raw IDAT files were imported into R using the rnb.run.import() function available in the RnBeads package*(90)*. Two packages were used to perform quality control checks of the samples; shinyMethyl*(91)* and MethylAid*(82)*. Three samples were evident as outliers and removed. We removed intentional SNP probes (n=65), sex chromosome probes (n=11,648), and probes with a detection p-value greater than 0.05 in any sample (n=10,777). A further 160 probes with low bead counts (bead counts less than 3 in more than 5% of samples) were removed. Probes were normalized using BMIQ*(19)* and an additional 94 probes were removed resulting in 462,833 probes for analysis.

Covariates
Maternal BMI was calculated using self-reported pre-pregnancy weight and height. Data on maternal age, income, parity and maternal smoking during pregnancy were assessed by questionnaires at 18 and 34 weeks pregnancy. Maternal age at delivery was derived from the mother's report of her own and her baby's dates of birth. We used plate and plate position as technical batch variables. Gestational age was based on the date of the last menstrual period, unless there was discordance with ultrasound measurements at less than 18 weeks gestation. In those cases of discordance of more than 7 days, the estimate was based on ultrasound biometry at 18 weeks. First date of last menstrual period (LMP) was recorded by the mother at the 16-18-week survey. Weight was measured by Wedderburn Chair Scales to the nearest 100 grams and height to the nearest 0.1 cm using a Holtain Stadiometer, at the 17 year follow up. Body mass index (BMI) was calculated as weight in kilograms divided by height in metres squared. Current smoking status at 17 years of age was derived from an online confidential questionnaire answered by the adolescent, in response to the question "have you smoked in the last 4 weeks?"

Cell type correction
The relative proportions of CD8+ T-lymphocytes, CD4+ T-lymphocytes, NK cells, B-lymphocytes, monocytes and granulocytes were inferred using the estimateCellCounts function within minfi*(29)*, based on the Houseman method*(6)*.

Batch correction
Plate and row number were included in all models to adjust for potential batch effects.

Exclusion criteria
Individuals whose mother had maternal diabetes or pre-eclampsia were excluded, as well as multiple births and pre-term births.

Rhode Island Child Health Study (RICHS)

Design and study population

Study participants are part of the Rhode Island Child Health Study (RICHS), which enrolled 840 mother-infant pairs following delivery at Women and Infants Hospital (Providence, RI, USA) from 2009-2014*(92)*. Study participants provided written informed consent and all protocols were approved by the Institutional Review Boards at Women and Infants Hospital and Dartmouth College. Eligibility criteria included being between the ages of 18 and 40 years, free of life threatening conditions, no congenital or chromosomal abnormalities, and gestational time greater than 37 weeks. Infants were singleton births, with gestation to term (≥ 37 weeks). Term infants born small for gestational age (SGA, <10th percentile), or large for gestational age (LGA, >90th percentile), were oversampled, then infants appropriate for gestational age (AGA, ≥10th percentile and ≤90th percentile) matched on gender, gestational age (±3 days), and maternal age (±2 years) were also enrolled. Structured medical records review was performed to collect maternal inpatient information from the delivery as well as anthropometric and clinical data. After delivery but prior to discharge, an interviewer-administered questionnaire was used to obtain information on exposures, demographics, and lifestyle factors. Cord blood samples were obtained as residual tissues from samples collected at delivery and maintained in the Department of Pathology for potential clinical use. Once the infant was discharged, remaining cord blood samples were collected for the study. A total of 450 newborn cord blood samples were collected, and the major demographic features of the infants with cord blood samples did not differ from those of the whole cohort.

Birthweight

Participants also consented to a medical record review, which allowed for additional details on birth outcomes and delivery.

Methylation measurements

This study consisted of the first participants born in the study with available cord blood samples for DNA methylation analysis, complete information for all covariates (n=89). DNA was bisulfite converted using the EZ DNA Methylation kit and subsequently subjected to epigenome-wide DNA methylation assessment using the Illumina Infinium HumanMethylation450 BeadChip at the University of Minnesota Genomics Core Facility following standardized protocols. Post-array processing was conducted in the 'minfi' package in R. Array control probes were used to assess the quality of our samples and evaluate potential poor bisulfite conversion or color-specific issues for each array. Probes with detection p-values > 0.01 in at least one sample were removed. Data was then normalized using functional normalization (funNorm).

Covariates

Self-reported smoking during pregnancy (*any smoking during pregnancy vs. no smoking during pregnancy*), maternal highest educational attainment (*high school graduation or less vs. at least some post high school education*), parity (*ever previously gave birth vs. never previously gave birth*), and maternal age (*continuous*) were collected via questionnaire. Infant sex and gestational age were collected via medical records abstraction.

Cell type correction

Proportions of cell types were estimated from the 450K DNAM via the estimateCellCounts function*(5, 6)* within the 'minfi' package in R. Due to the sum of the six estimated cell proportions adding to 1.0 for each participant, only five of the cell-types were added to the models as covariates (granulocytes were excluded).

Batch correction

Batch effects were removed from the methylation data via ComBat; removal of batch effects was confirmed with principal components analysis.

Exclusion criteria

For the main analysis we excluded 7 participants whose mothers experienced diabetes during pregnancy. This information was obtained from questionnaires completed by the mothers shortly after delivery.

The Swedish Twin study On Prediction and Prevention of Asthma (STOPPA)

Design and study population

The Swedish Twin study On Prediction and Prevention of Asthma (STOPPA)*(93)* is a twin cohort study including 752 individuals. Twins 9-14 years of age were selected from an on-going data collection within the Child and Adolescent Twin study in Sweden (CATSS)*(94)* based on the pair's asthma status. Asthma concordant (ACC), asthma discordant (ADC) and healthy concordant (HCC) pairs were included and invited to take part in test centre visits including clinical examination, questionnaires, lung function testing and collection of biosamples. The study was approved by the regional ethical review board in Stockholm, Sweden. Written informed consent was collected from the study participants and their parents. Further details regarding STOPPA have been provided in a separate publication*(93)*. To allow for both twins from full pairs to be retained within the sample, generalized estimating equation (GEE) models were used in place of robust linear regression in STOPPA. By specifying twin pairs as clusters,

the GEE method produces robust standard errors and corrects for within-cluster (i.e. within-pair) correlations. The parameter estimates themselves are not affected. For these analyses the *drgee* R package was used*(95)*.

Birthweight
The study population has been linked to the Swedish population-based Medical Birth Register (MBR), from which birthweight was available from birth records. No values fell outside +/-5 SD. Because only one individual with birthweight >4000 grams remained within the study population following exclusions, only analyses featuring the continuous phenotype were performed using the STOPPA data.

Methylation measurements
STOPPA biosamples included whole blood samples from n=708 twins, i.e. all twins for whom blood sample collection was possible on the day of clinical examination. Following exclusions based on predefined criteria (n=339), missing data on covariates or outcomes (n=118), and sample quality (n=2), 249 twins remained for the current study. Of the 249 individuals with complete information, n=8 were below 10 (but above 9) years of age. Because this group was too small to run any analyses within separately, they were included in the adolescent group. DNA was extracted from whole blood (collected in a 4ml EDTA tube) using the Chemagic Star 400 kit (PerkinElmer chemagen, Baesweiler, Aachen, Germany) according to a standardized protocol. Samples were allocated between analysis plates and chips by complete randomization, with the exception that samples from twin pairs were kept within the same chip to allow for within-pair comparisons free of batch effects. Analyses were performed at the Mutation Analysis Facility (MAF) at Karolinska Institutet using the Infinium HumanMethylation450 Beadchip Kit (Illumina, Inc., San Diego, California, USA). Quality control, sample and probe filtering was performed using RnBeads*(90)*. Predicted gender and phenotype-based sex were compared, and matched for all samples. Probes overlapping with single nucleotide polymorphisms or specific nucleotide contexts (n=15,489), due to unreliable measurements (defined as detection p-values > 5*10-8, resulting in the filtering of n=3,663 probes and 2 samples), or which were located on sex chromosomes (n=10,942) were filtered out, leaving 455,483 CpG probes for the final analysis. The data set was normalized using the dasen method*(45)*.

Covariates
Gestational age, child's sex, maternal smoking during pregnancy (any/never), maternal BMI (calculated from weight and height at the first antenatal care visit), and parity were available from maternal antenatal and birth records (MBR). Maternal social class was defined by the highest self-reported education level achieved by the mother at the time of clinical examination, and categorized as "Less than high school level", "High school or equivalent degree", "Some college or university studies" or "3+ years of college/university or a university degree." Because recruitment to STOPPA was based on asthma case/control status within twin pairs, the case/control variable for asthma status within the individual was included as a covariate. Of the final 249 individuals, 114 (45.8%) had been recruited as asthma cases and 135 (54.2%) as controls. Study participants' own current smoking at the time of data collection had not been assessed and was therefore not included in the STOPPA sensitivity model.

Cell type correction
The relative proportions of CD8+ T-lymphocytes, CD4+ T-lymphocytes, NK cells, B-lymphocytes, monocytes and granulocytes were inferred using the estimateCellCounts function within minfi*(29)*, based on the Houseman method*(6)*.

Batch correction
Plate number was included in all models to adjust for potential batch effects.

Exclusion criteria
For the main analysis we excluded n=339 participants: 323 infants born preterm (<37 weeks), and whose mothers experienced pre-eclampsia (n=24) or diabetes (n=2) during pregnancy. This information was obtained from medical birth records.

The Gambia
Design and study population
The data was collected as part of a study in The Gambia (in Sub-Saharan West Africa) identifying biomarkers and understanding mechanisms for the relationship between aflatoxin exposure and child stunting. 251 whole blood samples (3 ml) were collected from children aged 2 years as part of the Early Nutrition and Immune Development (ENID) Trial*(96)*. For DNA extraction the blood was centrifuged, and the plasma was removed; DNA was then extracted from the resulting cell pellet using a salting out technique. For RNA extraction, a sample of whole blood was taken prior to centrifugation and stored in RNALater. All ethical approvals were obtained from the joint Gambia Government/MRC Unit, The Gambia Ethics Committee, and full informed consent was obtained from all participating families, prior to enrolment into the study. Of the samples collected, 243 samples had DNA methylation data (HM450), of which 84 samples also had expression data (HumanHT-12 v4 Expression BeadChip); both datasets were generated at the Epigenetics Group, International Agency for Research on Cancer, Lyon, France.

Birthweight

Birthweight was measured within 72 hours of delivery by a trained member of the study team, using a digital infant scale (Seca 334) and to the nearest 10g. We did not observe birthweight values +/- 5 SD from the mean. For the main analysis model, we treated birthweight as a continuous trait in grams, and, for the dichotomous model, we categorised birthweight in high (>4000g) versus normal (2500-4000g) birthweight.

Methylation measurements
Within The Gambia birth cohort, we selected 243 infants for the methylation study. From these infants, peripheral blood DNA was used for the epigenome-wide DNA methylation analyses. Bisulfite conversion (600 ng DNA/sample) was performed using the EZ-96 DNA methylation kit (Zymo research Corporation, Irvine, USA). Then the Infinium HumanMethylation450 BeadChip (Illumina Inc., San Diego, USA) was used to measure the methylation level as a beta value ranging from 0 (no methylation) to 1 (complete methylation). During the quality control, the binomial distribution was evident in all samples and coherent across samples, and we observed no gender mismatches, which we confirmed using the getSex function. We performed Functional normalization (FunNorm)*(18)* from minfi R package*(29)* to remove unwanted variation by regressing out variability explained by the control probes present on the array. We filtered out cross-reactive probes and probes on X or Y chromosomes, resulting in 470,964 remaining CpGs. We have added the ethnicity covariable for our specific analysis because our data comprises several ethnicities which we reclassified in 2 groups: Mandinka (n = 189) and other (n = 23).

Covariates
The covariates analysed were maternal parity, socio-economic status, smoking, pre-pregnancy body mass index, age at birth, newborn gender, gestational age at birth and ethnicity.

Cell type correction
We used the Reinius-based Houseman method*(5, 6)* with the *estimateCellCounts* function in the Minfi package*(29)* in R to estimate relative proportions of six white blood cell subtypes (CD4+ T-lymphocytes, CD8+ T-lymphocytes, NK (natural killer) cells, B-lymphocytes, monocytes and granulocytes).

Batch correction
We additionally corrected the analyses for batch effect using surrogate variable analysis (SVA)*(30)*.

Exclusion criteria
In the methylation subset of The Gambia cohort we excluded infants born preterm (<37 weeks) (n=2), and samples that didn't have complete data for covariates (n=29). Data on maternal diabetes and pre-eclampsia was not available in this cohort.

Gene expression analysis
The Gambia gene expression data was collected at 2 years of age. Whole blood was collected in Gambia and shipped to IARC, France, on dry ice where they were stored at -80 C. Samples were later thawed and extracted using RiboPure kit from Ambion (RNA purification kit for blood). All samples had a RIN greater than the acceptable threshold of 6.5 (range = 6.5 – 9.8; median = 9.1, mean ± SD = 9.0 ± 0.7). The high median and mean were in line with the observation that the vast majority of RNA samples had a RIN ~9.0, so we did not adjust for RNA quality. Gene expression data was generated using IlluminaHumanHT-12 v4 Expression BeadChip Kit at IARC, Lyon, France. Gene expression was normalized with the neqc R package, and expression transcripts were annotated using HumanHT-12 v4.0 Manifest File. Out of 104 samples that had expression data, 20 were excluded because they did not have methylation data. The final sample size was 84.

DNA methylation, DNA extraction and preprocessing has been described in the cohort-specific methods above. In brief, data quality was inspected using boxplots for the distribution of methylated and unmethylated signals, and inter-sample relationship (including gender mismatches) using multidimensional scaling plots and unsupervised clustering. QQ-plots of p values were used to rule out potential data inflation after statistical analyses. The remaining dataset was normalized using the funnorm normalization*(18)* of the minfi package*(29)*. Batch effects were then corrected with surrogate variable analysis (7 surrogate variables identified and adjusted for), and the effect of sex and blood cell proportions estimates were controlled out in a linear regression model. Cell proportions were estimated using the Houseman method*(6)*.

Only transcripts inside a 500kb window of a selected CpG (250 kb downstream and 250 kb upstream) were considered in the analysis. The model was controlled for technical and unwanted biological variation when estimating gene expression residuals, 8 surrogate variables were detected by surrogate variable analysis and added to a second model including sex and cell estimates.

References

1. A. Fraser, C. Macdonald-Wallis, K. Tilling, A. Boyd, J. Golding, G. Davey Smith, J. Henderson, J. Macleod, L. Molloy, A. Ness, S. Ring, S. M. Nelson, D. A. Lawlor, Cohort Profile: the Avon Longitudinal Study of Parents and Children: ALSPAC mothers cohort, *Int J Epidemiol* **42**, 97–110 (2013).

2. A. Boyd, J. Golding, J. Macleod, D. A. Lawlor, A. Fraser, J. Henderson, L. Molloy, A. Ness, S. Ring, G. Davey Smith, Cohort Profile: the "children of the 90s"--the index offspring of the Avon Longitudinal Study of Parents and Children, *Int J Epidemiol* **42**, 111–127 (2013).

3. C. L. Relton, T. Gaunt, W. McArdle, K. Ho, A. Duggirala, H. Shihab, G. Woodward, O. Lyttleton, D. M. Evans, W. Reik, Y. L. Paul, G. Ficz, S. E. Ozanne, A. Wipat, K. Flanagan, A. Lister, B. T. Heijmans, S. M. Ring, G. D. Smith, Data resource profile: Accessible resource for integrated epigenomic studies (ARIES), *Int. J. Epidemiol.* **44**, 1181–1190 (2015).

4. C. Macdonald-Wallis, K. Tilling, A. Fraser, S. M. Nelson, D. A. Lawlor, Associations of blood pressure change in pregnancy with fetal growth and gestational age at delivery: findings from a prospective cohort., *Hypertens. (Dallas, Tex. 1979)* **64**, 36–44 (2014).

5. L. E. Reinius, N. Acevedo, M. Joerink, G. Pershagen, S.-E. Dahlén, D. Greco, C. Söderhäll, A. Scheynius, J. Kere, A. H. Ting, Ed. Differential DNA Methylation in Purified Human Blood Cells: Implications for Cell Lineage and Studies on Disease Susceptibility, *PLoS One* **7**, e41361 (2012).

6. E. A. Houseman, W. P. Accomando, D. C. Koestler, B. C. Christensen, C. J. Marsit, H. H. Nelson, J. K. Wiencke, K. T. Kelsey, DNA methylation arrays as surrogate measures of cell mixture distribution, *BMC Bioinformatics* **13**, 1–16 (2012).

7. A. E. Jaffe, R. A. Irizarry, Accounting for cellular heterogeneity is critical in epigenome-wide association studies, *Genome Biol.* **15**, R31 (2014).

8. R Core Team, R Core Team (2014). R: A language and environment for statistical computing., *R Found. Stat. Comput. Vienna, Austria. URL http//www.R-project.org/.* , R Foundation for Statistical Computing (2014).

9. M. Wickman, I. Kull, G. Pershagen, S. L. Nordvall, The BAMSE project: presentation of a prospective longitudinal birth cohort study., *Pediatr. allergy Immunol.* **13 Suppl 1**, 11–13 (2002).

10. S. Ekström, J. Magnusson, I. Kull, T. Lind, C. Almqvist, E. Melén, A. Bergström, Maternal body mass index in early pregnancy and offspring asthma, rhinitis and eczema up to 16 years of age., *Clin. Exp. Allergy* **45**, 283–91 (2015).

11. S. Ekström, J. Magnusson, I. Kull, N. Andersson, M. Bottai, M. B. Pour, E. Melen, A. Bergström, Body Mass Index Development and Asthma Throughout Childhood, *Am. J. Epidemiol.* **186**, 255–263 (2017).

12. A. Gref, S. K. Merid, O. Gruzieva, S. Ballereau, A. Becker, T. Bellander, A. Bergstrom, Y. Bosse, M. Bottai, M. Chan-Yeung, E. Fuertes, D. Ierodiakonou, R. Jiang, S. Joly, M. Jones, M. S. Kobor, M. Korek, A. L. Kozyrskyj, A. Kumar, N. Lemonnier, E. MacIntyre, C. Menard, D. Nickle, M. Obeidat, J. Pellet, M. Standl, A. Saaf, C. Soderhall, C. M. T. Tiesler, M. van den Berge, J. M. Vonk, H. Vora, C.-J. Xu, J. M. Anto, C. Auffray, M. Brauer, J. Bousquet, B. Brunekreef, W. J. Gauderman, J. Heinrich, J. Kere, G. H. Koppelman, D. Postma, C. Carlsten, G. Pershagen, E. Melen, Genome-Wide Interaction Analysis of Air Pollution Exposure and Childhood Asthma with Functional Follow-up., *Am. J. Respir. Crit. Care Med.* **195**, 1373–1383 (2017).

13. Y. A. Chen, M. Lemire, S. Choufani, D. T. Butcher, D. Grafodatskaya, B. W. Zanke, S. Gallinger, T. J. Hudson, R. Weksberg, Discovery of cross-reactive probes and polymorphic CpGs in the Illumina Infinium HumanMethylation450 microarray, *Epigenetics* **8**, 203–209 (2013).

14. R. Pidsley, C. C. Y Wong, M. Volta, K. Lunnon, J. Mill, L. C. Schalkwyk, A data-driven approach to preprocessing Illumina 450K methylation array data, *BMC Genomics* **14**, 293 (2013).

15. R Core Team, R. D. C. Team, R: A language and environment for statistical computing.

16. W. E. Johnson, C. Li, A. Rabinovic, Adjusting batch effects in microarray expression data using empirical Bayes methods, *Biostatistics* **8**, 118–127 (2007).

17. X. Ma, P. A. Buffler, J. L. Wiemels, S. Selvin, C. Metayer, M. Loh, M. B. Does, J. K. Wiencke, Ethnic difference in daycare attendance, early infections, and risk of childhood acute lymphoblastic leukemia, *Cancer Epidemiol. Biomarkers Prev.* **14**, 1928–1934 (2005).

18. J.-P. Fortin, A. Labbe, M. Lemire, B. W. Zanke, T. J. Hudson, E. J. Fertig, C. M. Greenwood, K. D. Hansen, Functional normalization of 450k methylation array data improves replication in large cancer studies, *Genome Biol.* **15**, 503 (2014).

19. A. E. Teschendorff, F. Marabita, M. Lechner, T. Bartlett, J. Tegner, D. Gomez-Cabrero, S. Beck, A Beta-Mixture Quantile Normalisation method for correcting probe design bias in Illumina Infinium 450k DNA methylation data., *Bioinformatics* **29**, 189–

196 (2012).

20. J. E. Joo, E. M. Wong, L. Baglietto, C.-H. Jung, H. Tsimiklis, D. J. Park, N. C. Wong, D. R. English, J. L. Hopper, G. Severi, G. G. Giles, M. C. Southey, The use of DNA from archival dried blood spots with the Infinium HumanMethylation450 array., *BMC Biotechnol.* **13**, 23 (2013).

21. B. Eskenazi, A. Bradman, E. a. Gladstone, S. Jaramillo, K. Birch, N. Holland, CHAMACOS, A Longitudinal Birth Cohort Study: Lessons from the Fields, *J. Child. Heal.* **1**, 3–27 (2003).

22. B. Eskenazi, K. Harley, A. Bradman, E. Weltzien, N. P. Jewell, D. B. Barr, C. E. Furlong, N. T. Holland, Association of in utero organophosphate pesticide exposure and fetal growth and length of gestation in an agricultural population, *Environ. Health Perspect.* **112**, 1116–1124 (2004).

23. P. Yousefi, K. Huen, R. A. Schall, A. Decker, E. Elboudwarej, H. Quach, L. Barcellos, N. Holland, Considerations for normalization of DNA methylation data by Illumina 450K BeadChip assay in population studies, *Epigenetics* **8**, 1141–1152 (2013).

24. R. McConnell, K. Berhane, L. Yao, M. Jerrett, F. Lurmann, F. Gilliland, N. Künzli, J. Gauderman, E. Avol, D. Thomas, J. Peters, Traffic, susceptibility, and childhood asthma, *Environ. Health Perspect.* **114**, 766–772 (2006).

25. T. J. Triche, D. J. Weisenberger, D. Van Den Berg, P. W. Laird, K. D. Siegmund, Low-level processing of Illumina Infinium DNA Methylation BeadArrays, *Nucleic Acids Res.* **41**, e90 (2013).

26. N. Touleimat, J. Tost, Complete pipeline for Infinium ® Human Methylation 450K BeadChip data processing using subset quantile normalization for accurate DNA methylation estimation, *Epigenomics* **4**, 325–341 (2012).

27. J. K. Pritchard, M. Stephens, P. Donnelly, Inference of population structure using multilocus genotype data, *Genetics* **155**, 945–959 (2000).

28. C. J. Newschaffer, L. A. Croen, M. D. Fallin, I. Hertz-Picciotto, D. V Nguyen, N. L. Lee, C. A. Berry, H. Farzadegan, H. N. Hess, R. J. Landa, S. E. Levy, M. L. Massolo, S. C. Meyerer, S. M. Mohammed, M. C. Oliver, S. Ozonoff, J. Pandey, A. Schroeder, K. M. Shedd-Wise, Infant siblings and the investigation of autism risk factors, *J. Neurodev. Disord.* **4**, 0 (2012).

29. M. J. Aryee, A. E. Jaffe, H. Corrada-Bravo, C. Ladd-Acosta, A. P. Feinberg, K. D. Hansen, R. A. Irizarry, Minfi: A flexible and comprehensive Bioconductor package for the analysis of Infinium DNA methylation microarrays, *Bioinformatics* **30**, 1363–1369 (2014).

30. J. T. Leek, J. D. Storey, Capturing heterogeneity in gene expression studies by surrogate variable analysis, *PLoS Genet.* **3**, 1724–1735 (2007).

31. P. Vineis, M. Chadeau-Hyam, H. Gmuender, J. Gulliver, Z. Herceg, J. Kleinjans, M. Kogevinas, S. Kyrtopoulos, M. Nieuwenhuijsen, D. H. Phillips, N. Probst-Hensch, A. Scalbert, R. Vermeulen, C. P. Wild, The exposome in practice: Design of the EXPOsOMICS project, *Int. J. Hyg. Environ. Health* **220**, 142–151 (2017).

32. S. Farchi, F. Forastiere, L. Vecchi Brumatti, S. Alviti, A. Arnofi, T. Bernardini, M. Bin, S. Brescianini, V. Colelli, R. Cotichini, M. Culasso, P. De Bartolo, L. Felice, V. Fiano, A. Fioritto, A. Frizzi, L. Gagliardi, G. Giorgi, C. Grasso, F. La Rosa, C. Loganes, P. Lorusso, V. Martini, F. Merletti, E. Medda, V. Montelatici, I. Mugelli, S. Narduzzi, L. Nisticò, L. Penna, E. Piscianz, C. Piscicelli, G. Poggesi, D. Porta, A. Ranieli, G. Rapisardi, A. Rasulo, L. Richiardi, F. Rusconi, L. Serino, M. A. Stazi, V. Toccaceli, T. Todros, V. Tognin, M. Trevisan, E. Valencic, P. Volpi, V. Ziroli, L. Ronfani, D. Di Lallo, Piccolipiù, a multicenter birth cohort in Italy: protocol of the study, *BMC Pediatr.* **14**, 36 (2014).

33. L. Chatzi, V. Leventakou, M. Vafeiadi, K. Koutra, T. Roumeliotaki, G. Chalkiadaki, M. Karachaliou, V. Daraki, A. Kyriklaki, M. Kampouri, E. Fthenou, K. Sarri, M. Vassilaki, M. Fasoulaki, P. Bitsios, A. Koutis, E. G. Stephanou, M. Kogevinas, Cohort Profile: The Mother-Child Cohort in Crete, Greece (Rhea Study), *Int. J. Epidemiol.* , 1392–1393 (2017).

34. B. G. Janssen, N. Madlhoum, W. Gyselaers, E. Bijnens, D. B. Clemente, B. Cox, J. Hogervorst, L. Luyten, D. S. Martens, M. Peusens, M. Plusquin, E. B. Provost, H. A. Roels, N. D. Saenen, M. Tsamou, A. Vriens, E. Winckelmans, K. Vrijens, T. S. Nawrot, Cohort Profile: The ENVIRonmental influence ON early AGEing (ENVIRONAGE): a birth cohort study, *Int. J. Epidemiol.* **46**, 1386–1387m (2017).

35. G. Schoeters, E. Den Hond, A. Colles, I. Loots, B. Morrens, H. Keune, L. Bruckers, T. Nawrot, I. Sioen, S. De Coster, N. Van Larebeke, V. Nelen, E. Van de Mieroop, J. Vrijens, K. Croes, K. Goeyens, W. Baeyens, Concept of the Flemish human biomonitoring programme, *Int. J. Hyg. Environ. Health* **215**, 102–108 (2012).

36. Flemish Center of Expertise on Environment and Health (available at http://www.milieu-en-gezondheid.be/en/home).

37. G. Koppen, E. Den Hond, V. Nelen, E. Van De Mieroop, L. Bruckers, M. Bilau, H. Keune, N. Van Larebeke, A. Covaci, H. Van De Weghe, C. Schroijen, K. Desager, M. Stalpaert, W. Baeyens, G. Schoeters, Organochlorine and heavy metals in newborns: Results

from the Flemish Environment and Health Survey (FLEHS 2002-2006), *Environ. Int.* **35**, 1015–1022 (2009).

38. J. Maksimovic, L. Gordon, A. Oshlack, SWAN: Subset-quantile Within Array Normalization for Illumina Infinium HumanMethylation450 BeadChips, *Genome Biol.* **13**, R44 (2012).

39. C. L'Abee, P. J. Sauer, M. Damen, J. P. Rake, H. Cats, R. P. Stolk, Cohort Profile: the GECKO Drenthe study, overweight programming during early childhood, *Int J Epidemiol* **37**, 486–489 (2008).

40. L. K. Küpers, X. Xu, S. A. Jankipersadsing, A. Vaez, S. la Bastide-van Gemert, S. Scholtens, I. M. Nolte, R. C. Richmond, C. L. Relton, J. F. Felix, L. Duijts, J. B. van Meurs, H. Tiemeier, V. W. Jaddoe, X. Wang, E. Corpeleijn, H. Snieder, DNA methylation mediates the effect of maternal smoking during pregnancy on birthweight of the offspring, *Int. J. Epidemiol.* **44**, 1224–1237 (2015).

41. L. Guillemette, C. Allard, M. Lacroix, J. Patenaude, M.-C. Battista, M. Doyon, J. Moreau, J. Ménard, L. Bouchard, J.-L. Ardilouze, P. Perron, M.-F. Hivert, Genetics of Glucose regulation in Gestation and Growth (Gen3G): a prospective prebirth cohort of mother–child pairs in Sherbrooke, Canada, *BMJ Open* **6**, e010031 (2016).

42. M. N. Kooijman, C. J. Kruithof, C. M. van Duijn, L. Duijts, O. H. Franco, M. H. van IJzendoorn, J. C. de Jongste, C. C. W. Klaver, A. van der Lugt, J. P. Mackenbach, H. A. Moll, R. P. Peeters, H. Raat, E. H. H. M. Rings, F. Rivadeneira, M. P. van der Schroeff, E. A. P. Steegers, H. Tiemeier, A. G. Uitterlinden, F. C. Verhulst, E. Wolvius, J. F. Felix, V. W. V. Jaddoe, The Generation R Study: design and cohort update 2017, *Eur. J. Epidemiol.* **31**, 1243–1264 (2016).

43. The Genome of the Netherlands Consortium, Whole-genome sequence variation, population structure and demographic history of the Dutch population, *Nat. Genet.* **46**, 818–825 (2014).

44. M. J. Bonder, S. Kasela, M. Kals, R. Tamm, K. Lokk, I. Barragan, W. A. Buurman, P. Deelen, J.-W. Greve, M. Ivanov, S. S. Rensen, J. V van Vliet-Ostaptchouk, M. G. Wolfs, J. Fu, M. H. Hofker, C. Wijmenga, A. Zhernakova, M. Ingelman-Sundberg, L. Franke, L. Milani, Genetic and epigenetic regulation of gene expression in fetal and adult human livers., *BMC Genomics* **15**, 860 (2014).

45. R. Pidsley, C. C. Y Wong, M. Volta, K. Lunnon, J. Mill, L. C. Schalkwyk, A data-driven approach to preprocessing Illumina 450K methylation array data., *BMC Genomics* **14**, 293 (2013).

46. O. Gishti, R. Gaillard, R. Manniesing, M. Abrahamse-Berkeveld, E. M. van der Beek, D. H. M. Heppe, E. A. P. Steegers, A. Hofman, L. Duijts, B. Durmuş, V. W. V. Jaddoe, Fetal and Infant Growth Patterns Associated With Total and Abdominal Fat Distribution in School-Age Children, *J. Clin. Endocrinol. Metab.* **99**, 2557–2566 (2014).

47. T. E. Strandberg, A. L. Järvenpää, H. Vanhanen, P. M. McKeigue, Birth outcome in relation to licorice consumption during pregnancy, *Am. J. Epidemiol.* **153**, 1085–1088 (2001).

48. K. Räikkönen, S. Martikainen, A. K. Pesonen, J. Lahti, K. Heinonen, R. Pyhälä, M. Lahti, S. Tuovinen, K. Wehkalampi, S. Sammallahti, L. Kuula, S. Andersson, J. G. Eriksson, A. Ortega-Alonso, R. M. Reynolds, T. E. Strandberg, J. R. Seckl, E. Kajantie, Maternal licorice consumption during pregnancy and pubertal, cognitive, and psychiatric outcomes in children, *Am. J. Epidemiol.* **185**, 317–328 (2017).

49. M. E. Price, A. M. Cotton, L. L. Lam, P. Farré, E. Emberly, C. J. Brown, W. P. Robinson, M. S. Kobor, Additional annotation enhances potential for biologically-relevant analysis of the Illumina Infinium HumanMethylation450 BeadChip array, *Epigenetics Chromatin* **6**, 4 (2013).

50. D. L. McCartney, R. M. Walker, S. W. Morris, A. M. McIntosh, D. J. Porteous, K. L. Evans, Identification of polymorphic and off-target probe binding sites on the Illumina Infinium MethylationEPIC BeadChip, *Genomics Data* **9**, 22–24 (2016).

51. L. Paternoster, D. M. Evans, E. Aagaard Nohr, C. Holst, V. Gaborieau, P. Brennan, A. Prior Gjesing, N. Grarup, D. R. Witte, T. Jørgensen, A. Linneberg, T. Lauritzen, A. Sandbaek, T. Hansen, O. Pedersen, K. S. Elliott, J. P. Kemp, B. St. Pourcain, G. McMahon, D. Zelenika, J. Hager, M. Lathrop, N. J. Timpson, G. Davey Smith, T. I. A. Sørensen, Genome-wide population-based association study of extremely overweight young adults - the GOYA study, *PLoS One* **6** (2011), doi:10.1371/journal.pone.0024303.

52. E. A. Nohr, N. J. Timpson, C. S. Andersen, G. Davey Smith, J. Olsen, T. I. A. Sørensen, P. Middleton, Ed. Severe Obesity in Young Women and Reproductive Health: The Danish National Birth Cohort, *PLoS One* **4**, e8444 (2009).

53. A. P. Starling, J. T. Brinton, D. H. Glueck, A. L. Shapiro, C. S. Harrod, A. M. Lynch, A. M. Siega-Riz, D. Dabelea, Associations of maternal BMI and gestational weight gain with neonatal adiposity in the Healthy Start study, *Am. J. Clin. Nutr.* **101**, 302–309 (2015).

54. A. L. B. Shapiro, S. J. Schmiege, J. T. Brinton, D. Glueck, T. L. Crume, J. E. Friedman, D. Dabelea, Testing the fuel-mediated hypothesis: maternal insulin resistance and glucose mediate the association between maternal and neonatal adiposity, the Healthy Start study, *Diabetologia* **58**, 937–941 (2015).

55. M. Guxens, F. Ballester, M. Espada, M. F. Fernández, J. O. Grimalt, J. Ibarluzea, N. Olea, M. Rebagliato, A. Tardón, M. Torrent, J. Vioque, M. Vrijheid, J. Sunyer, Cohort profile: The INMA-INfancia y Medio Ambiente-(environment and childhood) project, *Int.*

J. Epidemiol. **41**, 930–940 (2012).

56. S. C. Westerway, A. Davison, S. Cowell, Ultrasonic fetal measurements: new Australian standards for the new millennium., *Aust. N. Z. J. Obstet. Gynaecol.* **40**, 297–302 (2000).

57. J. T. Leek, W. E. Johnson, H. S. Parker, A. E. Jaffe, J. D. Storey, The SVA package for removing batch effects and other unwanted variation in high-throughput experiments, *Bioinformatics* **28**, 882–883 (2012).

58. S. H. Arshad, W. Karmaus, H. Zhang, J. W. Holloway, Multigenerational cohorts in patients with asthma and allergy, *J. Allergy Clin. Immunol.* **139**, 415–421 (2017).

59. B. Lehne, A. W. Drong, M. Loh, W. Zhang, W. R. Scott, S.-T. Tan, U. Afzal, J. Scott, M.-R. Jarvelin, P. Elliott, M. I. McCarthy, J. S. Kooner, J. C. Chambers, A coherent approach for analysis of the Illumina HumanMethylation450 BeadChip improves data quality and performance in epigenome-wide association studies, *Genome Biol.* **16**, 37 (2015).

60. S. Scholtens, N. Smidt, M. A. Swertz, S. J. L. Bakker, A. Dotinga, J. M. Vonk, F. Van Dijk, S. K. R. Van Zon, C. Wijmenga, B. H. R. Wolffenbuttel, R. P. Stolk, Cohort Profile: LifeLines, a three-generation cohort study and biobank, *Int. J. Epidemiol.* **44**, 1172–1180 (2015).

61. R. P. Stolk, J. G. M. Rosmalen, D. S. Postma, R. A. De Boer, G. Navis, J. P. J. Slaets, J. Ormel, B. H. R. Wolffenbuttel, Universal risk factors for multifactorial diseases: LifeLines: A three-generation population-based study, *Eur. J. Epidemiol.* **23**, 67–74 (2008).

62. P. Magnus, C. Birke, K. Vejrup, A. Haugan, E. Alsaker, A. K. Daltveit, M. Handal, M. Haugen, G. Høiseth, G. P. Knudsen, L. Paltiel, P. Schreuder, K. Tambs, L. Vold, C. Stoltenberg, Cohort Profile Update: The Norwegian Mother and Child Cohort Study (MoBa), *Int. J. Epidemiol.* **45**, 382–388 (2016).

63. S. E. Håberg, S. J. London, P. Nafstad, R. M. Nilsen, P. M. Ueland, S. E. Vollset, W. Nystad, Maternal folate levels in pregnancy and asthma in children at age 3 years*J. Allergy Clin. Immunol.* **127**, 262–264 (2011).

64. B. R. Joubert, S. E. Haberg, R. M. Nilsen, X. Wang, S. E. Vollset, S. K. Murphy, Z. Huang, C. Hoyo, O. Midttun, L. A. Cupul-Uicab, P. M. Ueland, M. C. Wu, W. Nystad, D. A. Bell, S. D. Peddada, S. J. London, 450K epigenome-wide scan identifies differential DNA methylation in newborns related to maternal smoking during pregnancy, *Env. Heal. Perspect* **120**, 1425–1431 (2012).

65. B. R. Joubert, J. F. Felix, P. Yousefi, K. M. Bakulski, A. C. Just, C. Breton, S. E. Reese, C. A. Markunas, R. C. Richmond, C. J. Xu, L. K. K??pers, S. S. Oh, C. Hoyo, O. Gruzieva, C. S??derh??ll, L. A. Salas, N. Ba??z, H. Zhang, J. Lepeule, C. Ruiz, S. Ligthart, T. Wang, J. A. Taylor, L. Duijts, G. C. Sharp, S. A. Jankipersadsing, R. M. Nilsen, A. Vaez, M. D. Fallin, D. Hu, A. A. Litonjua, B. F. Fuemmeler, K. Huen, J. Kere, I. Kull, M. C. Munthe-Kaas, U. Gehring, M. Bustamante, M. J. Saurel-Coubizolles, B. M. Quraishi, J. Ren, J. Tost, J. R. Gonzalez, M. J. Peters, S. E. H??berg, Z. Xu, J. B. Van Meurs, T. R. Gaunt, M. Kerkhof, E. Corpeleijn, A. P. Feinberg, C. Eng, A. A. Baccarelli, S. E. Benjamin Neelon, A. Bradman, S. K. Merid, A. Bergstr??m, Z. Herceg, H. Hernandez-Vargas, B. Brunekreef, M. Pinart, B. Heude, S. Ewart, J. Yao, N. Lemonnier, O. H. Franco, M. C. Wu, A. Hofman, W. McArdle, P. Van Der Vlies, F. Falahi, M. W. Gillman, L. F. Barcellos, A. Kumar, M. Wickman, S. Guerra, M. A. Charles, J. Holloway, C. Auffray, H. W. Tiemeier, G. D. Smith, D. Postma, M. F. Hivert, B. Eskenazi, M. Vrijheid, H. Arshad, J. M. Ant??, A. Dehghan, W. Karmaus, I. Annesi-Maesano, J. Sunyer, A. Ghantous, G. Pershagen, N. Holland, S. K. Murphy, D. L. Demeo, E. G. Burchard, C. Ladd-Acosta, H. Snieder, W. Nystad, G. H. Koppelman, C. L. Relton, V. W. V Jaddoe, A. Wilcox, E. Mel??n, S. J. London, DNA Methylation in Newborns and Maternal Smoking in Pregnancy: Genome-wide Consortium Meta-analysis, *Am. J. Hum. Genet.* **98**, 680–696 (2016).

66. K. S. Rønningen, L. Paltiel, H. M. Meltzer, R. Nordhagen, K. K. Lie, R. Hovengen, M. Haugen, W. Nystad, P. Magnus, J. A. Hoppin, The biobank of the Norwegian mother and child cohort Study: A resource for the next 100 years, *Eur. J. Epidemiol.* **21**, 619–625 (2006).

67. M. Bibikova, B. Barnes, C. Tsan, V. Ho, B. Klotzle, J. M. Le, D. Delano, L. Zhang, G. P. Schroth, K. L. Gunderson, J. B. Fan, R. Shen, High density DNA methylation array with single CpG site resolution, *Genomics* **98**, 288–295 (2011).

68. P. Magnus, L. M. Irgens, K. Haug, W. Nystad, R. Skjærven, C. Stoltenberg, E. Alsaker, L. S. Bakketeig, A. K. Daltveit, M. Eggesbø, J. Eide, B. Hareide, M. Haugen, R. Hovengen, K. K. Lie, R. T. Lie, H. M. Meltzer, R. Nilsen, R. Nordhagen, L. Paltiel, T. Reichborn-Kjennerud, K. S. Rønningen, P. Schreuder, N. Stensrud, A. Sunde, G. S. Tell, S. E. Vollset, J. Wiik, Cohort profile: The Norwegian Mother and Child Cohort Study (MoBa), *Int. J. Epidemiol.* **35**, 1146–1150 (2006).

69. A. J. Wilcox, R. T. Lie, K. Solvoll, J. Taylor, D. R. McConnaughey, F. Abyholm, H. Vindenes, S. E. Vollset, C. A. Drevon, Folic acid supplements and risk of facial clefts: national population based case-control study, *BMJ* **334**, 464–464 (2007).

70. Z. Xu, L. Niu, L. Li, J. A. Taylor, ENmix: a novel background correction method for Illumina HumanMethylation450 BeadChip, *Nucleic Acids Res.* **44**, e20–e20 (2016).

71. C. Hoyo, A. P. Murtha, J. M. Schildkraut, M. R. Forman, B. Calingaert, W. Demark-Wahnefried, J. Kurtzberg, R. L. Jirtle, S. K. Murphy, Folic acid supplementation before and during pregnancy in the Newborn Epigenetics STudy (NEST)., *BMC Public Health*

11, 46 (2011).

72. C. Hoyo, A. P. Murtha, J. M. Schildkraut, R. Jirtle, W. Demark-Wahnefried, M. R. Forman, E. S. Iversen, J. Kurtzberg, F. Overcash, Z. Huang, S. K. Murphy, Methylation variation at IGF2 differentially methylated regions and maternal folic acid use before and during pregnancy, *Epigenetics* **6**, 928–936 (2011).

73. P. Rantakallio, The longitudinal study of the Northern Finland birth cohort of 1966, *Paediatr. Perinat. Epidemiol.* **2**, 59–88 (1988).

74. U. Sovio, A. J. Bennett, L. Y. Millwood, J. Molitor, P. F. O'Reilly, N. J.Timpson, M. Kaakinen, J. Laitinen, J. Haukka, D. Pillas, I. Tzoulaki, J. Molitor, C. Hoggart, L. J. M. Coin, J. Whittaker, A. Pouta, A. L. Hartikainen, N. B. Freimer, E. Widen, L. Peltonen, P. Elliott, M. I.Mccarthy, M. R. Jarvelin, Genetic determinants of height growth assessed longitudinally from infancy to adulthood in the northern finland birth cohort 1966, *PLoS Genet.* **5** (2009), doi:10.1371/journal.pgen.1000409.

75. M. R. Jarvelin, A. -L Hartikainen-Sorri, P. Rantakallio, Labour induction policy in hospitals of different levels of specialisation, *BJOG An Int. J. Obstet. Gynaecol.* **100**, 310–315 (1993).

76. A. Jaaskelainen, U. Schwab, M. Kolehmainen, M. Kaakinen, M. J. Savolainen, P. Froguel, S. Cauchi, M. R. Jarvelin, J. Laitinen, Meal frequencies modify the effect of common genetic variants on body mass index in adolescents of the northern Finland birth cohort 1986, *World Rev. Nutr. Diet.* **109**, 10–11 (2014).

77. D. Gilbert-Diamond, K. L. Cottingham, J. F. Gruber, T. Punshon, V. Sayarath, A. J. Gandolfi, E. R. Baker, B. P. Jackson, C. L. Folt, M. R. Karagas, Rice consumption contributes to arsenic exposure in US women, *Proc. Natl. Acad. Sci.* **108**, 20656–20660 (2011).

78. G. Willemsen, J. M. Vink, A. Abdellaoui, A. den Braber, J. H. D. A. van Beek, H. H. M. Draisma, J. van Dongen, D. van 't Ent, L. M. Geels, R. van Lien, L. Ligthart, M. Kattenberg, H. Mbarek, M. H. M. de Moor, M. Neijts, R. Pool, N. Stroo, C. Kluft, H. E. D. Suchiman, P. E. Slagboom, E. J. C. de Geus, D. I. Boomsma, The Adult Netherlands Twin Register: Twenty-Five Years of Survey and Biological Data Collection, *Twin Res. Hum. Genet.* **16**, 271–281 (2013).

79. G. Willemsen, E. J. de Geus, M. Bartels, C. E. van Beijsterveldt, a I. Brooks, G. F. Estourgie-van Burk, D. a Fugman, C. Hoekstra, J. J. Hottenga, K. Kluft, P. Meijer, G. W. Montgomery, P. Rizzu, D. Sondervan, a B. Smit, S. Spijker, H. E. Suchiman, J. a Tischfield, T. Lehner, P. E. Slagboom, D. I. Boomsma, The Netherlands Twin Register biobank: a resource for genetic epidemiological studies, *Twin Res Hum Genet* **13**, 231–245 (2010).

80. M. J. Bonder, R. Luijk, D. V Zhernakova, M. Moed, P. Deelen, M. Vermaat, M. van Iterson, F. van Dijk, M. van Galen, J. Bot, R. C. Slieker, P. M. Jhamai, M. Verbiest, H. E. D. Suchiman, M. Verkerk, R. van der Breggen, J. van Rooij, N. Lakenberg, W. Arindrarto, S. M. Kielbasa, I. Jonkers, P. van 't Hof, I. Nooren, M. Beekman, J. Deelen, D. van Heemst, A. Zhernakova, E. F. Tigchelaar, M. A. Swertz, A. Hofman, A. G. Uitterlinden, R. Pool, J. van Dongen, J. J. Hottenga, C. D. A. Stehouwer, C. J. H. van der Kallen, C. G. Schalkwijk, L. H. van den Berg, E. W. van Zwet, H. Mei, Y. Li, M. Lemire, T. J. Hudson, P. E. Slagboom, C. Wijmenga, J. H. Veldink, M. M. J. van Greevenbroek, C. M. van Duijn, D. I. Boomsma, A. Isaacs, R. Jansen, J. B. J. van Meurs, P. A. C. 't Hoen, L. Franke, B. T. Heijmans, Disease variants alter transcription factor levels and methylation of their binding sites, *Nat. Genet.* **49**, 131–138 (2016).

81. J. Van Dongen, M. G. Nivard, G. Willemsen, J. Hottenga, Q. Helmer, C. V Dolan, E. A. Ehli, G. E. Davies, M. Van Iterson, C. E. Breeze, S. Beck, B. Consortium, H. E. Suchiman, R. Jansen, J. B. Van Meurs, B. T. Heijmans, P. E. Slagboom, D. I. Boomsma, Genetic and environmental influences interact with age and sex in shaping the human methylome, *Nat. Commun.* **7**, 1–13 (2016).

82. M. Van Iterson, E. W. Tobi, R. C. Slieker, W. Den Hollander, R. Luijk, P. E. Slagboom, B. T. Heijmans, MethylAid: Visual and interactive quality control of large Illumina 450k datasets, *Bioinformatics* **30**, 3435–3437 (2014).

83. A. H. Wijga, M. Kerkhof, U. Gehring, J. C. De jongste, D. S. Postma, R. C. Aalberse, A. P. H. Wolse, G. H. Koppelman, L. Van rossem, M. Oldenwening, B. Brunekreef, H. A. Smit, Cohort profile: The prevention and incidence of asthma and mite allergy (PIAMA) birth cohort, *Int. J. Epidemiol.* **43**, 527–535 (2014).

84. P. Girchenko, E. Hämäläinen, E. Kajantie, A.-K. Pesonen, P. M. Villa, H. Laivuori, K. Räikkönen, S. Entringer, C. Buss, P. D. Wadhwa, E. Hämäläinen, E. Kajantie, A.-K. Pesonen, P. M. Villa, H. Laivuori, K. Räikkönen, Prediction and Prevention of Preeclampsia and Intrauterine Growth Restriction (PREDO) study, *Int. J. Epidemiol.* , dyw154 (2016).

85. K. J. Brunst, R. O. Wright, K. DiGioia, M. B. Enlow, H. Fernandez, R. J. Wright, S. Kannan, Racial/ethnic and sociodemographic factors associated with micronutrient intakes and inadequacies among pregnant women in an urban US population, *Public Health Nutr.* **17**, 1960–1970 (2014).

86. M. M. De Carli, A. A. Baccarelli, L. Trevisi, I. Pantic, K. J. Brennan, M. R. Hacker, H. Loudon, K. J. Brunst, R. O. Wright, R. J. Wright, A. C. Just, Epigenome-wide cross-tissue predictive modeling and comparison of cord blood and placental methylation in a birth cohort, *Epigenomics* **9**, 231–240 (2017).

87. E. Oken, A. A. Baccarelli, D. R. Gold, K. P. Kleinman, A. A. Litonjua, D. De Meo, J. W. Rich-Edwards, S. L. Rifas-Shiman, S. Sagiv,

E. M. Taveras, S. T. Weiss, M. B. Belfort, H. H. Burris, C. A. Camargo, S. Y. Huh, C. Mantzoros, M. G. Parker, M. W. Gillman, Cohort profile: Project viva, *Int. J. Epidemiol.* **44**, 37–48 (2015).

88. J. P. Newnham, S. F. Evans, C. A. Michael, F. J. Stanley, L. I. Landau, Effects of frequent ultrasound during pregnancy: a randomised controlled trial, *Lancet* **342**, 887–891 (1993).

89. R. C. Huang, T. A. Mori, L. J. Beilin, Early life programming of cardiometabolic disease in the Western Australian pregnancy cohort (Raine) study, *Clin. Exp. Pharmacol. Physiol.* **39**, 973–978 (2012).

90. Y. Assenov, F. Müller, P. Lutsik, J. Walter, T. Lengauer, C. Bock, Comprehensive analysis of DNA methylation data with RnBeads, *Nat. Methods* **11**, 1138–1140 (2014).

91. J.-P. Fortin, E. Fertig, K. Hansen, shinyMethyl: interactive quality control of Illumina 450k DNA methylation arrays in R, *F1000Research* (2014), doi:10.12688/f1000research.4680.2.

92. A. A. Appleton, M. A. Murphy, D. C. Koestler, C. Lesseur, A. G. Paquette, J. F. Padbury, B. M. Lester, C. J. Marsit, Prenatal Programming of Infant Neurobehaviour in a Healthy Population, *Paediatr. Perinat. Epidemiol.* **30**, 367–375 (2016).

93. C. Almqvist, A. K. Ortqvist, V. Ullemar, C. Lundholm, P. Lichtenstein, P. K. E. Magnusson, Cohort Profile: Swedish Twin Study on Prediction and Prevention of Asthma (STOPPA), *Twin Res. Hum. Genet.* **18**, 273–280 (2015).

94. H. Anckarsater, S. Lundstrom, L. Kollberg, N. Kerekes, C. Palm, E. Carlstrom, N. Langstrom, P. K. Magnusson, L. Halldner, S. Bolte, C. Gillberg, C. Gumpert, M. Rastam, P. Lichtenstein, The Child and Adolescent Twin Study in Sweden (CATSS), *Twin Res. Hum. Genet.* **14**, 495–508 (2011).

95. J. Zetterqvist, A. Sjölander, Doubly Robust Estimation with the R Package drgee, *Epidemiol. Method.* **4**, 69–86 (2015).

96. S. E. Moore, A. J. Fulford, M. K. Darboe, M. L. Jobarteh, L. M. Jarjou, A. M. Prentice, A randomized trial to investigate the effects of pre-natal and infant nutritional supplementation on infant immune development in rural Gambia: the ENID trial: Early Nutrition and Immune Development., *BMC Pregnancy Childbirth* **12**, 1–8 (2012).
