# Supplementary material for: Meta-analysis of epigenome-wide association studies in neonates reveals widespread differential DNA methylation associated with birthweight
Source: Nat Commun. 2019 Apr 23;10:1893. doi: 10.1038/s41467-019-09671-3 (PMC6478731; doi:10.1038/s41467-019-09671-3)
Supplement: Supplementary file 3 — Description of Additional Supplementary Files [file 41467_2019_9671_MOESM3_ESM.pdf]

Description of Additional Supplementary Files

Supplementary Data 1. Characteristics of the participating studies in the main meta-analysis (A), the look-up in blood samples taken later in life (B) and the sensitivity model without exclusions of preterm births, pre-eclampsia or maternal diabetes (C)

Supplementary Data 2. Main meta-analysis results from 8696 CpGs with FDR siginificant association between methylation levels and continuous birth weight in grams (first 1071 CpGs are Bonferroni significant, P<1.06*10-7)

Supplementary Data 3. Meta-analysis results from 914 CpGs for the association between methylation levels and continuous birth weight in grams, in three separate ancestry groups

Supplementary Data 4. Meta-analysis results from 51 CpGs with association between methylation levels and high versus normal birth weight, after excluding preterm births, pre-eclampsia and maternal diabetes (P<1.06*10-7)

Supplementary Data 5. Results for meta-analyses without exclusion of preterm births, pre-eclampsia or maternal diabetes; association of DNA methylation with continuous birthweight in grams (n=5414), low (n=178) versus normal (n=4197) birthweight, and high (n=1039) versus normal (n=4197) birthweight

Supplementary Data 6. Comparison of 914 robust CpGs with two lists of SNP-influenced CpG probes and results from the dip test for multimodality

Supplementary Data 7. Meta-analysis results between methylation levels and continuous birth weight in grams from look-up in methylation samples in childhood, adolescence and adulthood: results from 914 robust CpGs in main meta-analysis

Supplementary Data 8. Overlap of CpGs that are associated with birthweight as well as maternal smoking during pregnancy or maternal body mass index

Supplementary Data 9. Comparison of 914 birthweight-related CpG sites with metastable epialleles and CpGs associated with genomic imprinting

Supplementary Data 10. Birthweight-related CpGs sites that are located +/- 2Mb of birthweight-related SNPs from a recently published fetal GWAS meta-analysis (Horikoshi et al.)

Supplementary Data 11. Birthweight-related CpGs sites that are located +/- 2Mb of birthweight-related SNPs from a recently published maternal GWAS meta-analysis (Beaumont et al.)

Supplementary Data 12. The 98 expression quantitative trait methylation (cis-eQTM) sites for 82 of the 914 birthweight associated CpG sites

Supplementary Data 13. Associations between gene expression and methylation in INMA and the Gambia (FDR<0.05)

Supplementary Data 14. All 148 methylation quantitative trait loci for 126 of the 914 birthweight-related CpGs
