# Supplementary material for: Meta-analysis of epigenome-wide association studies in neonates reveals widespread differential DNA methylation associated with birthweight
Source: Nat Commun. 2019 Apr 23;10:1893. doi: 10.1038/s41467-019-09671-3 (PMC6478731; doi:10.1038/s41467-019-09671-3)
Supplement: Supplementary file 18 — Reporting Summary [file 41467_2019_9671_MOESM18_ESM.pdf]

nature research

Corresponding author(s): Janine Felix, Harold Snieder, Caroline Relton, Debbie Lawlor

Last updated by author(s): Feb 17, 2019

Reporting Summary

Nature Research wishes to improve the reproducibility of the work that we publish. This form provides structure for consistency and transparency in reporting. For further information on Nature Research policies, see Authors & Referees and the Editorial Policy Checklist.

Statistics

For all statistical analyses, confirm that the following items are present in the figure legend, table legend, main text, or Methods section.

n/a	Confirmed	
☐	☒	The exact sample size (*n*) for each experimental group/condition, given as a discrete number and unit of measurement
☐	☒	A statement on whether measurements were taken from distinct samples or whether the same sample was measured repeatedly
☐	☒	The statistical test(s) used AND whether they are one- or two-sided<br>*Only common tests should be described solely by name; describe more complex techniques in the Methods section.*
☐	☒	A description of all covariates tested
☐	☒	A description of any assumptions or corrections, such as tests of normality and adjustment for multiple comparisons
☐	☒	A full description of the statistical parameters including central tendency (e.g. means) or other basic estimates (e.g. regression coefficient) AND variation (e.g. standard deviation) or associated estimates of uncertainty (e.g. confidence intervals)
☐	☒	For null hypothesis testing, the test statistic (e.g. *F*, *t*, *r*) with confidence intervals, effect sizes, degrees of freedom and *P* value noted<br>*Give P values as exact values whenever suitable.*
☒	☐	For Bayesian analysis, information on the choice of priors and Markov chain Monte Carlo settings
☐	☒	For hierarchical and complex designs, identification of the appropriate level for tests and full reporting of outcomes
☐	☒	Estimates of effect sizes (e.g. Cohen's *d*, Pearson's *r*), indicating how they were calculated

Our web collection on statistics for biologists contains articles on many of the points above.

Software and code

Policy information about availability of computer code

Data collection	All analyses were performed in R, except for the meta-analysis, which was performed using METAL. The code used for this EWAS meta-analysis is available from the corresponding authors upon reasonable request.
Data analysis	All analyses were performed in R, except for the meta-analysis, which was performed using METAL. The code used for this EWAS meta-analysis is available from the corresponding authors upon reasonable request.

For manuscripts utilizing custom algorithms or software that are central to the research but not yet described in published literature, software must be made available to editors/reviewers. We strongly encourage code deposition in a community repository (e.g. GitHub). See the Nature Research guidelines for submitting code & software for further information.

Data

Policy information about availability of data

All manuscripts must include a data availability statement. This statement should provide the following information, where applicable:

- Accession codes, unique identifiers, or web links for publicly available datasets
- A list of figures that have associated raw data
- A description of any restrictions on data availability

All relevant data supporting the key findings of this study are available within this article and its Supplementary Information files or from the corresponding authors upon reasonable request. All summary statistics from this EWAS meta-analysis are available via doi: 10.5281/zenodo.2222287.

Field-specific reporting

Please select the one below that is the best fit for your research. If you are not sure, read the appropriate sections before making your selection.

☒ Life sciences ☐ Behavioural & social sciences ☐ Ecological, evolutionary & environmental sciences

For a reference copy of the document with all sections, see nature.com/documents/nr-reporting-summary-flat.pdf

Life sciences study design

All studies must disclose on these points even when the disclosure is negative.

Sample size	To the best of our knowledge, we included the largest available sample size at the time of analysis, which was 8,825 individuals, in our main analysis.
Data exclusions	We pre-defined exclusion of multiple births, preterm births, and offspring of mothers with pre-eclampsia or diabetes from our main analyses, as the latter three are major pathological cause of differences in fetal growth. As a secondary analysis, we reran our primary analyses without exclusion of women with preeclampsia, diabetes or preterm delivery. We excluded DNA methylation probes that did not pass quality control, as well as control probes and probes mapping to the X and Y chromosomes.
Replication	To increase the power for discovery, we included all available studies in the primary meta-analysis. We then explored heterogeneity between studies within the meta-analysis and focused our follow-up analyses on those differentially methylated CpGs that did not show large between-study heterogeneity.
Randomization	There was no randomization, as this is a meta-analysis of observational studies.
Blinding	There was no blinding, this is not relevant for our study.

Reporting for specific materials, systems and methods

We require information from authors about some types of materials, experimental systems and methods used in many studies. Here, indicate whether each material, system or method listed is relevant to your study. If you are not sure if a list item applies to your research, read the appropriate section before selecting a response.

Materials & experimental systems

n/a	Involved in the study	
☒	☐	Antibodies
☒	☐	Eukaryotic cell lines
☒	☐	Palaeontology
☒	☐	Animals and other organisms
☐	☒	Human research participants
☒	☐	Clinical data

Methods

n/a	Involved in the study	
☒	☐	ChIP-seq
☒	☐	Flow cytometry
☒	☐	MRI-based neuroimaging

Human research participants

Policy information about studies involving human research participants

Population characteristics	We used data from 8,825 neonates from 24 studies, representing mainly European, but also African and Hispanic ethnicities with similar proportions of males and females.
Recruitment	The subjects were participants in 24 observational studies. Details for each of the 24 participating studies are provided in the Supplementary Methods.
Ethics oversight	All studies received approval from local ethics committees. Study-specific ethical approval statements are provided in the Supplementary Methods.

Note that full information on the approval of the study protocol must also be provided in the manuscript.
